# Exploring a Potential Optimization Route for Peptide Ligands of the Sam Domain from the Lipid Phosphatase Ship2

**DOI:** 10.3390/ijms251910616

**Published:** 2024-10-02

**Authors:** Marian Vincenzi, Flavia Anna Mercurio, Sara La Manna, Rosanna Palumbo, Luciano Pirone, Daniela Marasco, Emilia Maria Pedone, Marilisa Leone

**Affiliations:** 1 Institute of Biostructures and Bioimaging, Via Pietro Castellino 111, 80131 Naples, Italy; marian.vincenzi@ibb.cnr.it (M.V.); flaviaanna.mercurio@cnr.it (F.A.M.); rosanna.palumbo@cnr.it (R.P.); luciano.pirone@cnr.it (L.P.); daniela.marasco@unina.it (D.M.); empedone@unina.it (E.M.P.); 2 Department of Pharmacy, University of Naples “Federico II”, Via Domenico Montesano 49, 80131 Naples, Italy; sara.lamanna@unina.it

**Keywords:** anticancer molecular tools, EphA2 receptor, Ship2 lipid phosphatase, Sam (Sterile alpha motif) domain, drug design, peptides

## Abstract

The Sam (Sterile alpha motif) domain of the lipid phosphatase Ship2 (Ship2-Sam) is engaged by the Sam domain of the receptor tyrosine kinase EphA2 (EphA2-Sam) and, this interaction is principally linked to procancer effects. Peptides able to hinder the formation of the EphA2-Sam/Ship2-Sam complex could possess therapeutic potential. Herein, by employing the FoldX software suite, we set up an in silico approach to improve the peptide targeting of the so-called Mid Loop interface of Ship2-Sam, representing the EphA2-Sam binding site. Starting from a formerly identified peptide antagonist of the EphA2-Sam/Ship2-Sam association, first, the most stabilizing mutations that could be inserted in each peptide position were predicted. Then, they were combined, producing a list of potentially enhanced Ship2-Sam ligands. A few of the in silico generated peptides were experimentally evaluated. Interaction assays with Ship2-Sam were performed using NMR and BLI (BioLayer Interferometry). In vitro assays were conducted as well to check for cytotoxic effects against both cancerous and healthy cells, and also to assess the capacity to regulate EphA2 degradation. This study undoubtedly enlarges our knowledge on how to properly target EphA2-Sam/Ship2-Sam associations with peptide-based tools and provides a promising strategy that can be used to target any protein–protein interaction.

## 1. Introduction

Ship2 (SH2 domain containing inositol 5-phosphatase 2) is a lipid phosphatase that principally dephosphorylates phosphatidylinositol 3,4,5-trisphosphate (PtdIns(3,4,5)P(3)) into phosphatidylinositol 3,4-bisphosphate (PtdIns(3,4)P(2)) and is also linked to the insulin signaling pathway in vivo [1,2]. Ship2 overexpression has been related to diverse pathological conditions, including metabolic disorders and Alzheimer’s disease [3]. Moreover, Ship2 has been associated with several tumor types and it has been speculated to have involvement in the metastasis mechanism of carcinoma cells [3]. High levels of Ship2 have been encountered in certain breast cancer cell lines and tissue, where Ship2 knockdown induces decreased AKT-phosphorylation, as well as reduced tumorigenesis in vivo and cell proliferation in culture [4]. However, the role of Ship2 in cancer onset and progression is debated and depends on the cancer type; for example, regarding glioblastoma, high Ship2 levels correlate with a reduction in AKT phosphorylation [4]. A connection has also been established between Ship2 and the tyrosine kinase receptor EphA2, which represents a target in the anticancer drug discovery field. Indeed, the EphA2 receptor is overexpressed in several tumor types and is involved in complex signaling related to the balance of an anti-oncogenic ephrin ligand-dependent path and a pro-oncogenic ligand independent path [5,6]. Ship2 works as a negative regulator of ligand-induced receptor endocytosis. This is followed by receptor degradation, and can be, consequently, exploited to decrease procancer outcomes linked to high EphA2 levels [7]. Ship2 is able to regulate EphA2 endocytosis through a heterotypic Sam (Sterile alpha motif)–Sam interaction [8]. The binding of the Sam domain of Ship2 (Ship2-Sam) to the Sam domain of EphA2 (EphA2-Sam) has been very well characterized from the structural point of view using both solution NMR [8,9] and X-ray crystallography [10] techniques.

**Figure 1:**
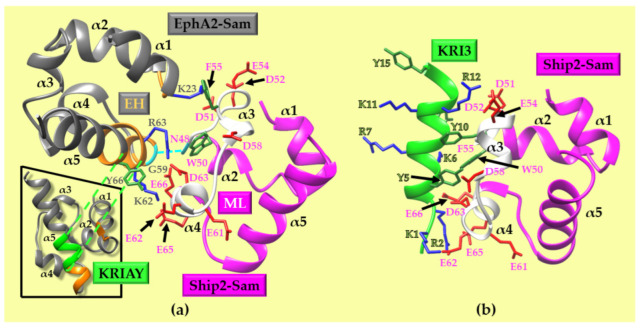
(**a**) The EphA2-Sam (gray)/Ship2-Sam (magenta) hetero-dimer. The EphA2-Sam EH interface (regions P58-Y66 and I22-M24 from the chain A of the PDB entry code 2KSO [9]) and the Ship2-Sam ML interface (segment H47-E66 from the chain B of the PDB entry code 2KSO) are colored orange and white, respectively. The side chains of aromatic, positively, and negatively charged residues present at the Sam–Sam EH/ML interface are shown in dark green, blue, and red, respectively. Residues G59 (EphA2-Sam) and N48 (Ship2-Sam) involved in the Sam–Sam characteristic H-bond (G59 _N_H/N48 O [14]) are colored in cyan and highlighted by a connecting dashed line. The inset shows the position of the KRIAY motif (green) within the EH interface of EphA2-Sam. (**b**) The docking model of the Ship2-Sam/KRI3 peptide complex [32]. The color code used for Ship2-Sam, aromatic, positively charged, and negatively charged residues is the same as that in panel (**a**). KRI3 is shown in a light green ribbon representation, with the side chains of Lys (blue), Arg (blue), and Tyr (dark green) reported in a neon representation.

Sam domains are small-protein interaction domains with a five-helix bundle fold; the Ship2-Sam/EphA2-Sam complex is a dimer, with a dissociation constant value in the low micromolar range, that follows a canonical Mid Loop (ML)–End Helix (EH) interaction model (Figure 1a) [11]. The Ship2-Sam ML binding site is rich in negatively charged residues and covers the Ship2-Sam central regions, while the EH interface in EphA2-Sam is positively charged and partially encompasses the C-terminal α5 helix and the close α1α2 loop (Figure 1a). A few aromatic residues are also present at the Sam–Sam interface, but the electrostatic interactions are the major contributors to complex formation [8]. It is thought that Sam domains containing a glycine at the N-terminal side of α5, like EphA2-Sam, have a tendency to participate in Sam–Sam interactions by proving the EH interface (Figure 1a); this glycine is an important anchorage point for Sam–Sam associations, establishing a crucial H-bond with a residue on the C-terminal side of α2 in the ML interface (Figure 1a) [12,13,14].

The recruitment of Ship2-Sam by EphA2-Sam should primarily cause pro-oncogenic outcomes in tumor cells. Thus, the development of peptide antagonists of heterotypic Sam–Sam associations, mediated by EphA2, represents a possible approach to the generation of novel anticancer agents.

Targeting Sam–Sam interactions is not a trivial task due to the large and flat interaction surfaces involved [9]. To date, many different well-established approaches have been exploited to design peptide modulators of protein–protein interactions (PPIs). The most common methods are those relying on virtual screenings of in silico generated peptide libraries against the target of interest through docking-like approaches. In this case, docking scores can be employed for peptide ranking and the prediction of the best ligands. However, docking approaches often do not contemplate the dynamic features that can characterize a protein/peptide interaction and are unsuccessful when a protein undergoes large conformational changes upon binding to a peptide [15]. Indeed, to save time and computational resources, completely or partially rigid ligands are frequently employed, providing just a static and often unreal picture of protein/peptide interactions [16]. This issue seemed to be overcome in a very recent multistep design strategy that made use of sophisticated molecular dynamics (MD) simulation methods to develop peptide modulators of the interaction between HVEM (herpesvirus entry mediator) and its interactor LIGHT. The HVEM/LIGHT complex plays a role in the immune system and can be considered a therapeutic target for the treatment of immune-related pathologies. This strategy integrated multi-trajectory all-atom MD simulations into the microsecond time scale with the evaluation of interaction energies through molecular mechanics with generalized Born and surface area (MMGBSA), steering MD simulations at various pulling speeds to identify promising peptide variants possessing high LIGHT affinity [15]. This MD-relying approach led to understand the conformational variations involved in LIGHT/HVEM complexing and pointed out the relevance of HVEM fragments and disulfide bonds to the protein/peptide recognition mechanism [15].

Nevertheless, artificial intelligence software mainly relying on deep learning approaches, like AlphaFold [17,18] and RosettaFold [19], has lately attracted a lot of attention. Such computational tools are focused on structure prediction. This is information highly relevant to peptide design and can consequently be used in diverse design pipelines (as very well described in a recent review article [20]). For example, AlphaFold2 has been exploited to support the design of cyclic peptide modulators of PPIs [21,22].

During the last years, to design peptides targeting the challenging Ship2-Sam/EphA2-Sam complex, we set up docking-based virtual screening strategies by employing the specific software and expertise available in our laboratory. For example, we investigated the possibility of targeting a region of EphA2-Sam that is important for engaging the ML interfaces of partner Sam domains with cyclic peptides through virtual screening, using AutoDock Vina [23], of peptide libraries containing diverse D- and L-amino acids in several peptide positions [14]. We also used a similar AutoDock Vina-based virtual screening approach to discover linear peptides targeting the EphA2-Sam EH site, starting from a fragment of the ML interface of Ship2-Sam and again generating a peptide library by inserting diverse D- and L-amino acids [24].

More recently, we set up a rather successful virtual screening approach in order to optimize a formerly identified Ship2-Sam ligand (i.e., the KRI3 peptide) [25], which acts as a weak inhibitor of the EphA2-Sam/Ship2-Sam association [26], by employing the Haddock Refinement Interface [25,27].

Herein, we further attempted the optimization of the KRI3 peptide through the FoldX (v. 5) [28,29] software suite. FoldX is a well-established tool that performs better than other software in predicting variations in Gibbs free energies (ΔΔGs) [30,31]. Our approach relies on the evaluation of both folding and binding ΔΔGs related to the starting KRI3 peptide and many mutant variants in complexes with Ship2-Sam. The best in silico obtained peptide and a few analogs were experimentally analyzed by an array of techniques, including NMR and CD measurements used for conformational studies, NMR and BLI interaction assays, and cell-based experiments.

## 2. Results and Discussion

### 2.1. Computational Approach for Peptide Design and Selection

The KRI3 peptide was previously developed in our laboratories (Figure 1b). The KRI3 amino acid sequence (Ac-KRIAYKRIAYKRIAY-NH_2_, where Ac and NH_2_ represent N-terminal acetylation and C-terminal amidation, respectively) includes the triple repetition of the “KRIAY” motif belonging to the EphA2-Sam primary sequence, and is placed along the EH interface (Figure 1b) [26]. The KRI3 peptide is a Ship2-Sam interactor (K_D_ equal to ~100 µM) that, by targeting the ML interface in Ship2-Sam, works as a weak antagonist of the EphA2-Sam/Ship2-Sam association [26].

A 3D model of the Ship2-Sam/KRI3 complex was built via docking studies (Figure 1b) [27]. Docking poses are mainly stabilized by networks of electrostatic interactions, mediated by Lys and Arg residues in KRI3, with Asp and Glu from the ML interface of Ship2-Sam and some π–π contact mediated by the Tyr residues (KRI3) with Phe and Trp (Ship2-Sam) (Figure 1b) [32].

**Figure 2:**
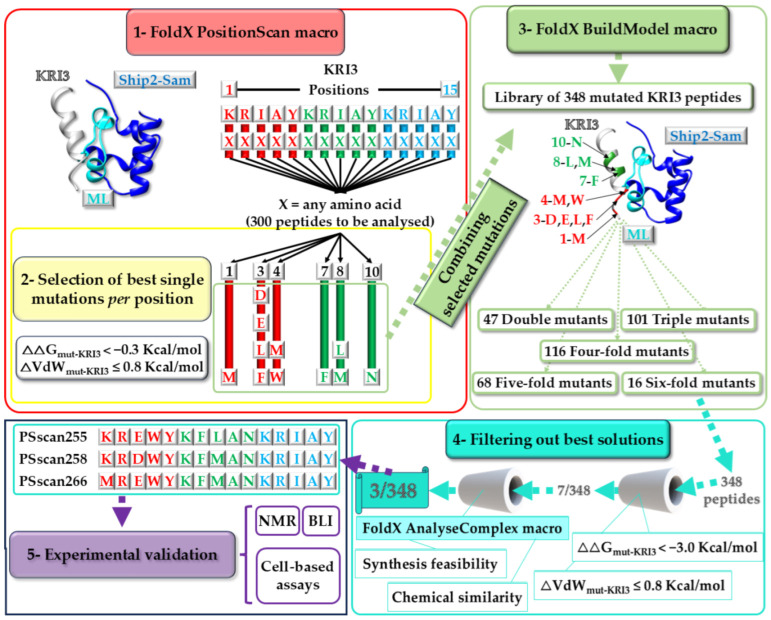
In silico design of peptide sequences through the different macros of FoldX (v. 5) [29]; the peptide selection strategy and the experimental in vitro evaluation protocol are also indicated.

Herein, we developed a computational strategy to generate novel peptides that could interact with the ML interface of Ship2-Sam, starting from the best docking model of the KRI3/Ship2-Sam complex (Figure 2). The approach was set up using the FoldX software package [28,29].

Interestingly, FoldX represents a clever computational instrument that can be used to evaluate the Gibbs free energy of macromolecules through the usage of an empirical force field [28,29]. The software can be, for example, employed to evaluate differences in free energies (=ΔΔG values) between mutant and wild-type variants. Stabilizing mutations are associated with more negative ΔΔG values, whereas positive ΔΔG values point out destabilizing amino acid substitutions [33].

Our approach relied mainly on three software macros. First, the macro “PositionScan” [33,34,35] was used to screen the whole KRI3 amino acid sequence, mutate each peptide position with all other 19 amino acids, and predict the most favorable amino acid substitutions (Figure 2, Box 1). The ΔΔG values, which were computed from the difference between the free energy of folding of the designed mutant and that of the WT (wild-type), were analyzed to evaluate the change in stability of the KRI3 peptide variants in complex with Ship2-Sam (ΔG_mut_) with respect to the unmutated reference structure (ΔG_WT_). The most favorable single-amino acid substitutions (=11 mutations spread over 6 KRI3 positions) were chosen while also considering ΔΔG values lower than −0.3 Kcal/mol and keeping into account low energy penalties due to Van der Waals clashes (i.e., ΔVdW ≤ 0.8 Kcal/mol) (Table S1 and Figure 2, Box 2). Next, these selected single mutations were combined to generate additional KRI3 mutant peptides by implementing the “BuildModel” macro of FoldX [36,37] (Figure 2, Box 3). Overall, 348 KRI3 variants containing multiple amino acid substitutions (from 2 to 6 mutations) were built and analyzed within this second step (Figure 2, Box 3). Based on ΔΔG folding estimates and energy penalties due to Van der Waals (VdW) clashes (established criteria ΔΔG_mut-KRI3_ < −3 Kcal/mol and ΔVdW_mut-KRI3_ ≤ 0.8 Kcal/mol), 7 KRI3 variants were selected (Table S2 and Figure 2, Box 4).

In the final step of our strategy, the 7 selected peptides in complex with Ship2-Sam were further inspected with the “AnalyseComplex” macro of FoldX that performs the computational evaluation of binding affinities [28,29,38]. In detail, differences in binding affinities [38] and penalties due to Van der Waals clashes between Ship2-Sam/mutant peptide complexes and the reference Ship2-Sam/KRI3 peptide complexes, generated at the previous step by the “BuildModel” macro, were computed (Table S3). The best peptide in terms of interaction energy was PSscan255. Differences between intramolecular Van der Waals’ clashes among residues at the interaction interface of either Ship2-Sam or different mutant peptides and those in the Ship2-Sam/KRI3 reference complexes indicated that PSscan258 and PSscan266 presented the lowest steric clashes at the protein or peptide interfaces, respectively, and were consequently, chosen for further experimental analyses (Table S3). Interestingly, although PSscan266 and PSscan258 possess high sequence similarity with PSscan255 with only two additional mutations (i.e., K1M and L8M for PSscan266 and E3D, L8M for PSscan258) (Table S4), they are predicted to have non-optimal interaction energies (Table S3) and, thus, represent interesting peptides for the experimental validation of the proposed computational protocol. In the end, following the analysis of the parameters provided by the “AnalyseComplex” macro (i.e., intramolecular Van der Waals penalties and interaction energies), and upon extra evaluation of sequence similarities and synthetic chemistry efforts, 3/7 optimized peptide sequences (i.e., PSscan255, PSscan258 and PSscan266) (Table S3 and Figure 2, Box 4), containing either 5 (PSscan255 and PSscan258) or 6 (PSscan266) amino acid substitutions with respect to the KRI3 starting sequence, were retrieved for experimental studies (Figure 2, Box 5).

### 2.2. Peptide Structural Characterization

Conformational analyses of PSscan255, PSscan258, and PSscan266 peptides were achieved through CD and NMR investigations (Figures S1–S5).

#### 2.2.1. CD Studies

CD spectra were recorded in sodium phosphate by performing a TFE titration (0–80%, *v*/*v*) (Figure S1), and their deconvolution is reported in Figure S2. In the absence of TFE, the CD spectrum suggests combined random-beta conformations, by exhibiting two minima centered at ~202 and 218 nm (Figure S1). The addition of TFE led to a certain increase in the helical content, as indicated by the shift in the distinct minima at 209 and 223 nm. This tendency is more evident for PSscan255 and PSscan266 (Figure S2).

#### 2.2.2. NMR Studies

Two-dimensional (2D) [^1^H, ^1^H] NOESY (Nuclear Overhauser Enhancement Spectroscopy) [39] and 2D [^1^H, ^1^H] ROESY (Rotating-Frame Overhauser Enhancement Spectroscopy) [40] spectra of PSscan peptides were first recorded in a PBS buffer and indicated flexible and mainly disordered conformations (Figures S3a–S5a).

**Figure 3:**
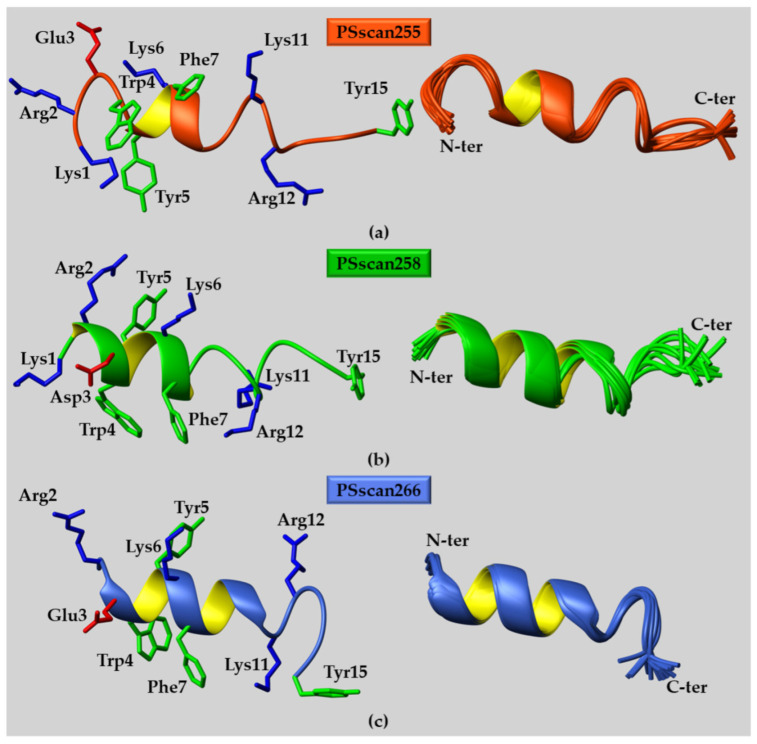
NMR solution structures in PBS/TFE (50/50—*v*/*v*) of (**a**) PSscan255; (**b**) PSscan258; and (**c**) PSscan266 peptides. (**a**–**c**) Representative NMR conformers are reported in the left panels in ribbon drawings, whereas superpositions on the backbone atoms of twenty structures are shown in the right panels. In the left panels, diverse charged and aromatic residues are evidenced in a neon representation with only heavy atoms. (**a**) PSscan255 conformers were calculated from 175 distance restraints (52 intra-residue, 53 short-, 70 medium-, and 0 long-range) and 81 angle constraints; (**b**) regarding PSscan258, 159 distance restraints (50 intra-residue, 57 short-, 52 medium-, and 0 long-range) along with 83 angle constraints were employed to derive the NMR structure; (**c**) the PSscan266 structure was calculated based on 177 distance restraints (74 intra-residue, 54 short-, 49 medium-, and 0 long-range) and 81 angle constraints.

On the contrary, peptide solution structures could be calculated in PBS/TFE (50/50—*v*/*v*) mixtures due to the improved signal dispersion of NMR spectra (Figures S3b–S5b) and enlarged amount of NOE/ROE contacts that could be observed (Tables S5–S10 and Figure 3 and Figure S6).

Comparative analyses of TOCSY and NOESY experiments allowed us to collect most proton resonance assignments for PSscan255, PSscan258, and PSscan266 (Tables S5–S7) [41]. Chemical shift deviations of Hα protons with respect to random coil values were calculated [42,43]. The results highlighted the rise in helical conformations, covering mostly the regions from residue 4 to 12, in the three peptides in solution containing 50% TFE (Figure S6a–c left panels). In line with these results, the pattern of short- and medium-range NOEs for all peptides also showed a relevant number of Hα_i_-Hβ_i+3_ correlations, canonical of helical structuration [41] (Figure S6a–c right panels). Three-dimensional (3D) structures of PSscan255, 258 and 266 were calculated with the software CYANA 2.1 [44] (Figure 3 and Tables S8–S10). The inspection of PSscan255 conformers with the software MOLMOL 2K.2 [45] revealed a more ordered α-helical turn, encompassing the segment Y5-L8, flanked by disordered bends/turns (regions E3-W4 and A9-R12) (Figure 3a). For PSscan258, a regular α-helical structuration was present in the region between R2-M8 with a more disordered bend configuration at the C-terminal side from A9 to I13 (Figure 3b). Finally, for PSscan266, the regular α-helical segment covered residues from E3 to N10, while the C-terminal side from K11 to I13 presented a distorted (i.e., bend) helical organization (Figure 3c). The helical conformations in each peptide were stabilized, principally by π–π contacts and cation–π contacts in between residues in the positions i and i + 3/i + 4, like K1/R2 with Y5, W4 with F7, and F7 with K11 (as shown in Figure 3’s left panels).

### 2.3. Interaction Studies: Peptides vs. Ship2-Sam

#### 2.3.1. NMR Chemical Shift Perturbation Studies

**Figure 4:**
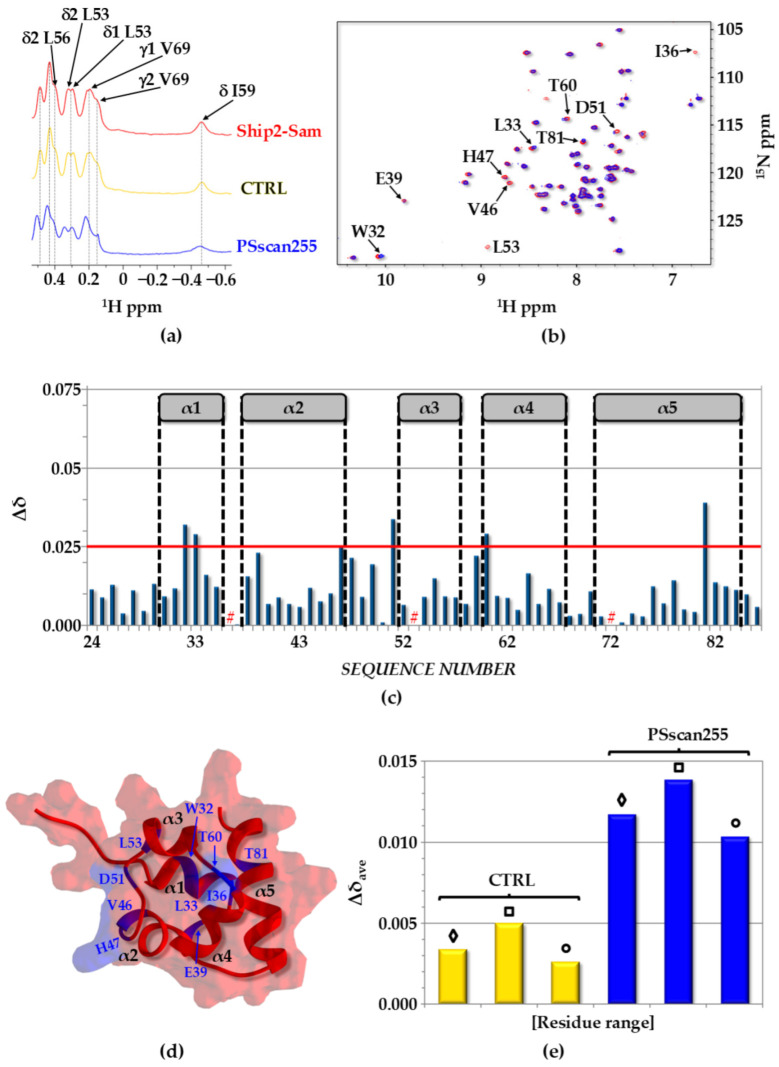
(**a**) Screening by 1D [^1^H] NMR. The expansion of aliphatic regions of 1D [^1^H] NMR spectra of Ship2-Sam in the apo form (27 µM concentration) (red) and in the presence of the different peptides (273 µM each). (**b**) Comparison of [^1^H-^15^N] HSQC spectra of Ship2-Sam (20 μM concentration) alone (red) and after the addition of the PSscan255 peptide (200 μM concentration) (blue). (**c**) Histogram showing chemical shift perturbations (CSPs) (i.e., Δδ = [(ΔH_N_)^2^ + (0.17 × Δ^15^N_H_)^2^]^1/2^) [49] versus residue numbers for the interaction between Ship2-Sam and PSScan255. The Δδ value was set to be equal to zero for P72 as well as I36 and L53 as their peaks disappear in the spectrum of the peptide/protein complex (See “#”). (**d**) Residues associated with the largest perturbations in chemical shifts (Δδ ≥ 0.025 ppm) or peak intensities (i.e., W32 (NHε1), L33, I36, E39, V46, H47, D51, L53, T60, T81) are highlighted in blue in the 3D solution structure of Ship2-Sam (conformer number 1, PDB entry code 2K4P [8]), which is displayed in the ribbon with a transparent surface drawing. (**e**) Average CSP (Δδ_ave_) values for control (CTRL) and PSscan255 peptides, which were evaluated for the entire Ship2-Sam sequence (“◊” residue range L24-K86), the ML interface (“□” protein segment H47-E66), and the region outside the ML (“○” residues L24-V46 and A67-K86). Peaks corresponding to backbone NH and side-chain NHε1 groups of W32 and W50 were included in the CSP evaluation.

The interaction between diverse PSscan peptides and Ship2-Sam was assessed by NMR chemical shift perturbation (CSP) studies using both 1D [^1^H] and 2D [^1^H-^15^N] HSQC experiments [46,47] (Figure 4 and Figures S7–S10).

As a positive control in our NMR studies, we used the KRI3 peptide (Figure S7) [26].

Instead, the peptide designated as “CTRL” (Ac-GPTRREDKFMYF-NH_2_), which can interact with the Sam domain of the tumor suppressor protein DLC1 (deleted in liver cancer), was used as a negative control peptide as it presents an amino acid sequence dissimilar from that of KRI3 [25,48]. Chemical shift perturbation experiments did not reveal high-affinity binding of CTRL to Ship2-Sam (Figure 4a, Figures S7 and S10).

Spectra recorded for Ship2-Sam alone and in the presence of PSscan255 (Figure 4a,b and Figure S10a) indicated small chemical shift changes and highlighted a weak interaction. Nevertheless, the mapping of the observed changes in the 3D structure of Ship2-Sam (Figure 4c,d) and the analysis of average chemical shift perturbations (Δδ_ave_) in diverse protein regions (Figure 4e and Figure S10b) clearly indicated a reduced specific binding to the ML interface with respect to the starting KRI3 peptide, for which larger changes were localized around the ML binding site for EphA2-Sam (Figure S10b).

In the case of PSscan255, chemical shift changes induced by the peptide affect, apart from a few residues within the Ship2-Sam ML interface, residues external to the EphA2-Sam binding site, like the side chain of Trp32 at the α1/α2 interface, which is located close to but not directly inside of the ML interface (Figure 4d). This region, as previously pointed out [26], could also be targeted by positively charged peptides as it contains clusters of negatively charged residues (Figure S11). However, it is possible that the PSscan255 peptide binds to one edge of the ML interface close to the α2 helix, thus inducing small conformational changes in the above-mentioned site.

To further evaluate unspecific peptide binding to Sam domains, chemical shift perturbation studies were also conducted with [^1^H-^15^N] HSQC spectra of ^15^N-labeled EphA2-Sam, which were recorded in the absence and in the presence of PSscan255, but no interaction could be detected at a protein/peptide concentration ratio equal to 1/10, indicating that the peptide presents a preference for binding Ship2-Sam (Figure S12).

Chemical shift perturbation studies performed with the PSscan258 (Figure S8) and PSscan266 (Figure S9) peptides again revealed weak interactions with Ship2-Sam. According to our NMR studies, the binding between PSscan258 and Ship2-Sam appeared the weakest, and spectra recorded for Ship2-Sam in the presence and in the absence of PSscan258 were indeed very similar to each other (Figures S8 and S10a).

As concerning PSscan266, the targeting of the ML interface seems not to be very specific (Figures S9 and S10a) and the peptide might as well bind laterally to the α1-α2 interface (Figure S11).

It is worth noting that NMR interaction data are in line with the in silico results from the “AnalyseComplex” macro of FoldX [28,29], indicating that the best ligand of this series may be PSscan255 (Table S3).

**Figure 5:**
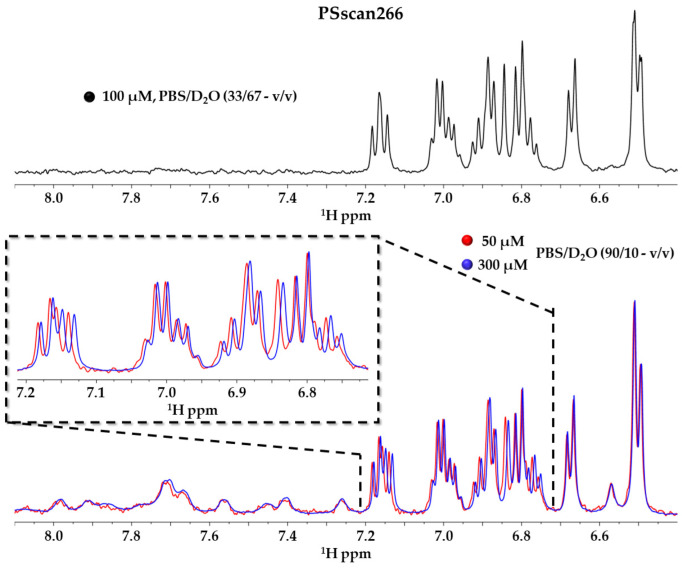
The 1D [^1^H] spectrum (HN/aromatic protons regions) of PSscan266 (100 µM concentration) in PBS/D_2_O (33/67—*v*/*v*) (black) is shown on the top. The overlay of 1D [^1^H] NMR spectra, recorded in PBS/D_2_O (90/10—*v*/*v*) of PSscan266 peptide at 50 µM (red) and 300 µM (blue) concentrations, is shown on the bottom. An expansion containing only peaks arising from aromatic protons is reported in the left inset. Peak intensities were adjusted to reach the same level in each spectrum.

In addition, to better understand results from NMR interaction assays, we evaluated whether peptide self-aggregation phenomena could occur in the peptide concentration range employed. Thus, NMR spectra were recorded for each PSscan peptide at diverse concentrations in the range of 50–300 µM (Figure 5, Figures S13 and S14). As all three peptides are rich in aromatic and positively charged residues, self-aggregation can occur not only through π–π stacking interactions but also through cation–π contacts between diverse peptide units. To better identify the signals of aromatic protons within 1D [^1^H] NMR spectra in PBS, hydrogen/deuterium exchange experiments were conducted (Figure 5 and Figure S14). Indeed, an excess of D_2_O in solution induces the disappearance of H_N_ and other exchangeable exposed protons, while aromatic and side-chain protons remain clearly visible (Figure 5 and Figure S14). When recording NMR spectra of peptides at different concentrations, particular care was also taken to keep the pH at a constant value to avoid undesired chemical shift changes.

Analyses of NMR experiments at diverse peptide concentrations clearly revealed that the only peptide that could be affected by a certain amount of self-aggregation was PSscan266 (Figure 5, upper panel). In fact, small chemical shift changes, particularly evident in the aromatic region of the spectra, indicated that some self-aggregation involving aromatic rings could occur (Figure 5, lower panel). However, at the concentration used to investigate binding to Ship2-Sam, due to the reduced line broadening observed in the NMR spectra, the presence of only small oligomeric species can be guessed (Figure 5).

It is worth noting that all PSscan peptides (Table S4) have similar amino acid sequences and contain one Trp residue, one Phe residue, and two Tyr residues. However, differently from PSscan255 and PSscan258, which have a positively charged Lys as the first N-terminal residue, PSscan266 contains a Met that can somehow improve the hydrophobic character and contribute to aggregation.

Additionally, to investigate intermolecular contacts, which could support peptide self-association, a 3D model of a PSscan266 dimer was built via docking studies through the Haddock webserver [27,50]. Based on the results of NMR experiments (Figure 5), docking runs were achieved by considering the aromatic residues as active. As CD data (Figures S1 and S2) indicated a mainly disordered/extended conformation for the peptide in an aqueous buffer and a helical organization in the presence of TFE, dimeric PSscan266 models were built for both the peptide in a β-organization and for the peptide in the helical structural arrangement observed in TFE. The best 10 docking models in terms of Haddock scores were analyzed for both peptide conformations (Figures S15 and S16). More similar Haddock solutions could be obtained for peptide self-association in an extended conformation, with a predominance of solutions where the two chains were arranged in an antiparallel orientation with respect to each other (Figure S15). A large degree of variability was observed in the two chains’ mutual orientation, starting from the helical PSscan266 conformation (Figure S16). The analysis of intermolecular contacts [51,52] between peptide chains in the best Haddock solution, performed using LigPlot+, revealed for the PSscan266 dimer in the extended configuration (Figure S17) a network of cation–π contact points and indicated that the negative side chain of the only Glu present in the peptide chain could also support self-association through electrostatic contacts with positively charged residues in another peptide unit if the chains were in an antiparallel arrangement. Regarding self-association mediated by an α-helical configuration, the analysis of intermolecular interactions in one of the best Haddock solutions indicates that, in this case also, a predominance of cation–π interactions could be present at the dimer interface and that the Glu residue is important for facilitating crucial intermolecular interactions (Figure S18). Intriguingly, LigPlot+ [51,52] analyses also pointed out that the first methionine is part of the interaction surface (Figures S17 and S18).

Peptide self-association in PSscan266 might be responsible for supporting an unspecific interaction with Ship2-Sam.

#### 2.3.2. Quantitative Assessment of the Binding between Ship2-Sam and PSscan255

Based on NMR investigations, the recognition of PSscan258 to Ship2-Sam appeared very weak, while PSscan266 demonstrated a certain propensity to self-associate; thus, only the PSscan255 peptide was evaluated regarding its affinity for Ship2-Sam through BLI assays.

The peptide was purchased in the biotinylated–pegylated form (Table S4) and immobilized on a streptavidin-coated biosensor. Kinetic experiments, carried out at diverse Ship2-Sam concentrations in the range of 20–500 µM (Figure S19), provided dose–response signals, allowing the estimation of a K_D_ value equal to (70 ± 40) µM.

#### 2.3.3. Docking Studies: PSscan255 vs. Ship2-Sam

**Figure 6:**
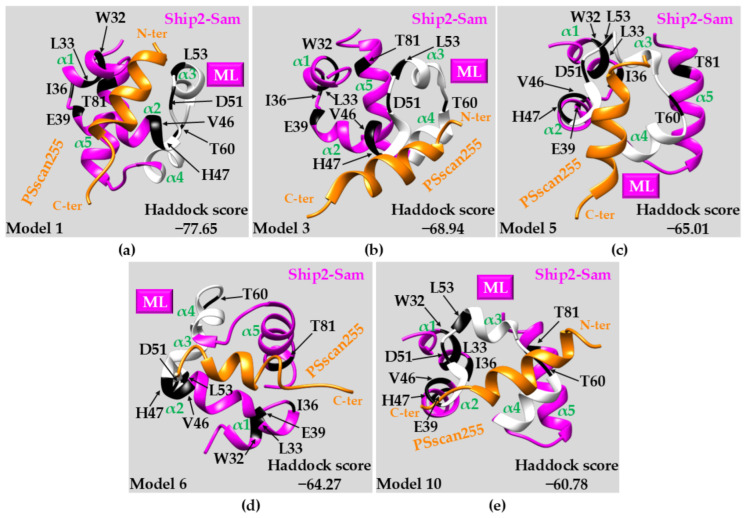
Docking results for the Ship2-Sam/PSscan255 peptide complex. In each panel, a different docking solution among the best 10—in terms of Haddock scores [50]—is shown. (**a**) 1st best model; (**b**) 3rd best model; (**c**) 5th best model; (**d**) 6th best model; (**e**) 10th best model. Each structure is reported in a ribbon representation: the ML region in Ship2-Sam (magenta) is colored white. The residues most affected by the binding of the peptide according to the NMR studies (i.e., W32 (NHε1), L33, I36, E39, V46, H47, D51, L53, T60, T81) are highlighted in black on the Ship2-Sam surface. The PSscan255 peptide is shown in an orange ribbon drawing. Different helices in Ship2-Sam are labeled.

Docking studies were conducted with the Haddock webserver and the NMR structures of Ship2-Sam and PSscan255 to gain further structural insights into the protein/peptide interaction mode [50]. A sort of blind docking was conducted, letting the peptide explore the whole protein surface (see the Section 3 for further information). The best 10 docking solutions in terms of Haddock scores were visually inspected, and after the exclusion of unrealistic poses with only limited contacts between peptide and protein, 5 models were analyzed more deeply (Figure 6 and Table S11). Most of these best docking solutions appeared to be compatible with the chemical shift perturbation data collected (Figure 6). The PSscan255 peptide in the helical conformation unveiled by TFE exhibits a higher propensity to occupy the ML binding site (Figure 6b,c,e and Figure S20a), although other regions are targeted, including the lateral side of the interface between α1 and α2 (Figure 6a and Figure S20b). This latter region represents the Ship2-Sam lateral site with respect to the ML interface (Figure S11), which is also affected by a few chemical shift perturbations; within this site, additional negatively charged residues (including E42 and E43) (Figure S11) are available to provide interactions with the positively charged residues of the PSscan255 peptide (Figure 6a and Figure S20b). In the best docking solutions analyzed, residues K1, R2, and K6 in the peptide provide the largest number of intermolecular H-bonds, followed by N10, K11, R12. Residues W4, F7 and L8 are involved in many non-bonded intermolecular contacts, whereas residues E3, I13, A14 and Y15 provide no interactions, revealing a possible dispensable role for the binding to Ship2-Sam (Table S11 and Figure S20). Results from docking studies indicate that optimized PSscan255 versions could be obtained by screening diverse amino acid types in the positions 3, 13, 14, and 15, which appear in the peptide sequence not relevant for binding to the protein. These could provide additional interactions if they are substituted by specific amino acids. Multidisciplinary approaches will be set up in the future to assess this point.

Recently, machine learning and artificial intelligence have been largely employed to accurately predict protein structures, either in isolated or complex forms, thus bringing tremendous changes to the field of structural biology. In this context, OmegaFold [53], AlphaFold [18,20], or RosettaFold [54] represent a few interesting recently developed tools for structure predictions. OmegaFold [53] is more appropriate for orphan proteins and antibodies for which reliable multiple sequence alignments of homologue proteins are unavailable due to evolutionary information being largely absent or unclear [53].

It has been predicted that AlphaFold and RosettaFold can even perform better than canonical docking approaches in the case of oligomeric models [54].

Thus, AlphaFold2 (AF2) [18,20] was employed to predict the structure of the PSscan255 peptide in complex with Ship2-Sam in order to verify whether results in line with those generated by Haddock could be obtained (Figure S21).

For the PSscan255/Ship2-Sam complex, AF2 predicted an interaction model very similar to what was observed in the Haddock Pose N. 10 (Figure 6e). Accordingly, AF2 predicted the ML interface of Ship2-Sam would be the peptide binding site (Figure S21). Moreover, in agreement with the NMR studies conducted in the mixture PBS/TFE, AF2 predicted a helical conformation for the peptide as well. The accuracy of the AF2 model was evaluated through the pLDDT (predicted local distance difference test) [17], pTM (predicted template modeling), and ipTM (interface pTM) scores [55,56]. Generally, pLDDTs larger than 80 can be associated with rather good predictions [57,58]; pTM and ipTM scores are linked to the global quality of the protein/peptide complexes or just their interfaces, respectively [59]. pTM and ipTM scores higher than 0.5 and 0.8, respectively, can be associated with accurate predictions [59]. The three accuracy scores appear non-optimal for the AF2 model of PSscan255/Ship2-Sam complex. Specifically, the ipTM value indicates the uncertainty of the AF2 prediction in establishing the exact positioning of the PSscan255 peptide within the ML interface of Ship2-Sam (Figure S21).

### 2.4. Cell-Based Experiments with PSscan255

In vitro cell-based assays were performed to assess cytotoxic effects and the possible modulation of ephrin ligand-induced EphA2 receptor degradation, associated with the PSscan255 peptide. Cell-based studies were mainly conducted in the prostate cancer cell-line PC-3 [60].

#### 2.4.1. Assessment of Serum Stability

The serum stability of the PSscan255 peptide was assessed by following the decrease in the chromatographic peak of the pure PSscan255 peptide over time upon incubation with FBS (fetal bovine serum) (Figures S22a and S23). The peptide appeared rather stable as, after 5 h, 80% could still be recovered, whereas after 19 h, 50% of the peptide was not degraded. This PSscan255 peptide sequence appears more stable in FBS with respect to the parent KRI3 peptide which, as we have previously reported, is almost completely degraded after ~17 h [32]. This outcome might be related to a lower content of positively charged residues (i.e., 5 in PSscan255 and 6 in KRI3) [61,62].

#### 2.4.2. Cellular Uptake

To allow cellular uptake, the PSscan255 sequence was linked to a TAT cell-penetrating motif [26] and, to further examine internalization in PC-3 cells by fluorescence microscopy, TAT-PSscan255 was also conjugated to a fluorescent moiety (fluorescein isothiocyanate (FITC)) (Table S4) (Figure S22b). The images obtained by fluorescence microscopy show that 4 h after treatment, FITC-TAT-PSscan255 was positioned in the cytoplasm (Figure S22b).

#### 2.4.3. Cytotoxicity

**Figure 7:**
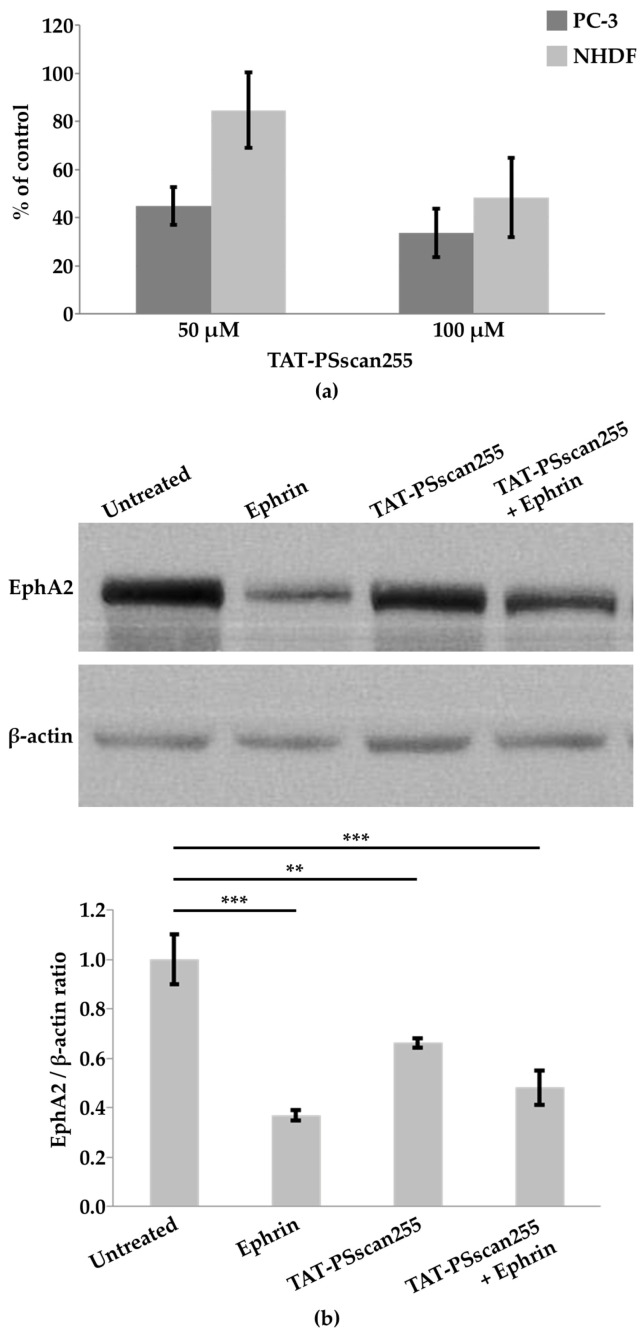
(**a**) The cytotoxic effect of PSscan255 peptide on PC-3 and NHDF cells was assessed through the crystal violet assay. Cells were treated with TAT-PSscan255 (50 μM and 100 μM concentrations for 4 h), and values were expressed as percentages relative to untreated cells. Each value represents an average ± SEM (Standard Error of Mean) of three separate experiments performed in quadruplicate. (**b**) EphA2 degradation in the prostatic cancer cell line. (Top) PC-3 cells were treated either with TAT-PSscan255 (50 μM for 4 h) and/or ephrinA1-Fc (1 μg/mL for 2 h). The β-actin antibody was employed for the comparison of protein loads. Characteristic data are presented (bottom). EphA2/β-actin ratios were normalized, assuming the EphA2 expression under the untreated condition as 1. Mean ± SEM, n = 3. One-way analysis of variance (ANOVA) using Dunnett’s post-test analysis was performed; ** *p*< 0.01; *** *p*< 0.001.

The cytotoxicity of TAT-PSscan255 was evaluated in PC-3 and in healthy cells (i.e., normal human dermal fibroblasts (NHDFs)) at 50 and 100 µM concentrations following 4 h treatments. Cell viability assays demonstrated an increased cytotoxic effect as a function of concentration and increased cytotoxicity towards PC-3 with respect to NHDFs, as already revealed for the parent KRI3 peptide [26] (Figure 7a).

#### 2.4.4. Western Blot Analyses to Monitor EphA2 Levels

In many tumors, EphA2 is present in high levels and with non-canonical functions [63]. The EphA2 ligand-dependent pathway is considered anti-oncogenic; upon binding to the ephrin ligand, EphA2 molecules form clusters, an activation process takes place, and signaling connected to reductions in cell migration and invasion occurs [64,65]. Interestingly, following ligand stimulation, EphA2 is internalized and degraded, and this endocytosis process holds importance by decreasing EphA2 overexpression in tumor cells and the related oncogenic pathway [63,66].

The levels of EphA2 were monitored in PC-3 cells through Western blot analysis to evaluate if the binding of the TAT-PSscan255 peptide to Ship2-Sam could avoid the inhibition of EphA2 endocytosis and degradation [67]. For this purpose, PC-3 cells were treated with ephrinA1-Fc (1 µg/mL for 2 h) and TAT-PSscan255 (50 µM for 4 h), both alone and in combination (i.e., pre-treating with TAT-peptide 50 µM for 2 h followed by the addition of ephrinA1-Fc 1 µg/mL for an extra 2 h) (Figure 7b).

EphrinA1-Fc is an engineered ligand in a dimeric form [68]. The stimulation of PC-3 with only the ephrinA1-Fc ligand causes a decrease in EphA2 levels upon the induction of receptor internalization and degradation (Figure 7b) [66]. Treatment with TAT-PSscan255 alone can also induce a certain decrease in EphA2 levels with respect to the untreated control (Figure 7b). Finally, the stimulation of PC-3 with TAT-PSscan255 in combination with ephrinA1-Fc causes a reduction in EphA2 levels like that observed for the ephrin ligand when used by itself (Figure 7b). These data indicate that the PSscan255 peptide can modulate EphA2 receptor endocytosis and degradation slightly and positively. Nevertheless, PSscan255, when used in combination with the natural ephrin ligand, is unable to enhance its action. Possibly, an increase in peptide binding affinity for Ship2-Sam is required to significantly alleviate the Ship2 inhibitory function towards receptor endocytosis.

## 3. Materials and Methods

### 3.1. FoldX Studies

The design of the KRI3 mutant peptides was achieved using the software FoldX (v. 5) (Centre for Genomic Regulation (CRG), Barcelona, Spain) through a three-step protocol based on a first “PositionScan” run, followed by a “BuildModel” cycle (Figure 2), and a final analysis by the “AnalyseComplex” macro [28,29]. The input structure for the FoldX approach consisted of the best docking model of the KRI3/Ship2-Sam complex, which we previously generated with Haddock [27,32] after the addition of non-polar hydrogens by UCSF Chimera (version 1.16) [69]. As the in silico protocol started from a Haddock optimized structure, no “RepairPDB” cycles were performed. The macros “PositionScan” and “BuildModel” were run by using default settings (i.e., number of runs: 1, pH 7, temperature 298 K, and ionic strength 0.05 M) [29]. The “PositionScan” macro was employed to evaluate the changes in stability occurring upon single-site mutagenesis within each position of the KRI3 peptide sequence [34,35]. The best mutations for each peptide position (i.e., 11 point mutations linked to 6 peptide positions (Table S1)) were selected based on the ΔΔG and ΔVdW (=difference in Van der Waals clashes, which indicates the steric overlaps between atoms and is connected to strong repulsive interactions [28]) criteria indicated in the Supplementary Table S1 and Figure 2, Box 2.

Then, a library of 348 KRI3 multiple mutant peptides (47 double, 101 triple, 116 four-fold, 68 five-fold, and 16 six-fold mutants) was built through the “BuildModel” macro of FoldX by considering all possible combinations of the most stabilizing point mutations identified through the first “PositionScan” cycle (Table S1). The stability of each member of the peptide library was further evaluated by the “BuildModel” macro [36,37,70]. In detail, combinations of mutations leading to the most stabilizing effects were established by analyzing the “Dif_PDB_BM.fxout” output file, which contains the energy differences between mutant and reference wild-type structures of the peptide/Ship2-Sam complexes, and considering the ΔΔG and ΔVdW thresholds indicated in the Supplementary Table S2 and in Figure 2, Box 4 [28,29,70]. This selection strategy led to the choice of 7 peptide sequences.

The binding affinities of these 7 mutant peptides towards Ship2-Sam were evaluated with the “AnalyseComplex” macro [28,29]. In details, the difference between interaction energies in the structure of Ship2-Sam/mutant peptide complexes with respect to their reference wild-type structures (=ΔΔGbind), generated by the “BuildModel” macro in the previous step, were computed. This last analysis was also based on the evaluation of “IntraclashesGroup1” (expressed in Table S3 as ΔVdW_Ship2-Sam_) and “IntraclashesGroup2” (expressed in Table S3 as ΔVdW_Peptide_). “Interaction Energy” values and “Intraclashes” were retrieved from the “Summary_AC.fxout” output files of the AnalyseComplex macro [28,29]. In the end, considering interaction energies, penalties due to Van der Waals clashes, chemical similarity, and synthetic chemistry feasibility, 3/7 mutant KRI3 peptides were selected and experimentally evaluated (Table S3 and Figure 2).

### 3.2. Peptides

The PSscan255, PSscan258, and PSscan266 peptides were purchased from GenScript (Rijswijk, The Netherlands). Peptide purities were at least 95% (Table S4 and Figures S24–S35). Three lyophilization cycles of peptide samples were performed to remove as much TFA (trifluoroacetic acid) as possible. Prior to peptide use in cell-based assays, full TFA removal and its exchange with acetate were carried out.

### 3.3. CD Spectroscopy

CD investigations were conducted as reported before [26,32]. Each PSscan peptide was analyzed at a 100 μM concentration in a 10 mM phosphate buffer at pH 7.4, with and without TFE (from 0 to 80%), by employing a quartz cuvette (0.1 cm path length). CD spectra were registered on a Jasco J-810 spectropolarimeter (JASCO Corp, Milan, Italy), over the wavelength range from 190 to 260 nm, at 25 °C. CD spectra, reported in mean residue ellipticity (deg × cm^2^ × dmol^−1^ × res^−1^), are the average of three scans after the subtraction of the associated blanks. The BESTSEL software (http://bestsel.elte.hu/ accessed on 10 April 2024) (Department of Biochemistry Institute of Biology Eötvös Loránd University, Budapest, Hungary) was used for the deconvolution of CD spectra [71].

### 3.4. Protein Expression

Ship2-Sam and EphA2-Sam were expressed in the ^15^N or unlabeled forms as recombinant proteins in *E-coli*, as previously reported [8,72].

### 3.5. NMR Spectroscopy

NMR experiments were recorded at 298 K on a Bruker Avance 500 MHz spectrometer equipped with a cryoprobe (Bruker Italia Srl, Milan, Italy), with samples consisting of 600 μL total volumes.

#### 3.5.1. Conformational Studies

For the conformational analyses, peptide samples were prepared in two different solvent conditions: PBS (phosphate-buffered saline—10 mM phosphates, 137 mM NaCl, and 2.7 mM KCl—obtained from Sigma-Aldrich by Merck Group, Milan, Italy) with a pH of 7.4 with 10% (*v*/*v*) D_2_O (98% D, Sigma-Aldrich by Merck Group, Milan, Italy), and a mixture of 2,2,2-trifluoroethanol-d3 (TFE, 99.5% isotopic purity, Sigma-Aldrich by Merck Group, Milan, Italy)/PBS (50/50—*v*/*v*). We performed 2D [^1^H, ^1^H] TOCSY (Total Correlation Spectroscopy) [73], NOESY (Nuclear Overhauser Enhancement Spectroscopy) [39], ROESY (Rotating-Frame Overhauser Enhancement Spectroscopy) [40], and DQFCOSY (Double Quantum-Filtered Correlated Spectroscopy) [74] experiments for each peptide, as previously described [32]. The suppression of residual water signal was achieved through excitation sculpting [75]. Chemical shifts were referenced to the water signal (4.75 ppm) in experiments, performed in PBS/D_2_O, and to internal TSP (Trimethylsilyl-3-propionic acid sodium salt-D4, 99% D, from Sigma-Aldrich by Merck Group, Milan, Italy) (0.0 ppm) in experiments performed in TFE/PBS. Spectra processing and analyses were performed with the software TopSpin 4.2 (Bruker, Milan, Italy) and NEASY [76], the latter being incorporated into CARA (http://cara.nmr.ch/doku.php/). Proton resonance assignments were obtained for PSscan255, PSscan266, and PSscan258 peptides with a standard approach relying on the comparison of TOCSY and NOESY spectra [41]. Chemical shift deviations from random coil values for Hα protons (CSD = Δδ_(Hα)_ = δ_(Hα)_observed − δ_(Hα)_random-coil) were calculated, following the approach suggested by Kjaergaard et al. [77], to evaluate random coil chemical shifts (https://www1.bio.ku.dk/english/research/bms/sbinlab/randomchemicalshifts1, accessed on 18 January 2024).

#### 3.5.2. Chemical Shift Perturbation Studies

[^1^H-^15^N] HSQC spectra for chemical shift perturbation studies were acquired with ^15^N labeled Ship2-Sam (20 μM concentration) in the absence and presence of PSscan255, PSscan258, and PSscan266 peptides (200 μM concentrations). For these experiments, concentrated peptide stocks in PBS were diluted in protein samples. The pH was checked upon each peptide’s addition and eventually carefully adjusted with NaOH or HCl solutions to avoid pH variations in the NMR samples. Chemical shift perturbation studies with 1D [^1^H] NMR experiments were similarly recorded for Ship2-Sam alone (27 μM concentration) and in the presence of PSscan peptides (273 μM concentration). Spectra under identical experimental conditions were recorded for Ship2-Sam alone and following the addition of the control peptides.

[^1^H-^15^N] HSQC spectra of ^15^N labeled EphA2-Sam (33 μM), without and with the PSscan255 peptide (300 μM), were also acquired.

#### 3.5.3. Solution Structure Calculations

The NMR solution structures of PSscan255, PSscan258, and PSscan266 peptides in TFE/PBS (50/50—*v*/*v*) were calculated using CYANA 2.1 [44] and a well-established protocol [32]. Briefly, the 2D [^1^H, ^1^H] NOESY spectrum (300 ms mixing time) was employed to derive distance constraints, while angular constraints were created by the GRIDSEARCH module of CYANA [44]. Structure calculations started from 100 random conformers; the 20 structures with the lowest CYANA target functions (=the highest agreement with experimental restraints) were validated with the programs MOLMOL 2K.2 [45] and PROCHECK-NMR (v. 3.4) [78].

### 3.6. BioLayer Interferometry

For BLI interaction assays, the Sartorius Octet^®^ N1 System and streptavidin (SA)-coated biosensor tips (Sartorius Italy S.r.l., Grassina-Bagno a Ripoli (FI), Italy) were employed. SA biosensor tips were first hydrated in 1X PBS buffer; then, to achieve ligand immobilization, the tips were dipped, for 300 sec with 1000 rpm agitation, into 4 µL of biotinylated–pegylated PSscan255 solution (50 µg/mL). Following peptide loading, each tip was placed in contact, for 300 s with a 1000 rpm agitation speed, with 4 µL of Ship2-Sam protein at several concentrations (20, 50, 100, 300, and 500 µM). All mathematical manipulations were handled by the Octet Analysis Studio 12.2 software from Sartorius. A 1:1 Langmuir binding model was used for data fitting, and the K_D_s were obtained through the global analysis mode. 

### 3.7. Docking Studies: Ship2-Sam/PSscan255 Complex

Docking studies were performed with the Haddock 2.4 webserver (https://rascar.science.uu.nl/haddock2.4/ accessed on 15 June 2024) [27,50], starting from the NMR structure of Ship2-Sam (1st conformers of the NMR ensemble, PDB code 2K4P [8]) and that of the PSscan255 peptide (1st conformers of the NMR ensemble). The protonation states of H47 and H74 from the input Ship2-Sam structure (PDB code 2K4P [8]) were retained by selecting “HISD” in the “Input parameters” section of Haddock 2.4 webserver [50].

Docking runs without experimental restraints were conducted using the optimal run settings suggested on the Haddock submission page [79]. All protein residues with a solvent exposure, which was evaluated with the software MOLMOL 2K.2 [45], higher than 40% (i.e., A27, R34, R40, E43, H47, E54, F55, D58, E62, E66, Q70, P72, R76, Q83, L84, and K86) were considered to be active [80]. The number of MD (molecular dynamics) steps for rigid-body high-temperature TAD (Torsion Angle Dynamics), those during the first rigid-body cooling stage, the second cooling stage with flexible side chains at the interface, and the third cooling stage with a fully flexible interface were set to 2000, 2000, 4000 and 4000, respectively. In addition, the number of partitions for random exclusion was set to 1.1428 and the number of trials for rigid-body minimization was set to 1, whereas the number of structures for rigid-body docking, for semi-flexible refinement, and for the final refinement, as well as the number of structures to analyze, were set to 10000, 400, 400, and 400, respectively [79].

The best 10 solutions in terms of Haddock scores were visually inspected, and 5 Ship2-Sam/PSscan255 structures representative of diverse binding modes were more deeply analyzed regarding intermolecular contacts (See Supplementary Materials).

### 3.8. AlphaFold Model of the Ship2-Sam/PSscan255 Complex

To predict a model of the Ship2-Sam/PSscan255 complex, AlphaFold2 [18,20] was run through the ColabFold (v1.5.5) server [81] (https://colab.research.google.com/github/sokrypton/ColabFold/blob/main/AlphaFold2.ipynb#scrollTo=kOblAo-xetgx, accessed on 10 September 2023), as recently reported [25]. The Ship2-Sam sequence (residues from 1194 to 1258 of human Ship2 (UniprotKB [82] entry O15357)) and the PSscan255 peptide sequences were used as inputs. Five models were predicted and subjected to post-prediction relaxation via gradient descent in the Amber force field [81,83].

### 3.9. Docking Studies: PSscan266 Self-Aggregation

Models of PSscan266 in a dimeric arrangement were obtained with Haddock (Haddock 2.4 webserver (https://rascar.science.uu.nl/haddock2.4/ accessed on 19 June 2024) [50], using as both ligand and receptor either the NMR structure of PSscan266 (1st NMR conformer), calculated in a solution containing TFE that possesses a helical structuration, or by employing an extended conformation of the peptide. The PSscan266 peptide in an extended conformation was built in UCSF Chimera (version 1.16) (UCSF Resource for Biocomputing, Visualization, and Informatics, San Francisco, CA, USA) [69] by imposing backbone dihedral angles, canonical of an extended structural organization (*ϕ* = −139°, *ψ =* 135°). During docking calculations, the peptide N- and C-termini were considered acetylated and amidated, respectively, and the aromatic residues (Trp, Phe, and Tyr) were set as active. The docking protocol first included a rigid-body energy minimization that provided 1000 output structures. In the following step, the best 200 solutions underwent a semi-flexible simulated annealing; next, a final refinement in water was carried out. The best 10 structures in terms of Haddock scores were visually inspected with UCSF Chimera [69], and the intermolecular interactions were further analyzed with LigPlot+ [51,52].

### 3.10. LigPlot+ Analyses

Two-dimensional diagrams of intermolecular interactions in representative Haddock models of Ship2-Sam/PSscan255 complexes and PSscan266 dimers were generated with LigPlot+ (version 2.2.8) [52]. H-bonds were identified using the following thresholds: 2.7 Å (H–acceptor distance) and 3.35 Å (donor–acceptor distance). Regarding non-bonded protein–peptide interactions, these were selected by choosing cut-off values equal to 2.9 Å and 3.9 Å for the minimum and maximum distances between any atoms in any residues, respectively [51,52]. Salt bridges between negatively and positively charged residues were identified following the criteria indicated in reference [84].

### 3.11. Serum Stability

The PSscan255 peptide was mixed with 25% fetal bovine serum (FBS, from Merck group, Milan, Italy), previously incubated at 37 °C for 15 min, to obtain a peptide concentration equal to 80 µM in the end. These mixtures were kept at 37 °C and, at several time intervals (0, 1, 2, 3, 4, 5, 17, and 19 h), aliquots of the incubating solutions (50 µL) were quenched by using 15% trifluoroacetic acid (TFA) (50 µL) and then incubated at 2 °C for 15 min. Next, serum proteins were discarded by centrifugation (15 min at 3000 rpm), and samples were subjected to reverse-phase high-performance liquid chromatography (RP-HPLC). RP-HPLC runs were conducted with an HPLC LC-4000 series (Jasco) equipped with a UV detector by using a C18-Kinetek column (Phenomenex, Milan, Italy). Gradient elution was conducted at 25 °C; the gradient started with buffer A, consisting of water with 0.1% TFA. Then buffer B, consisting of acetonitrile with 0.1% TFA, was added from 5 to 70% in 20 min. The peak areas were integrated at diverse time lapses. Two assays were performed, and data points represent average values over the two experiments.

### 3.12. Cell Culture Conditions and Cytotoxicity

Media and supplements were obtained from Thermo Fisher Scientific (Milan, Italy). PC-3 cells were cultured in the RPMI 1640 medium enriched with 10% fetal bovine serum (FBS) and 2 mM L-glutamine. NHDFs were cultured in the DMEM medium complemented with 2% FBS, 1 μg/mL hydrocortisone, 10 ng/mL human epidermal growth factor, 3 ng/mL basic fibroblast growth factor, and 10 μg/mL heparin. For the cytotoxicity assay 96-well flat-bottom microplates were used and 4 × 10^3^ cancer cells and 5 × 10^3^ NHDF cells were seeded in 50 μL of medium per well and incubated overnight to permit cell adhesion at 37 °C in a 5% CO_2_ humidified atmosphere. Following the removal of the culture medium, cells were incubated with 100 μL of their growth medium containing 50 and 100 μM TAT-PSscan255 for 4 h. TAT-PSscan255 was dissolved in water. The crystal violet assay was performed to assess cell viability. The amount of dye taken up was quantified with a plate reader (Multiskan Fc 10094, Thermo Fisher Scientific, Milan, Italy) at 595 nm. Data, reported as means ± SEM of three independent experiments carried out in quadruplicate, were presented as the percentage of proliferating cells compared to the control (vehicle-treated cells).

### 3.13. Western Blotting Analyses

PC-3 cells were treated with TAT-PSscan255 (50 μM) for 4 h and ephrinA1-Fc (1 μg/mL) for 2 h, both alone and in combination. After treatments, ice-cold PBS was used to wash cells three times and then cells were collected at 4 °C from a RIPA lysis buffer (Merck Group/Sigma-Aldrich, Milan, Italy) enriched with complete protease and phosphatase inhibitors (Roche, Monza, Italy). Lysates were centrifuged at 13,000 rpm for 30 min at 4 °C and the protein concentration in the supernatants was evaluated using the Bradford assay. Western blotting analysis was performed by first running protein aliquots (30 μg/lane) on SDS-PAGE (4–12%, Life Technology, Milan, Italy) and then blotting them onto nitrocellulose membranes (Novex, Life Technology, Milan, Italy). Membranes were blocked with 5% non-fat milk for 1 h at room temperature and next incubated overnight at 4 °C with EphA2 (#SC-924) and β-actin (#47778) (Santa Cruz Biotechnology, Dallas, TX, USA). The membranes were then incubated for 1 h at room temperature with the proper goat anti-rabbit or goat anti-mouse secondary HRP-coupled antibodies. To visualize immunocomplexes, enhanced chemiluminescence and autoradiography were employed according to the manufacturer’s protocol (Santa Cruz Biotechnology, Dallas, TX, USA). Quantification was achieved through densitometric analysis with the ImageJ software (v.1.54g) (National Institutes of Health, Bethesda, MD, USA, https://imagej.org). Statistical significance regarding the comparison between treated and untreated cells was evaluated with one-way ANOVA using Dunnett’s post-test analysis. Statistical analysis was assessed with the OriginPro software v.2023 (OriginLab Corporation, Northampton, MA, USA).

### 3.14. Fluorescence Microscopy

The FITC-TAT-PSscan255 peptide (50 μM) was added for 4 h to PC-3 cells (5 × 10^4^/mL) that were positioned on coverslips inside a Petri dish. Following incubation, cells were first washed three times with PBS and then fixed in 4% paraformaldehyde in PBS for 15 min. Next, cell permeabilization was achieved via treatment with 0.3% Triton-X 100 in PBS for 30 min. After blocking for 15 min with 2% bovine serum albumin, the coverslips were marked with phalloidin-iFluor 594 (Abcam, Milan, Italy), which was diluted 1:1000 for 1 h. After three extra rinses with PBS, the nuclei were stained with Hoechst 33342 (Thermo Fisher Scientific, Milan, Italy), diluted 1:2000, and incubated for 15 min in the dark. Lastly, the coverslips were mounted onto microscope slides with Mowiol (Sigma from Merck Group, Milan, Italy) and permitted to cure for at least 48 h before undergoing microscopy imaging. Images were taken with a Nikon Eclipse Ti2-E inverted research microscope (Nikon Instruments, Melville, NY, USA) using standard phase contrast, epifluorescence microscopy, and deconvolution. Image acquisition was accomplished with an Andor Zyla 4.2+ sCMOS high-sensitivity monochrome camera and managed by Nikon NIS-Elements Advanced Research (AR) image acquisition and analysis software.

## 4. Conclusions

Peptide antagonists of the heterotypic Sam–Sam association between the EphA2 receptor and the Ship2 lipid phosphatase may work as anticancer agents by releasing the Ship2 inhibitory function of receptor endocytosis and degradation [11]. KRI3 is a positively charged peptide able to interact with Ship2-Sam by targeting the EphA2-Sam binding site (i.e., the ML interface) [26]. Very recently, to improve the binding affinity and specificity of KRI3 towards Ship2-Sam, we developed a computation approach that made use of the Haddock Refinement Interface [25,27]. Herein, similarly starting from a docking model, which we previously built for KRI3 in complex with Ship2-Sam, we set up a different in silico approach to design optimized Ship2-Sam peptide ligands. The novel computational strategy relies on the software FoldX [29] and consists of three main steps. First, the macro “PositionScan” was used to establish in KRI3 the most stabilizing mutations (in terms of folding energy), which should be inserted in each peptide position. Then, the macro “BuildModel” was used to gather multiple mutations together and obtain a list of mutant peptides with 2–6 amino acid substitutions and improved stabilities (in terms of folding energies) with respect to the KRI3 starting peptide. In the last step, the interaction affinities for Ship2-Sam in selected peptides were evaluated using the “AnalyseComplex” macro [85]. The results of the computational study pointed out that the most advantageous mutations should be inserted in the first and second N-terminal “KRIAY” motives, while it is better to leave the last C-terminal “KRIAY” stretch unmutated (Table S3). This novel information will be more deeply explored by analyzing a larger set of peptides.

The peptide with the largest predicted gain in binding affinity (i.e., PSscan255) contained increased aromatic content with respect to the KRI3 peptide and reduced positive charges and was selected for experimental protocol validation, along with two additional analog peptides with predicted non-optimal interaction affinities (i.e., PSscan258 and PSscan266).

NMR interaction studies confirmed in silico data and indicated better binding to Ship2-Sam for PSscan255 with respect to the other two peptides. The dissociation constant (K_D_) for the binding of PSscan255 to Ship2-Sam was estimated by BLI and was ~70 µM, thus revealing only a marginal enhancement of binding to Ship2-Sam with respect to the KRI3 starting peptide (K_D_~100 µM in SPR studies). Interestingly, PSscan255 had improved serum stability with respect to KRI3 [26,32]. Cell-based assays revealed some specific cytotoxic effects of the peptide towards prostate cancer cells (PC-3) with respect to healthy cells (NHDFs) and a weak ability to modulate EphA2 receptor endocytosis/degradation.

Considering that we tested only a very small set of peptides and that the best hit demonstrated some binding to the protein and a certain effect on EphA2 endocytosis in experimental studies, which was impressive, we believe that we have presented here a solid and potentially successful computational approach with which to optimize ligands of any target protein.

We should point out that the proposed FoldX-relying protocol can surely be further improved. For example, one can envision performing a larger optimization of the KRI3/Ship2-Sam complex starting geometry through several “RepairPDB” cycles [29]. Nevertheless, the energy selection criteria within the protocol can also be changed to collect a greater number of peptides for analysis in the last steps and improve the chance of picking a high-affinity ligand. However, we can also envision in the future applying some different in silico strategies for peptide design, relying either on MD simulation methods [15] or artificial intelligence [16] to optimize the Sam-targeting pioneering compounds identified in our laboratory.

In the end, the implemented computational approach coupled with the interdisciplinary experimental validation led to the identification of a novel Ship2-Sam ligand and the acquisition of detailed structural information on its mode of binding to the protein, which can support the development of novel anticancer compound inhibitors of the EphA2-Sam/Ship2-Sam association.

## Data Availability

Additional data are contained in the Supplementary Materials or will be provided by the corresponding author upon reasonable request.

## Supplementary Materials

Supporting Figures and Tables can be downloaded at: https://www.mdpi.com/article/10.3390/ijms251910616/s1, reference [86] is cited in Supplementary Materials.
