# Supplementary material for: Exploring a Potential Optimization Route for Peptide Ligands of the Sam Domain from the Lipid Phosphatase Ship2
Source: Int J Mol Sci. 2024 Oct 2;25(19):10616. doi: 10.3390/ijms251910616 (PMC11476629; doi:10.3390/ijms251910616)
Supplement: Supplementary file 1 [file ijms-25-10616-s001.zip › ijms-3210003-supplementary.pdf]

Supporting information:

ARTICLE

Exploring a Potential Optimization Route for Peptide Ligands of the Sam Domain from the Lipid Phosphatase Ship2

Marian Vincenzi [1], Flavia Anna Mercurio [1], Sara La Manna [2], Rosanna Palumbo [1], Luciano Pirone [1], Daniela Marasco [1,2], Emilia Maria Pedone [1], and Marilisa Leone [1,*]

1 Institute of Biostructures and Bioimaging, Via Pietro Castellino 111, 80131, Naples, Italy
2 Department of Pharmacy, University of Naples "Federico II", Via Domenico Montesano 49, 80131, Naples, Italy
* Correspondence: marilisa.leone@cnr.it

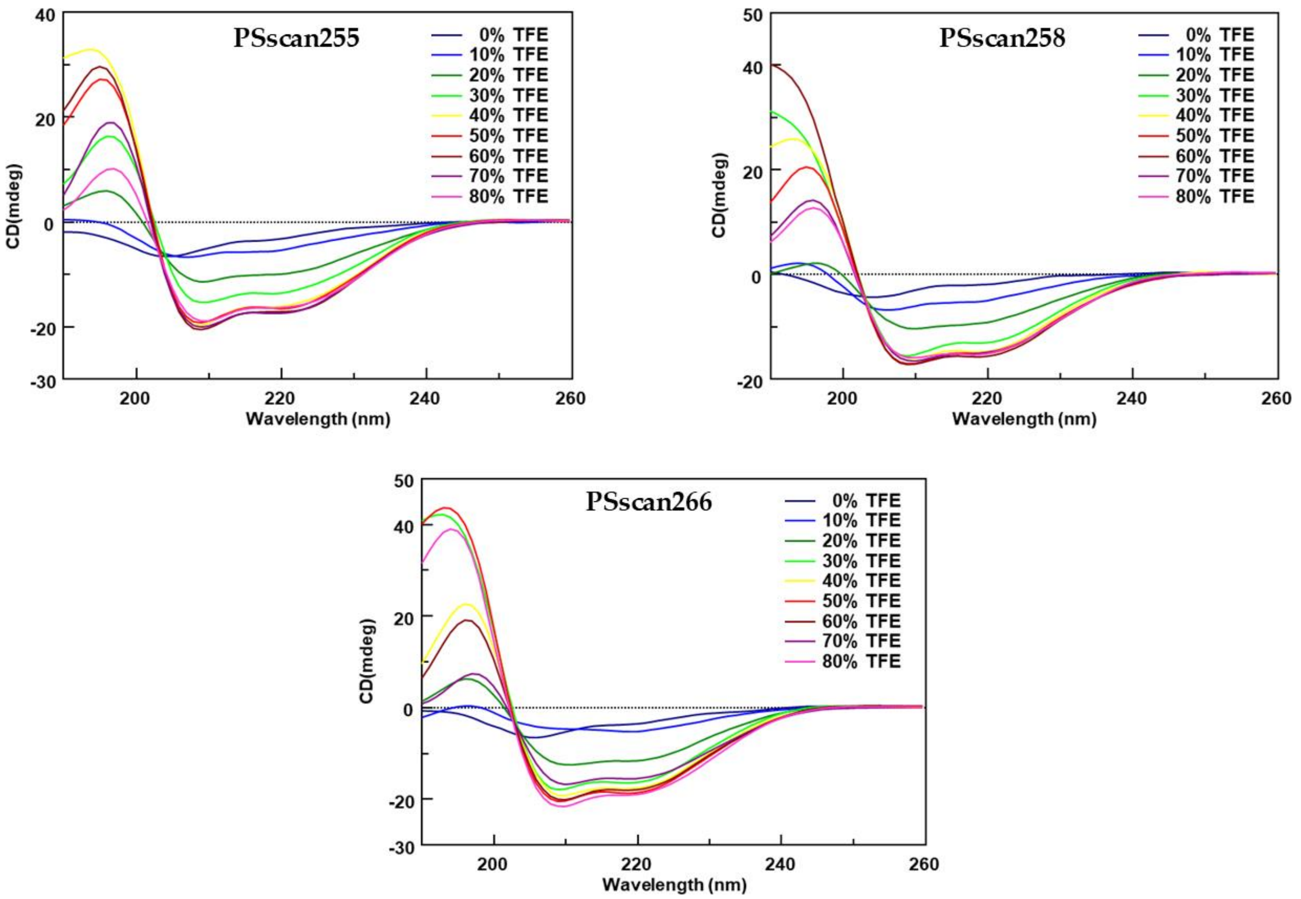

Figure S1. Overlay of CD spectra of the PSscan255, PSscan258, and PSscan266 peptides in 10 mM sodium phosphate buffer and at different TFE concentrations.

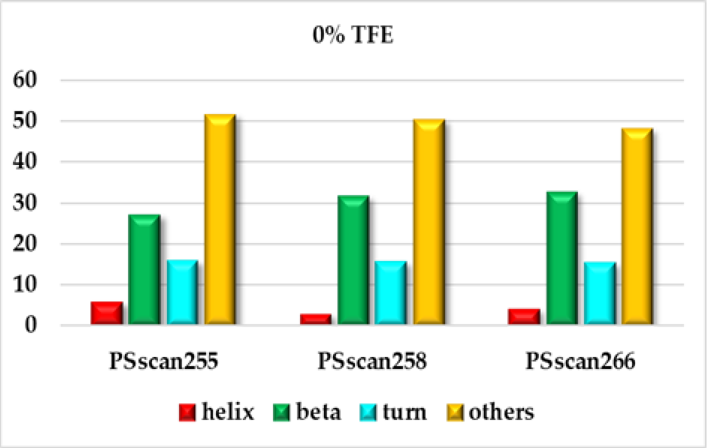

(a)

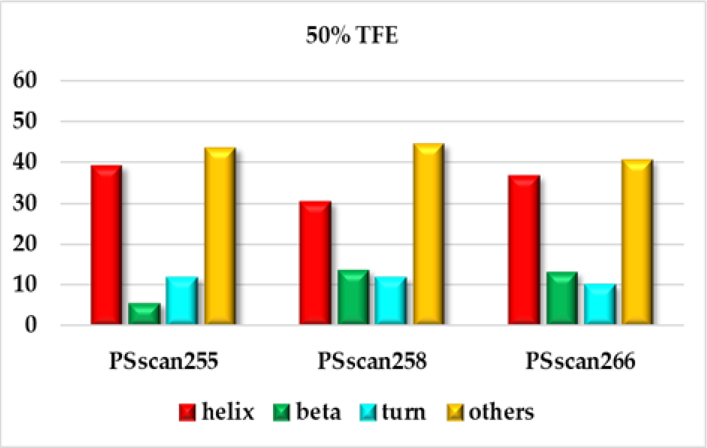

(b)

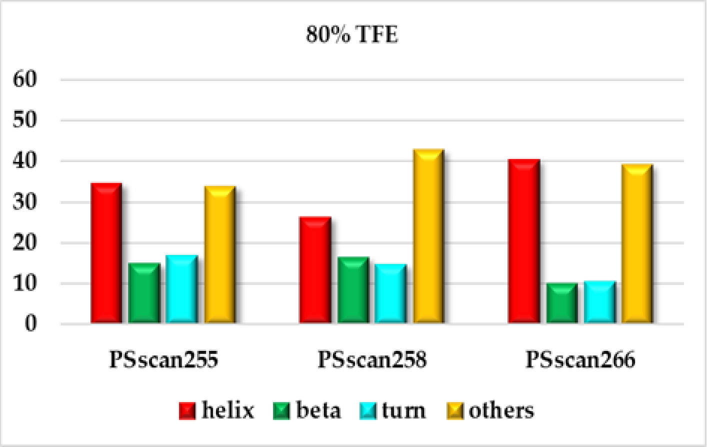

(c)

Figure S2. Deconvolution of CD spectra [1] recorded (**a**) without TFE; (**b**) with 50% and (**c**) 80% TFE.

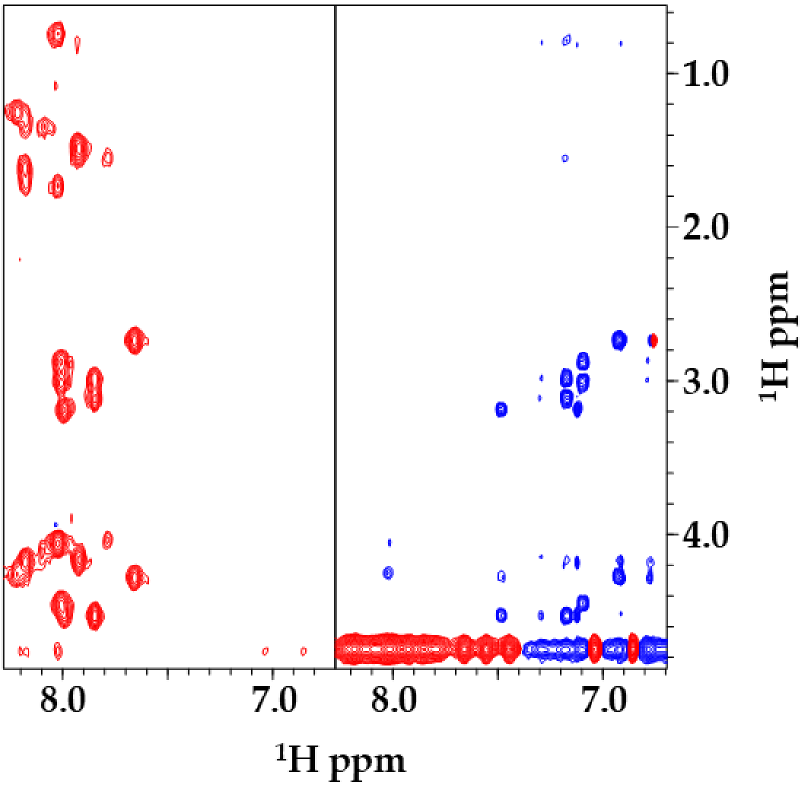

(a)

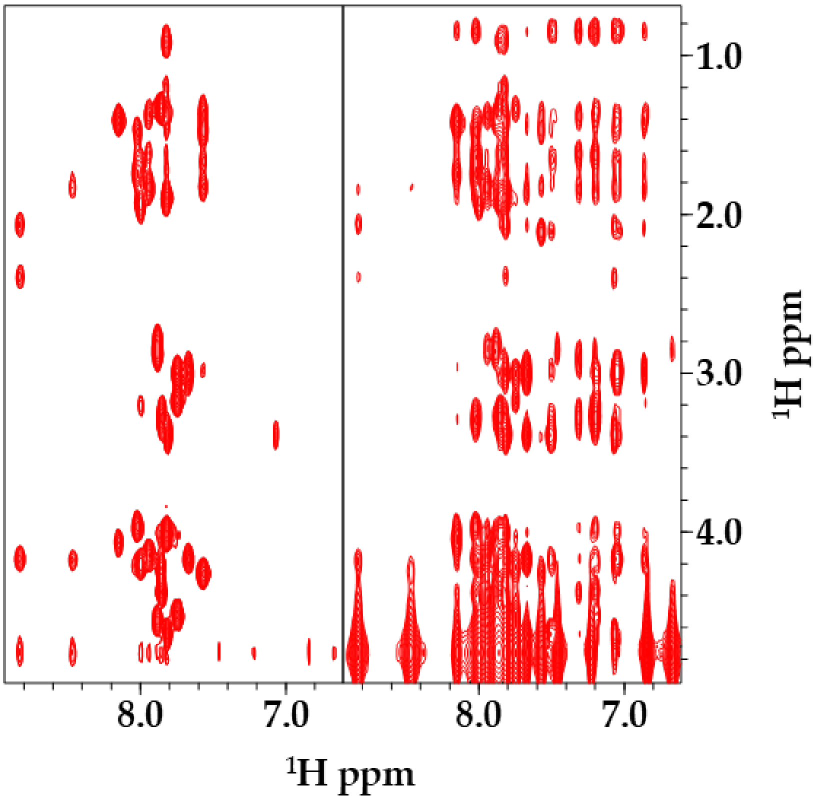

(b)

Figure S3. (**a, b**) NMR characterization of the PSscan255 peptide. (**a**) Comparison of 2D [^{1}H,^{1}H] TOCSY (left) and ROESY (right) spectra in PBS (300 µM peptide concentration, pH 7.4), and (**b**) of 2D [^{1}H,^{1}H] TOCSY (left) and NOESY (right) spectra in PBS/TFE (50/50 - v/v) (600 µM peptide concentration, pH 7.03). Spectral regions containing correlations between H_N and aromatic protons with aliphatic ones are shown.

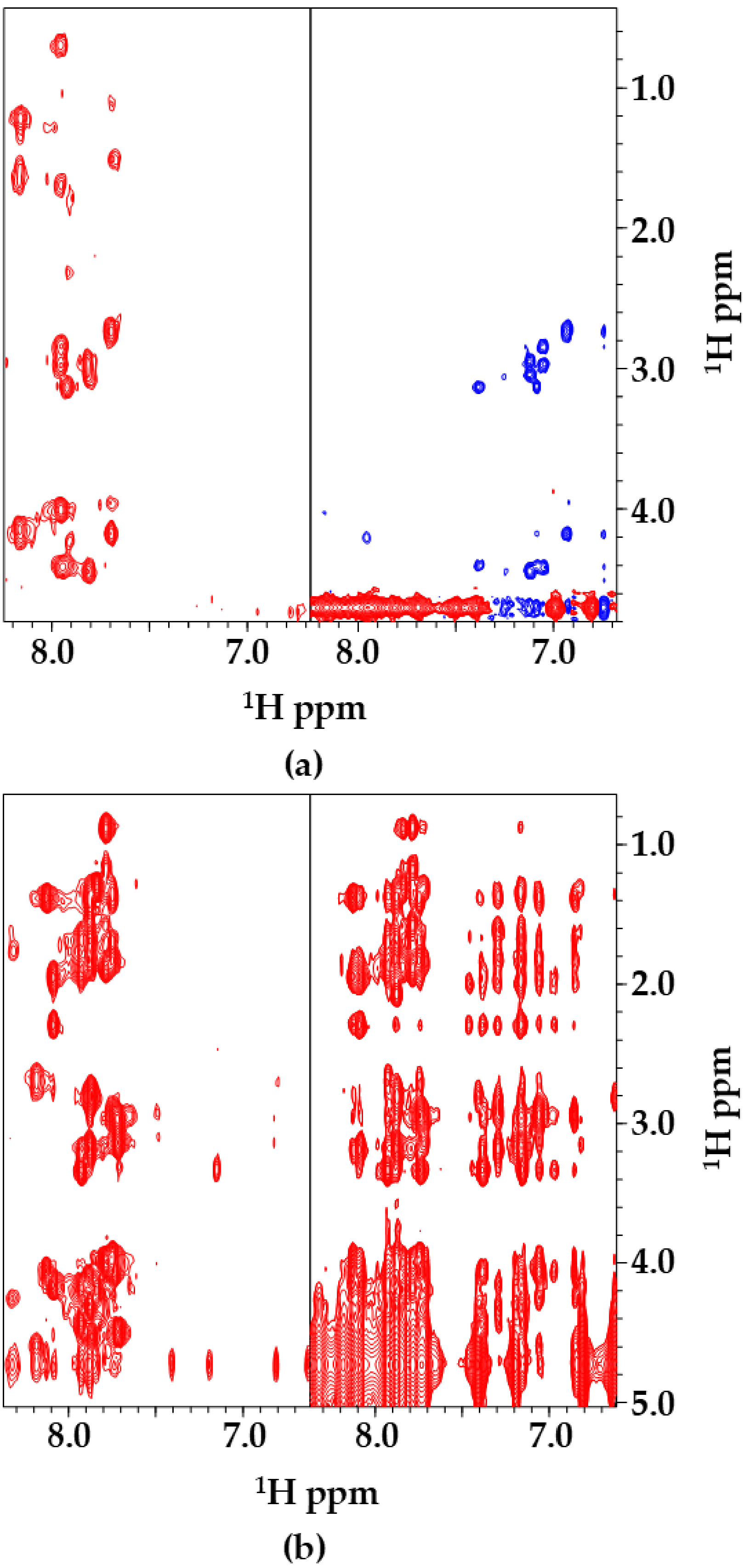

Figure S4. (**a**, **b**) NMR characterization of the PSscan258 peptide. Comparison of (**a**) 2D [^{1}H,^{1}H] TOCSY (left) and ROESY (right) spectra in PBS (300 µM peptide concentration, pH 7.38), and (**b**) of 2D [^{1}H,^{1}H] TOCSY (left) and NOESY (right) spectra in PBS/TFE (50/50 - v/v) (500 µM peptide concentration, pH 7.03). Correlations between H_N / aromatic protons and the aliphatic ones are displayed in the reported expansions.

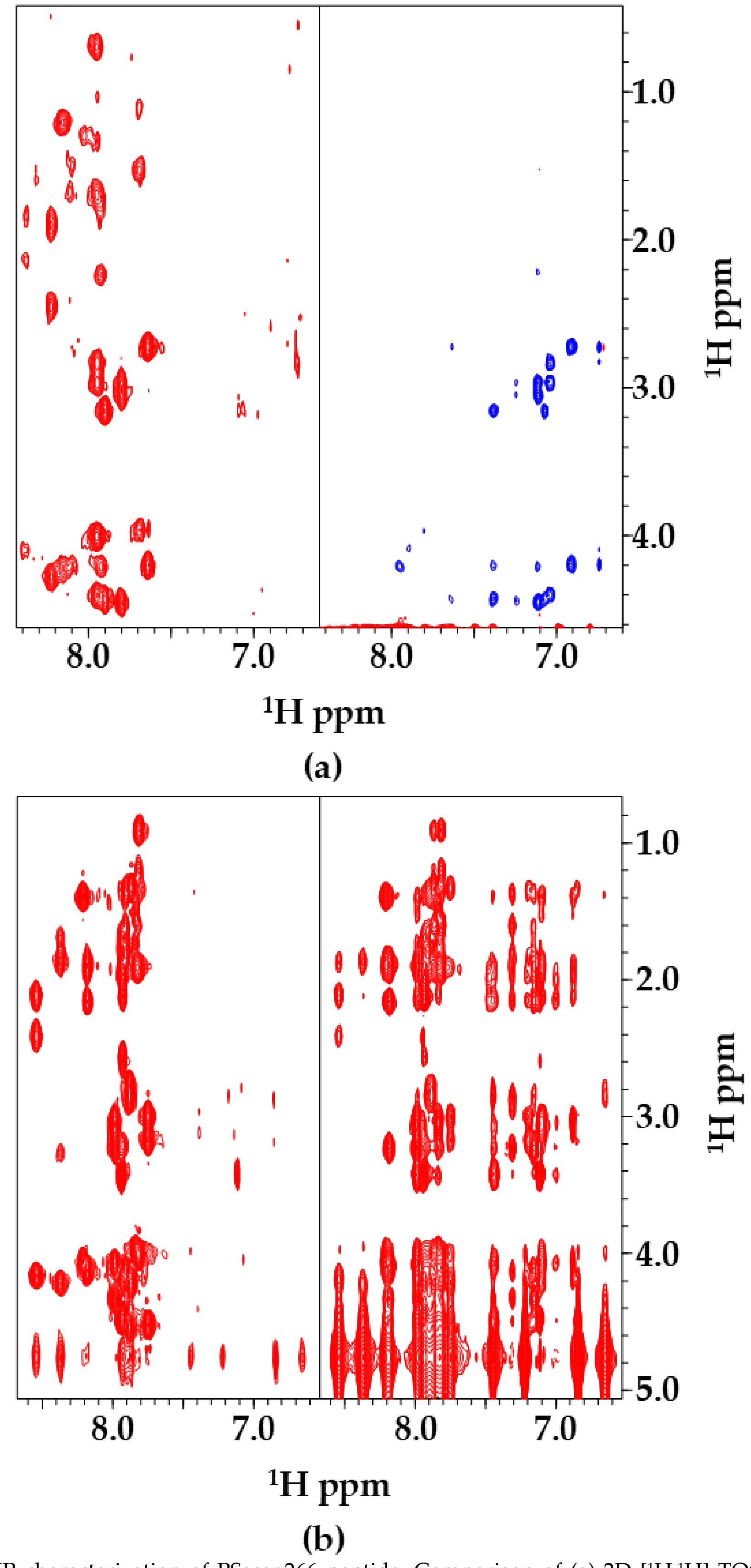

Figure S5. (**a**, **b**) NMR characterization of PSscan266 peptide. Comparison of (**a**) 2D [^{1}H,^{1}H] TOCSY (left) and ROESY (right) spectra in PBS (300 μM peptide concentration, pH 7.38), and (**b**) of 2D [^{1}H,^{1}H] TOCSY (left) and NOESY (right) spectra in PBS/TFE (50/50 - v/v) (492 μM peptide concentration, pH 7.09). Expansions of spectra that contain correlations between H_N and aromatic protons with aliphatic ones are shown.

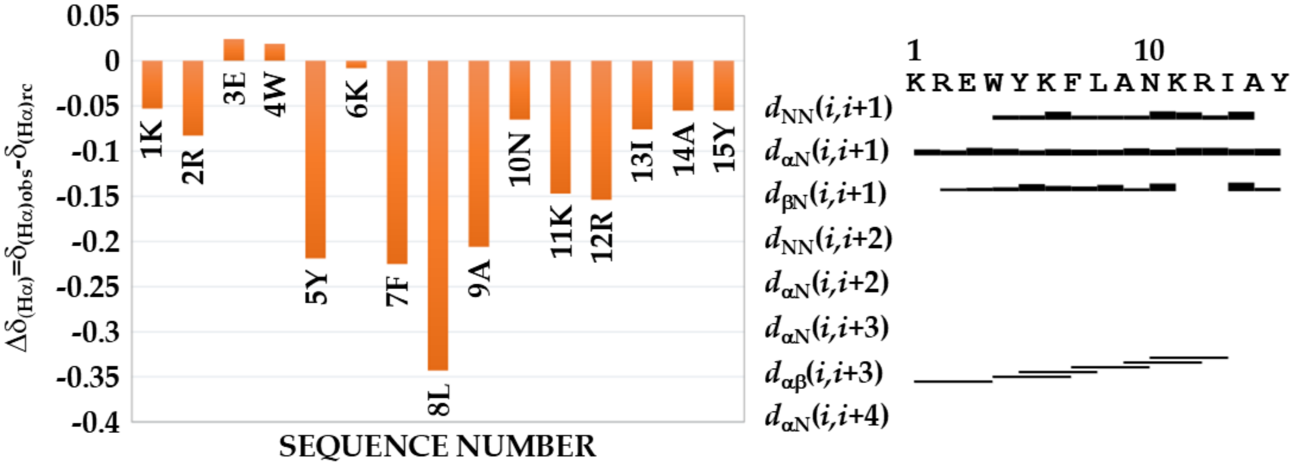

(a)

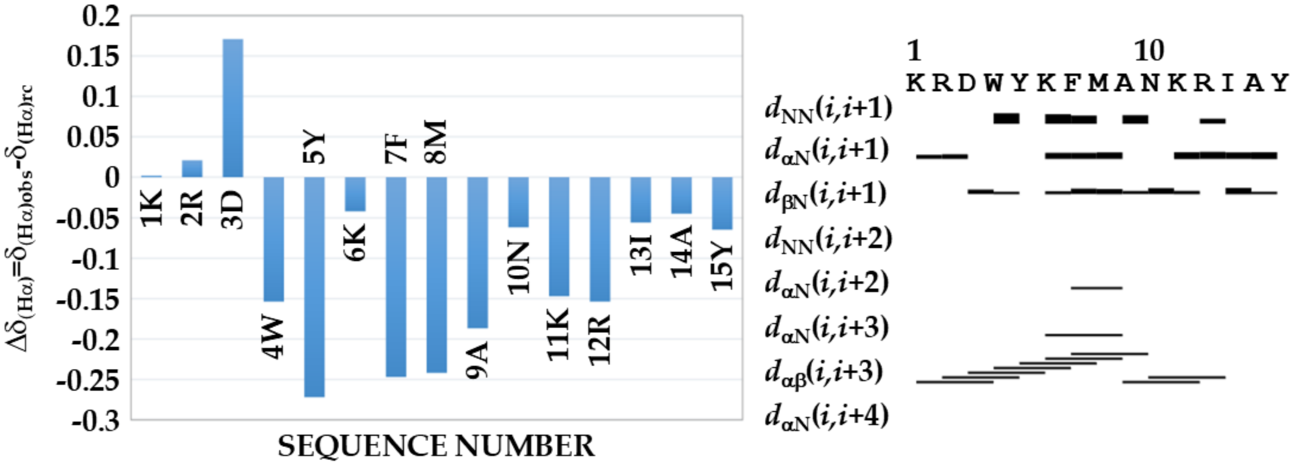

(b)

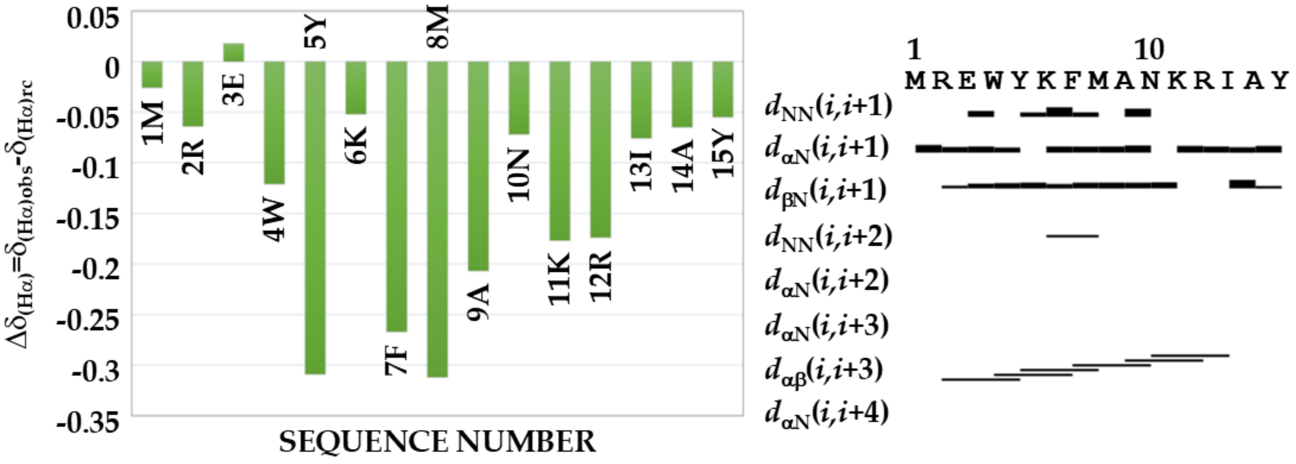

(c)

Figure S6. (**a, b, c** -Left panels) Comparison between H*α* chemical shifts ($\delta_{(H\alpha)obs}$) and the corresponding random coil values ($\delta_{(H\alpha)rc}$) (T=298K pH=7) for (**a**) PSscan255, (**b**) PSscan258, (**c**) PSscan266 [2]. (**a, b, c** -Right panels) Summary of significant NOEs, observed in solutions containing 50% TFE, for (**a**) PSscan255, (**b**) PSscan258, and (**c**) PSscan266; a NOE contact between the proton "a" in the residue "i" and the proton "b" in the residue "i + x", is reported as "d_{ab}(i, i +x)"; NOE intensities are proportional to the width of each bar.

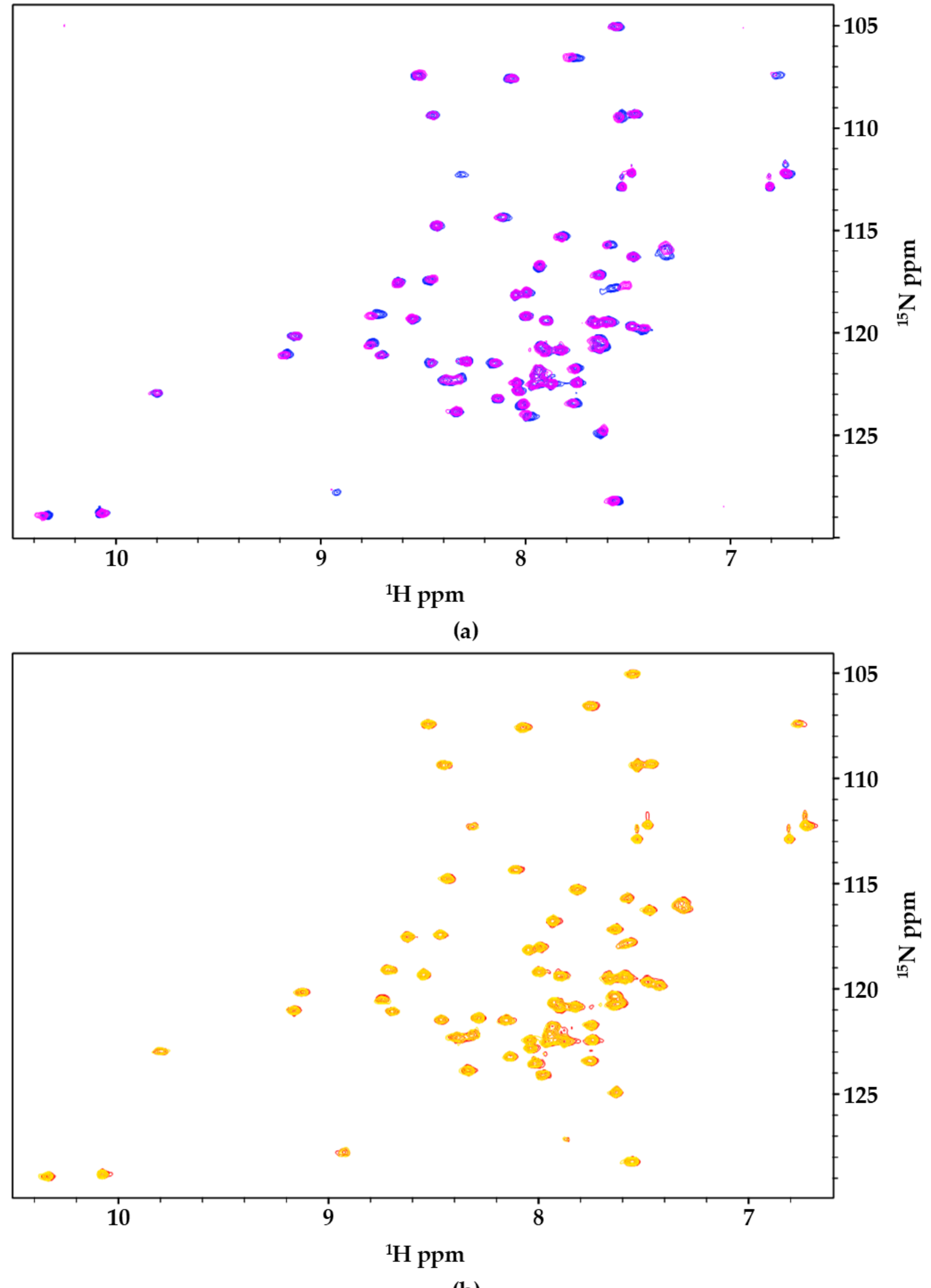

Figure S7. (**a**) Comparison of [^{1}H-^{15}N] HSQC spectra of Ship2-Sam (27 μM concentration) in its free form (blue) and after the addition of the KRI3 peptide (273 μM concentration) (magenta). (**b**) Comparison of [^{1}H-^{15}N] HSQC spectra of Ship2-Sam (27 μM concentration) in its free form (red) and after the addition of the CTRL peptide (273 μM concentration) (gold).

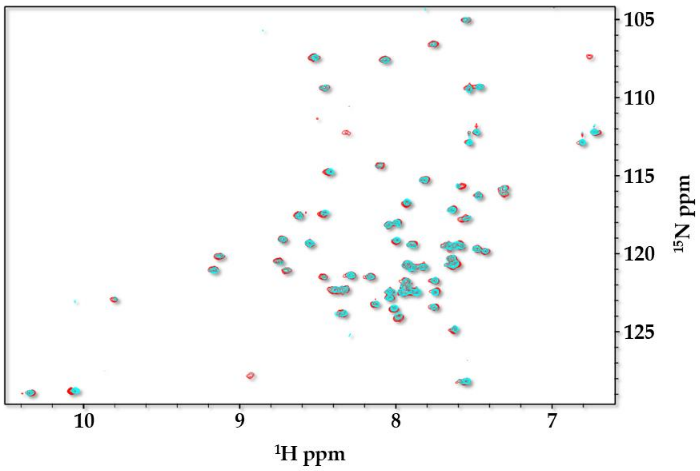

(a)

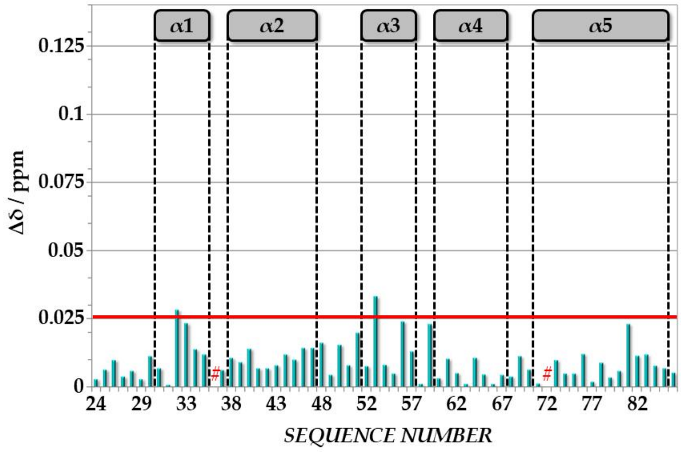

(b)

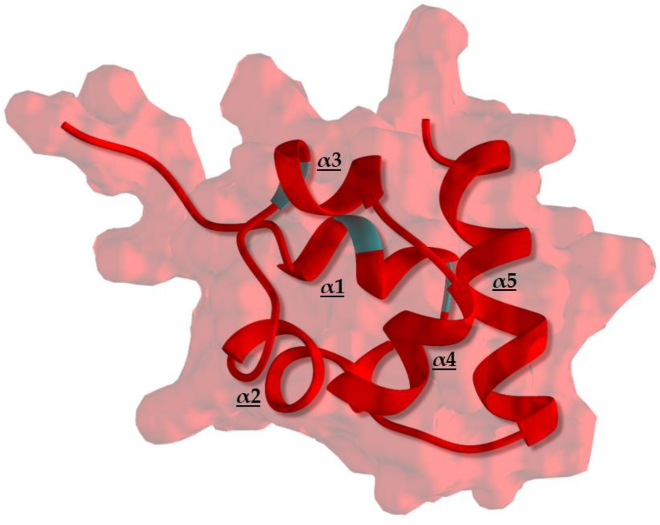

(c)

Figure S8. (**a**) The [^{1}H-^{15}N] HSQC spectrum of Ship2-Sam (20 μM concentration) in its free form (red) is superimposed to the one recorded after the addition of the PSscan258 peptide (200 μM concentration) (cyan). (**b**) Histogram showing chemical shift perturbations (CSP) (i.e., $\Delta\delta=[(\Delta H_N)^2 + (0.17 \times \Delta^{15}N_H)^2]^{1/2}$) [3] *versus* Ship2-Sam residue numbers. The "#" indicate residues with Δδ value set to zero (P72 and I36). (**c**) Protein residues that following peptide binding present $\Delta\delta \geq$ 0.025 ppm (i.e., W32 NHε1, L53) or disappear (i.e., I36), have been tinted in cyan on the 3D solution structure of Ship2-Sam (PDB entry code 2K4P [4]). Ship2-Sam is shown in a combined ribbon and translucent surface representation.

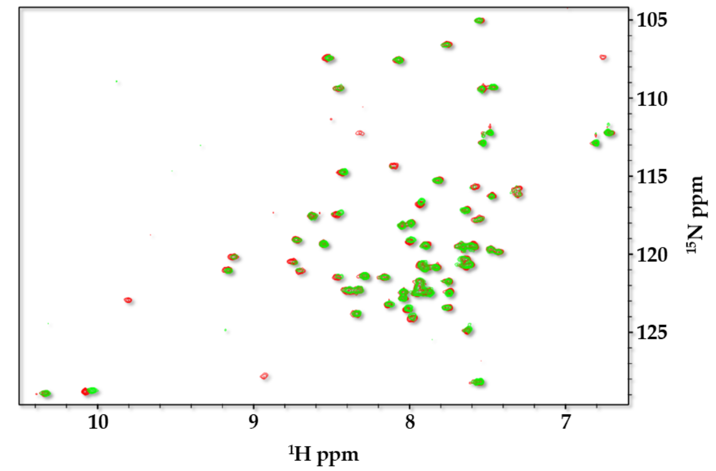

(a)

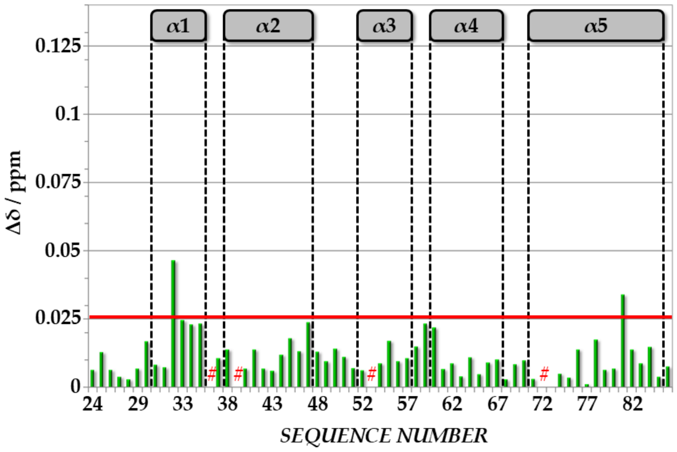

(b)

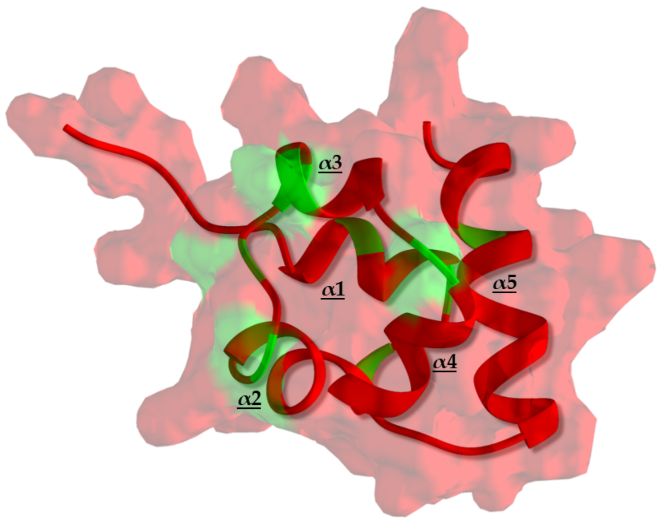

(c)

Figure S9. (**a**) The [^{1}H-^{15}N] HSQC spectrum of Ship2-Sam (20 μM concentration) in its unbound form (red) is overlayed with the corresponding spectrum recorded after the addition of the PSscan266 peptide (200 μM concentration) (light green). (**b**) Chemical shift perturbations (i.e., CSP=$\Delta\delta=[(\Delta H_N)^2 + (0.17 \times \Delta^{15}N_H)^2]^{1/2}$) [3] *versus* Ship2-Sam residue numbers. Δδ values are set equal to zero for P72, and also for I36, E39, L53 whose peaks disappear in the spectrum of the peptide/protein complex ("#"). (**c**) Residues with large CSP values (i.e., $\Delta\delta \geq 0.025$ ppm : W32 NHε1 and T81) and those decreasing in intensity or disappearing following peptide binding (i.e., I36, E39, N48, D51, L53, F55 and T60) have been dyed in light green on the NMR structure of Ship2-Sam (PDB entry code 2K4P [4]) that is shown in the mixed ribbon / transparent surface representation.

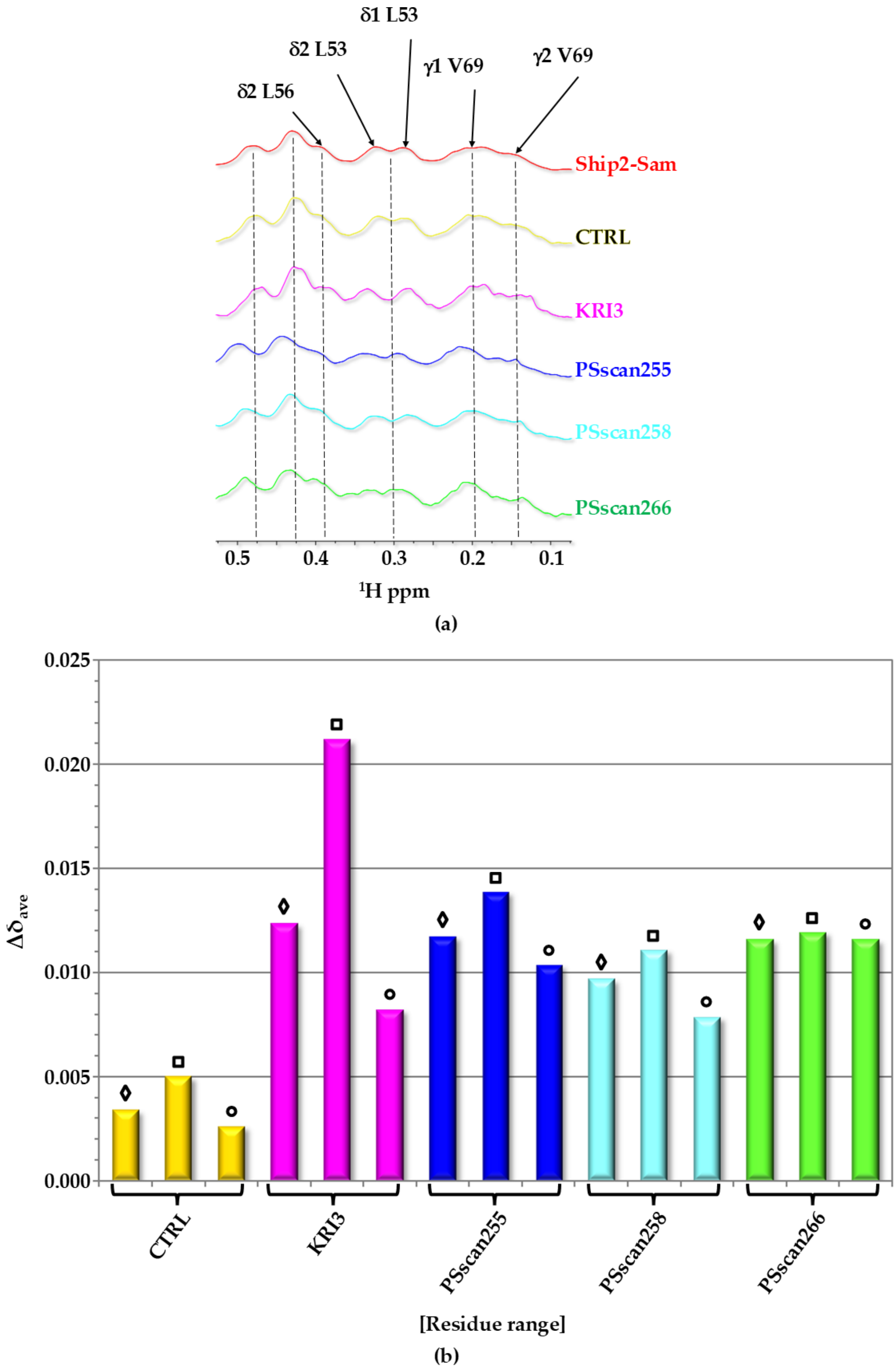

Figure S10. (**a**) Screening by 1D [^{1}H] NMR. Expansion of the aliphatic regions of the 1D [^{1}H] NMR spectra of Ship2-Sam in the apo form (27 μM concentration) (red) and in the presence of the different indicated peptides (273 μM each). (**b**) Comparison of average CSP ($\Delta\delta_{ave}$/ppm) values for different peptides. Data are shown for the whole Ship2-Sam sequence ("◊" residues from L24 to K86. For W32 and W50 CSP evaluation included backbone NH and side chain NHε1 peaks), the ML interface ("□" segment H47-E66) and the region external to ML ("○" residues L24-V46 and A67-K86).

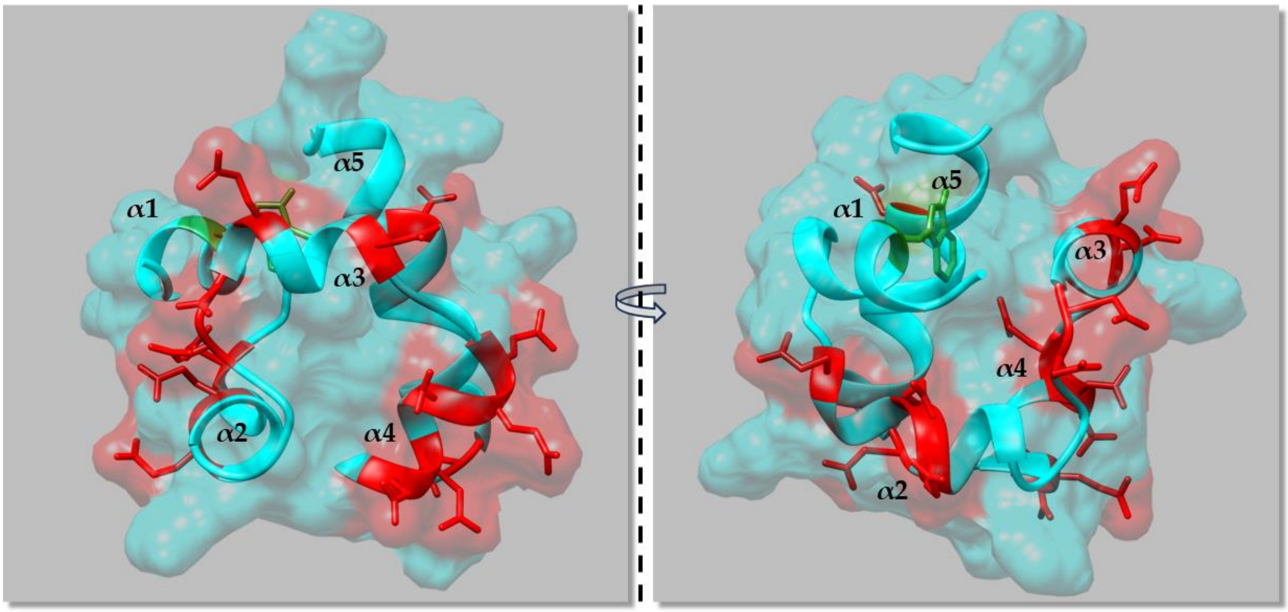

Figure S11. Ship2-Sam NMR structure (conformer n.1 pdb entry code: 2K4P [4]) in ribbon and clear surface representation. The side chains of the negatively charged Glu and Asp residues (red) and of Trp32 (green) are shown. Two different protein orientations are presented in the left and right panels.

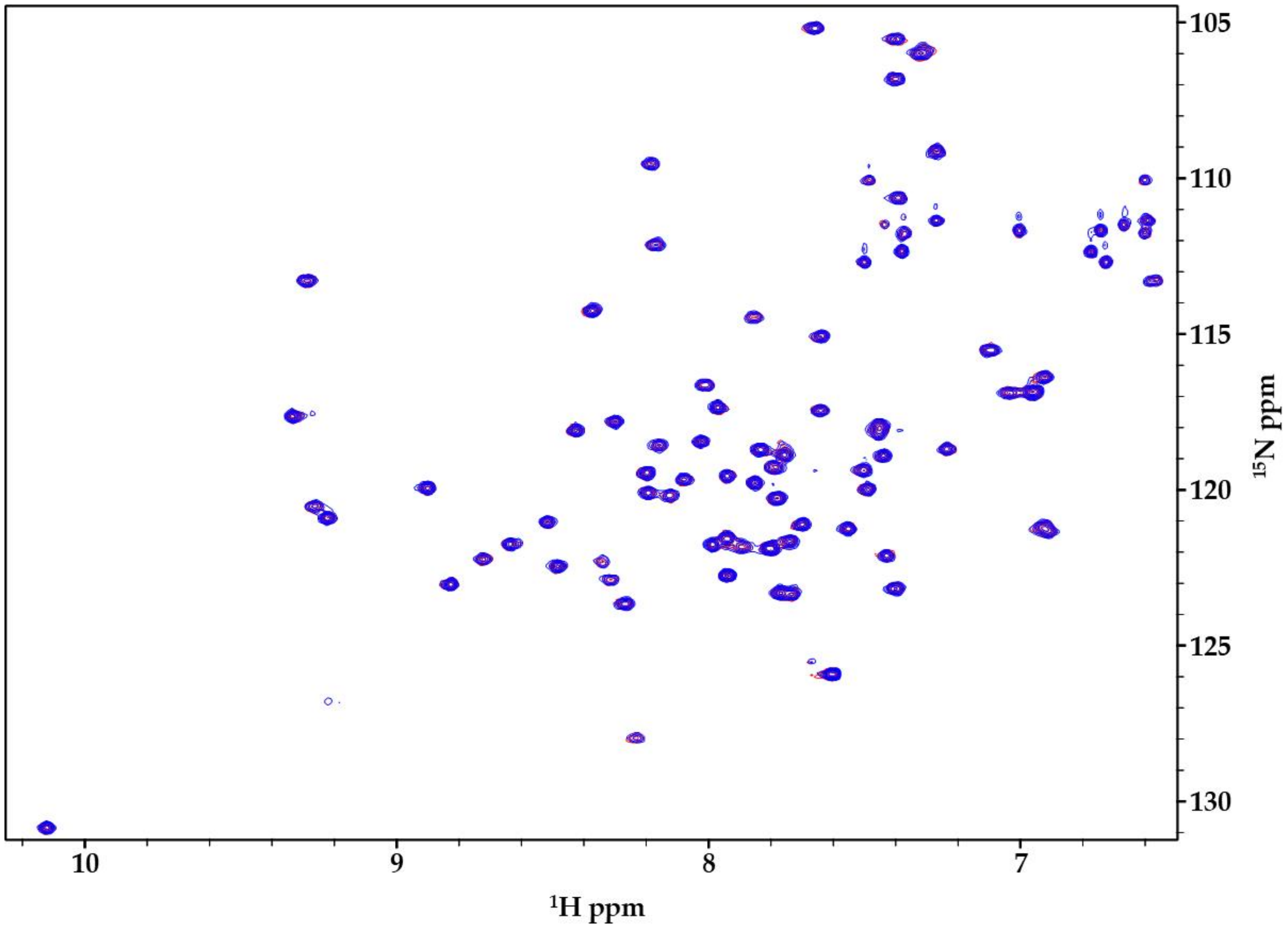

Figure S12. Comparison of [^{1}H-^{15}N] HSQC spectra of EphA2-Sam (33 μM concentration in PBS pH 7.45) in the absence (red) and in the presence (blue) of PSscan255 peptide (300 μM).

PSscan255

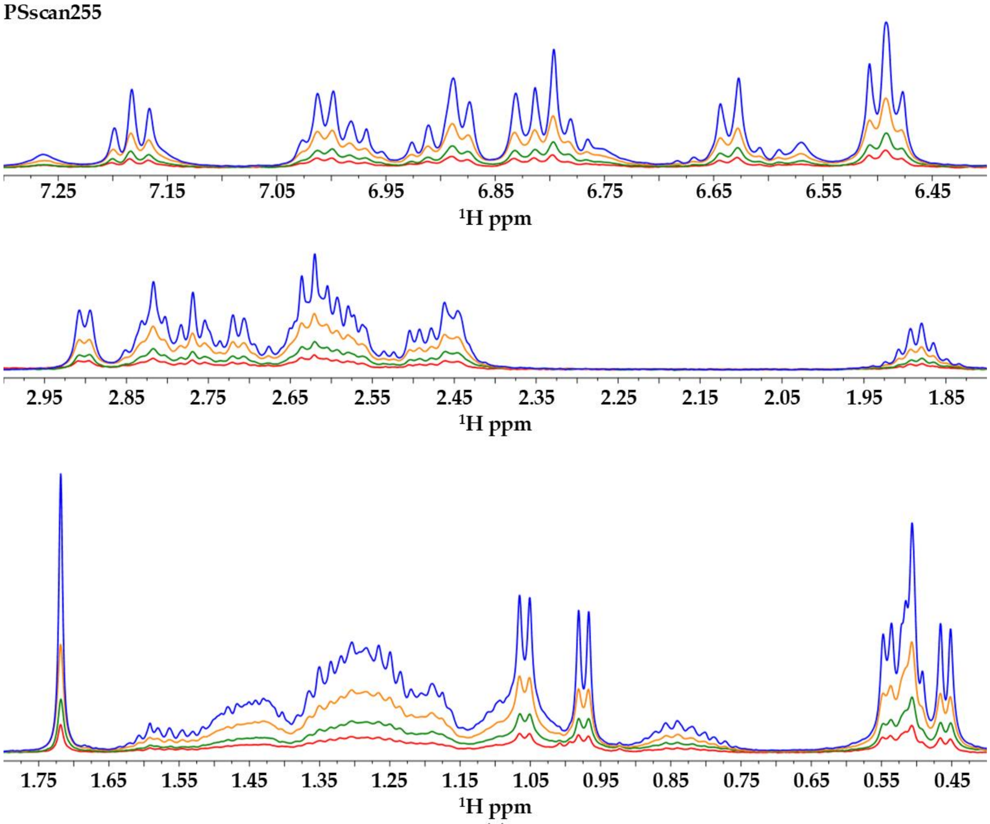

(a)

PSscan258

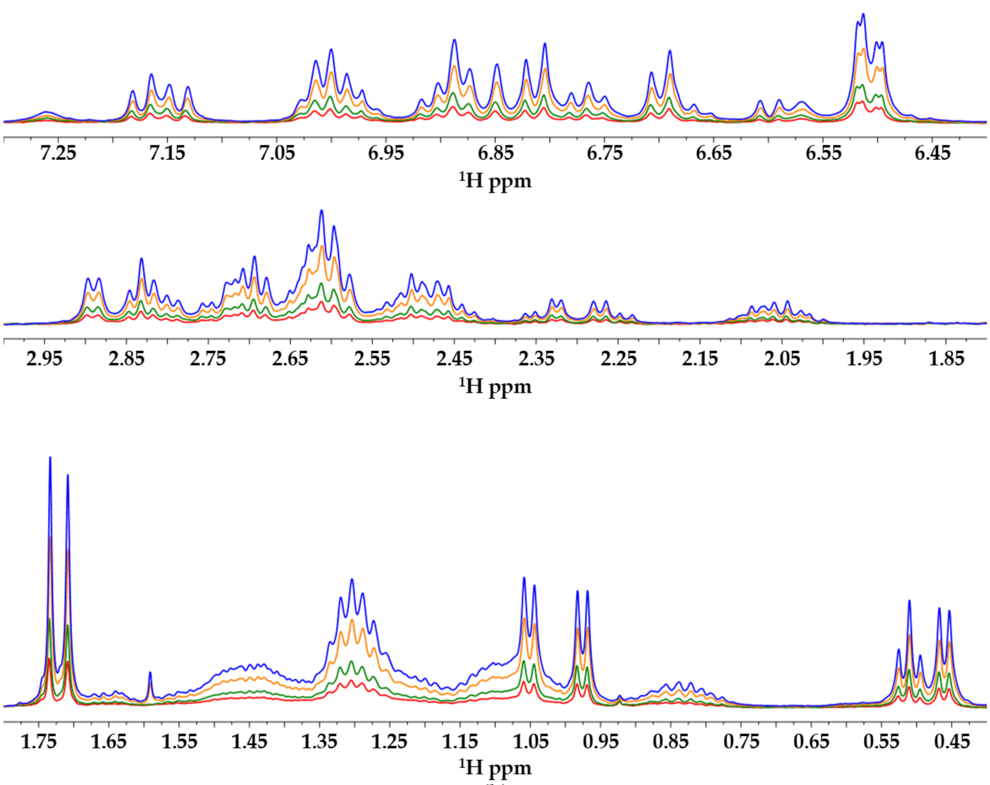

(b)

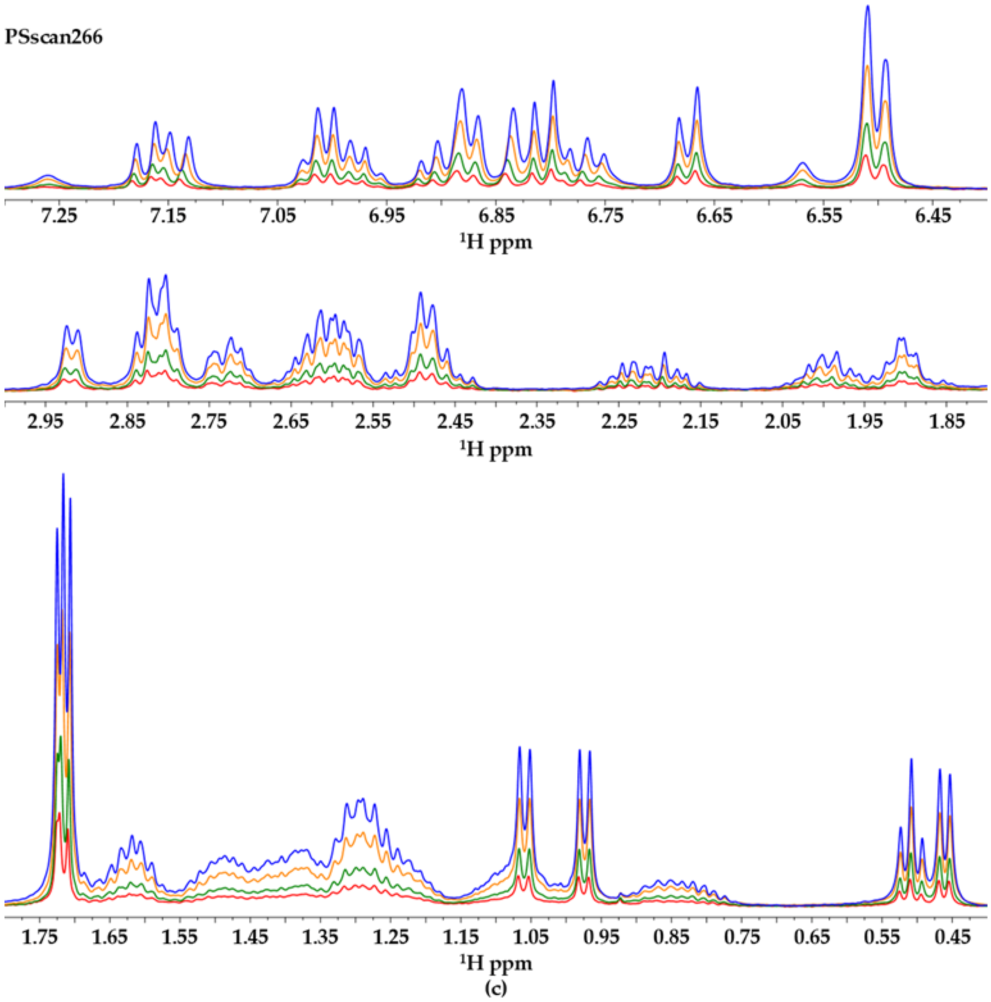

Figure S13. Overlay of 1D [^{1}H] NMR spectra in PBS/D_2O (90/10 - v/v) of (**a**) PSscan255, (**b**) PSscan258, (**c**) PSscan266 at 50 (red), 100 (green), 200 (orange), 300 (blue) μM concentrations. Three different expansions are shown: 7.3-6.4 ppm (i.e., H_N/aromatic region -upper panels); 3.0-1.8 ppm (i.e., aliphatic region - middle panels); 1.8-0.4 ppm (i.e., aliphatic region - bottom panels).

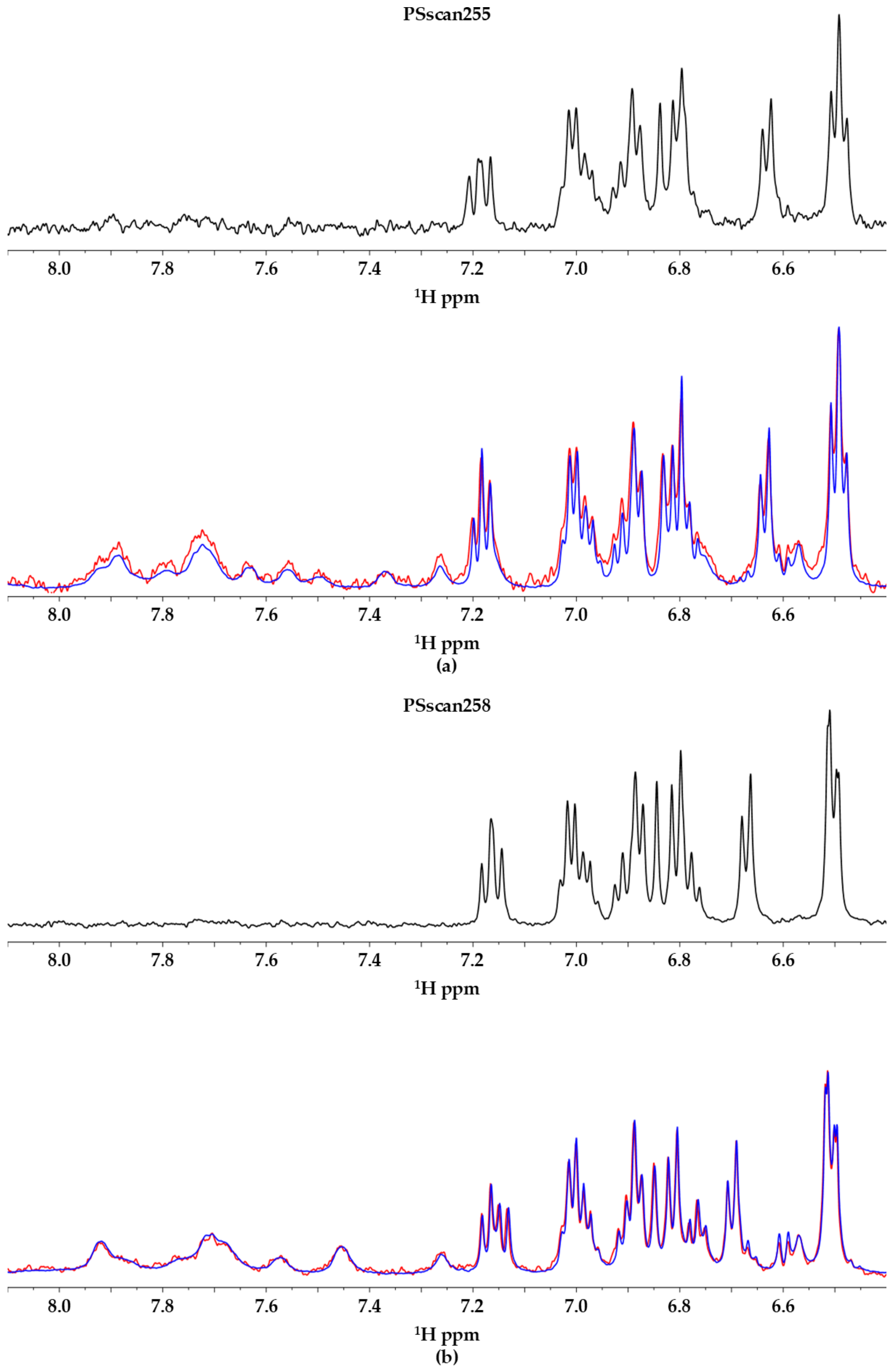

Figure S14. (**a**) The spectrum of PSscan255 peptide (33 µM concentration) in PBS/D_2O (33/67 - v/v) (black) is shown on the top panel. The comparison of the amide / aromatic proton regions of 1D [^{1}H] NMR spectra recorded in PBS/D_2O (90/10 - v/v) for PSscan255 at 50 (red) and 300 (blue) µM concentrations is reported in the bottom panel. (**b**) Comparison of the amide / aromatic proton regions of 1D [^{1}H] NMR spectra recorded in PBS/D_2O (90/10 - v/v) for PSscan258 at 50 (red) and 300 (blue) µM concentrations. The spectrum of PSscan258 peptide (100 µM concentration) in PBS/D_2O (33/67 - v/v) (black) is shown on top. In the lower panels (**a, b**) peak intensities are scaled based on the more concentrated peptide samples (i.e., 300 µM concentration).

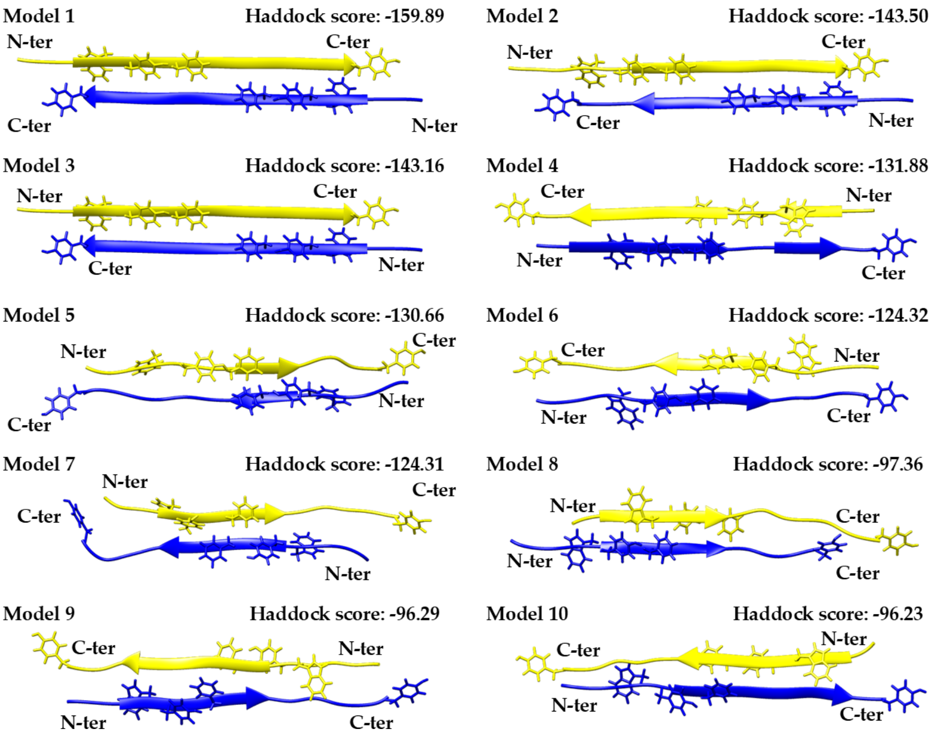

Figure S15. The best 10 Haddock [5, 6] solutions for the PSscan266 dimer in an extended conformation. Chains A and B in the PSscan266 dimers are colored blue and yellow, respectively. The side chains of diverse aromatic residues (Tyr, Trp, and Phe) are shown in a neon representation. Haddock scores are indicated: lowest values point out better solutions.

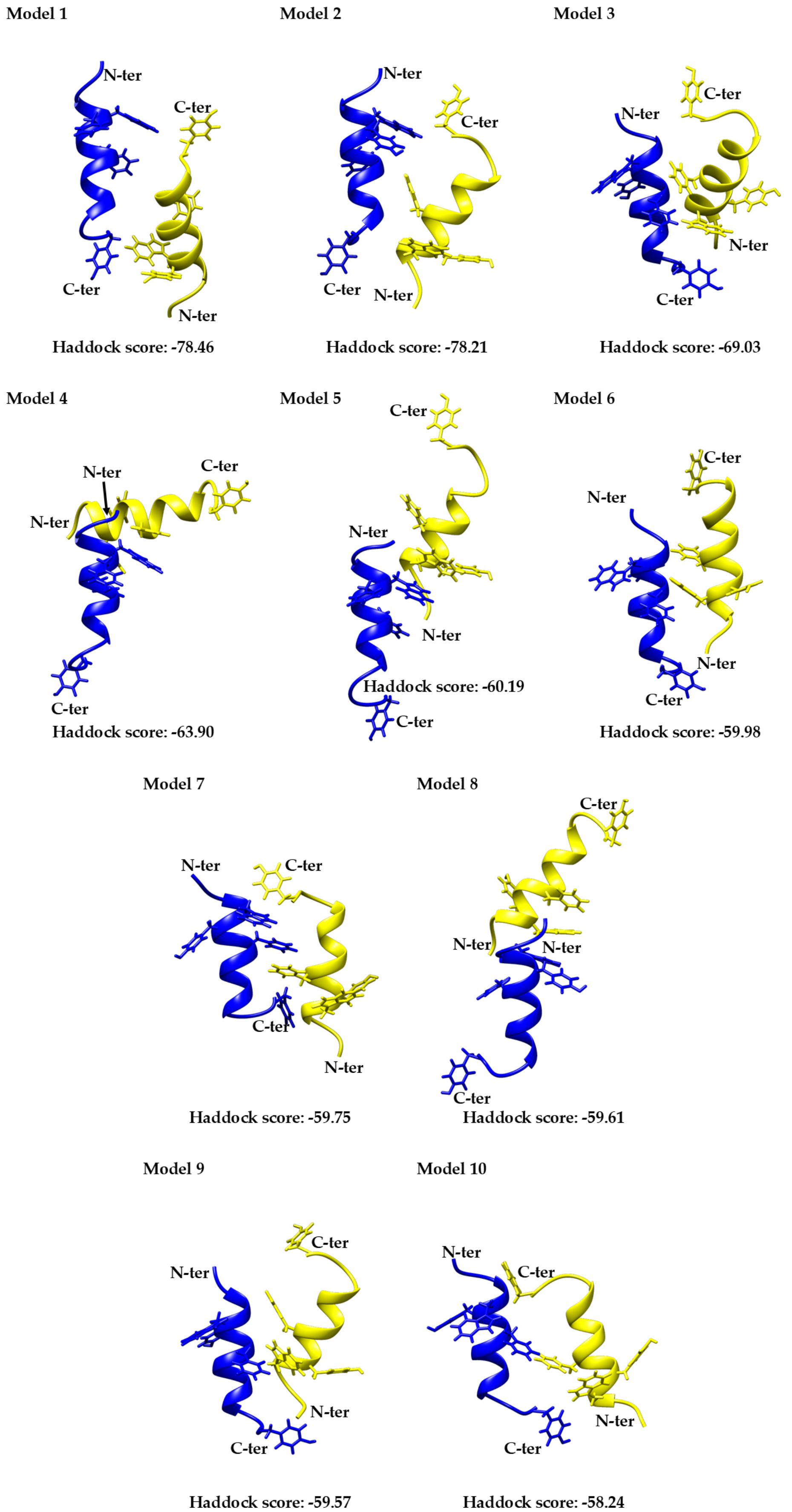

Figure S16. The best 10 Haddock [5, 6] solutions for the PSscan266 dimer in a helical conformation. Chain A and B in the PSscan266 dimers are colored blue and yellow, respectively. The side chains of diverse aromatic (Tyr, Trp, and Phe) residues are shown in a neon representation with heavy atoms and hydrogens. Haddock scores are indicated: lowest values point out better solutions.

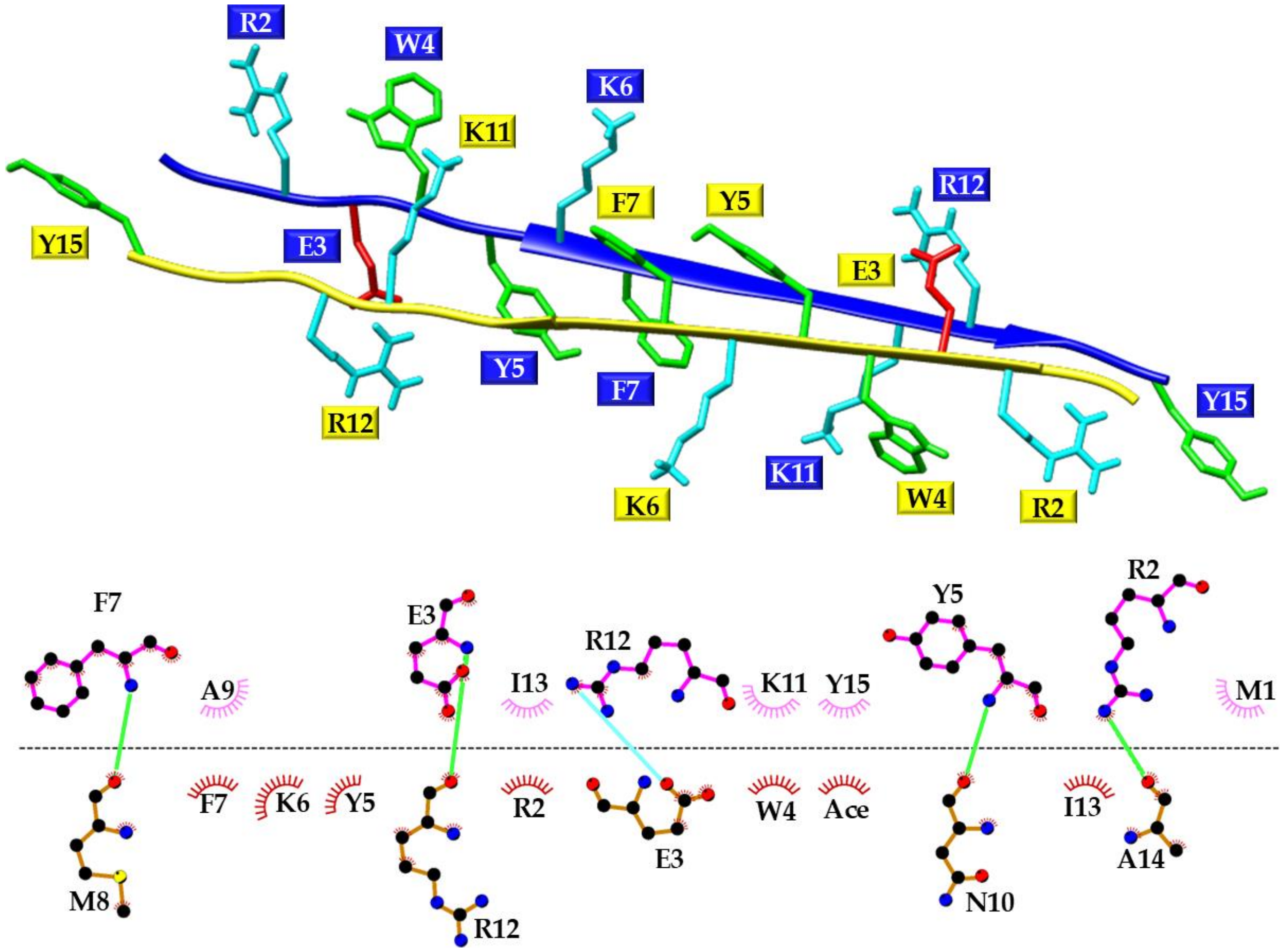

Figure S17. (Upper panel) One representative Haddock [5, 6] generated model (i.e., the one with the lowest Haddock score) of PSscan266 dimer in an extended conformation. The two PSscan266 strands are colored blue (chain A), and yellow (chain B); the side chains of aromatic (Tyr, Trp, and Phe -green-), positively (Lys, Arg -blue-), and negatively (Glu -red-) charged residues are shown in a neon representation with only heavy atoms and polar hydrogens, and labeled with the one-letter amino acid codes and residue numbers (blue and yellow rectangles for chains A and B, respectively). (Bottom panel) LigPlot+ [7, 8] diagram of intermolecular contacts. Residues involved in self-recognition are labelled. H-bonds and salt bridges are highlighted with green and cyan solid lines, respectively. Residues providing non-bonded interactions in each peptide unit are represented by red and magenta crescents with bristles. “Ace” stands for N-terminal Acetyl protecting group.

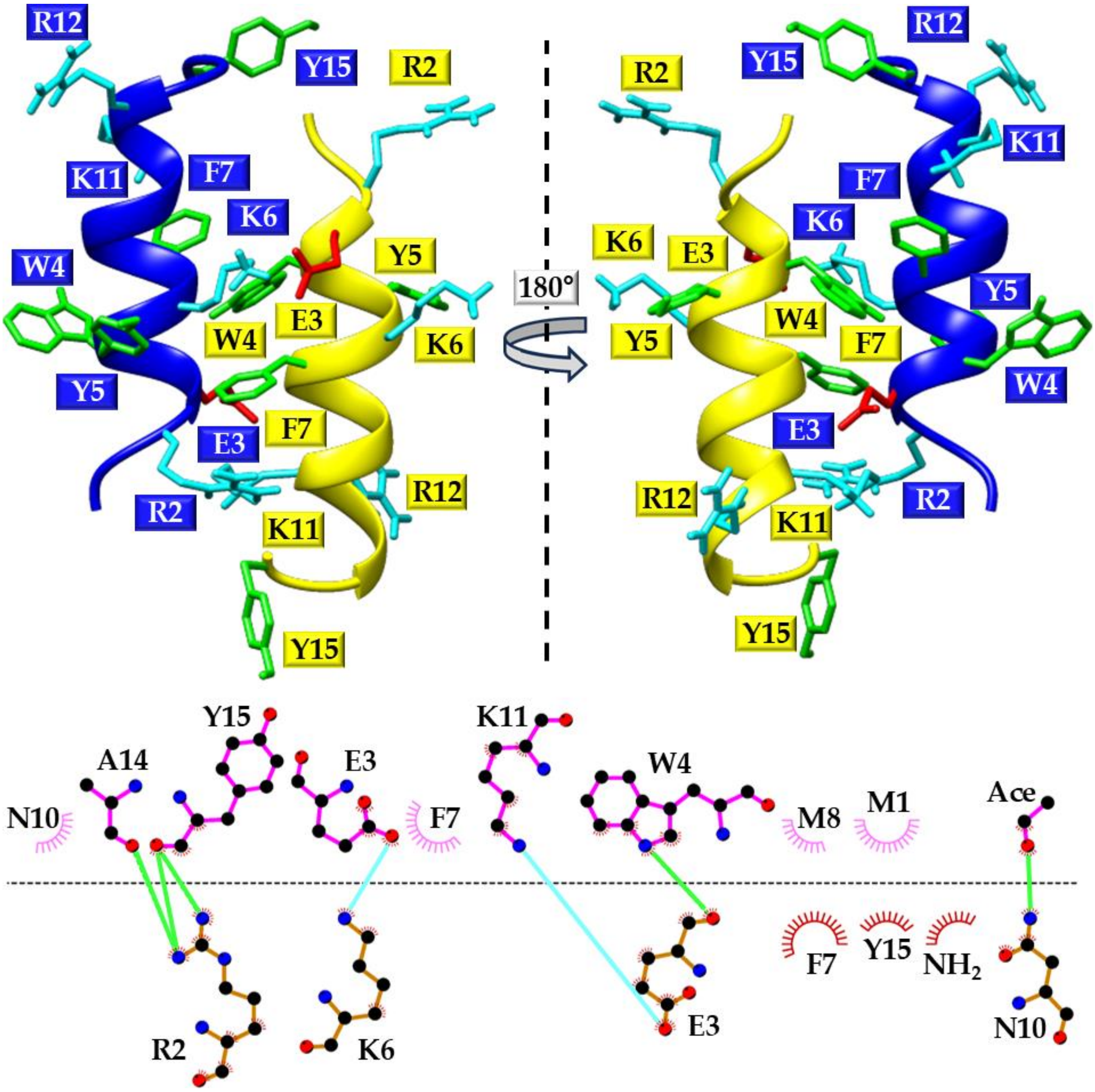

Figure S18. (Upper panel) One representative Haddock [5, 6] solution (i.e., number 6 in order of Haddock scores) for the PSscan266 dimer in a helical conformation. The A and B PSscan266 chains are colored blue and yellow, respectively and two diverse orientations are shown. The side chains of aromatic (Tyr, Trp, and Phe), positively (Lys, Arg), and negatively (Glu) charged residues are shown in green, cyan and red, respectively and labeled with the one-letter amino acid codes and sequence numbers. (Bottom panel) LigPlot+ [7, 8] diagram of intermolecular contacts. H-bonds and salt bridges are highlighted with green and cyan solid lines, respectively. Red and magenta crescents with bristles point out non-bonded contacts. "Ace" stands for the N-terminal Acetyl protecting group, and "NH_2" for the C-terminal amide group.

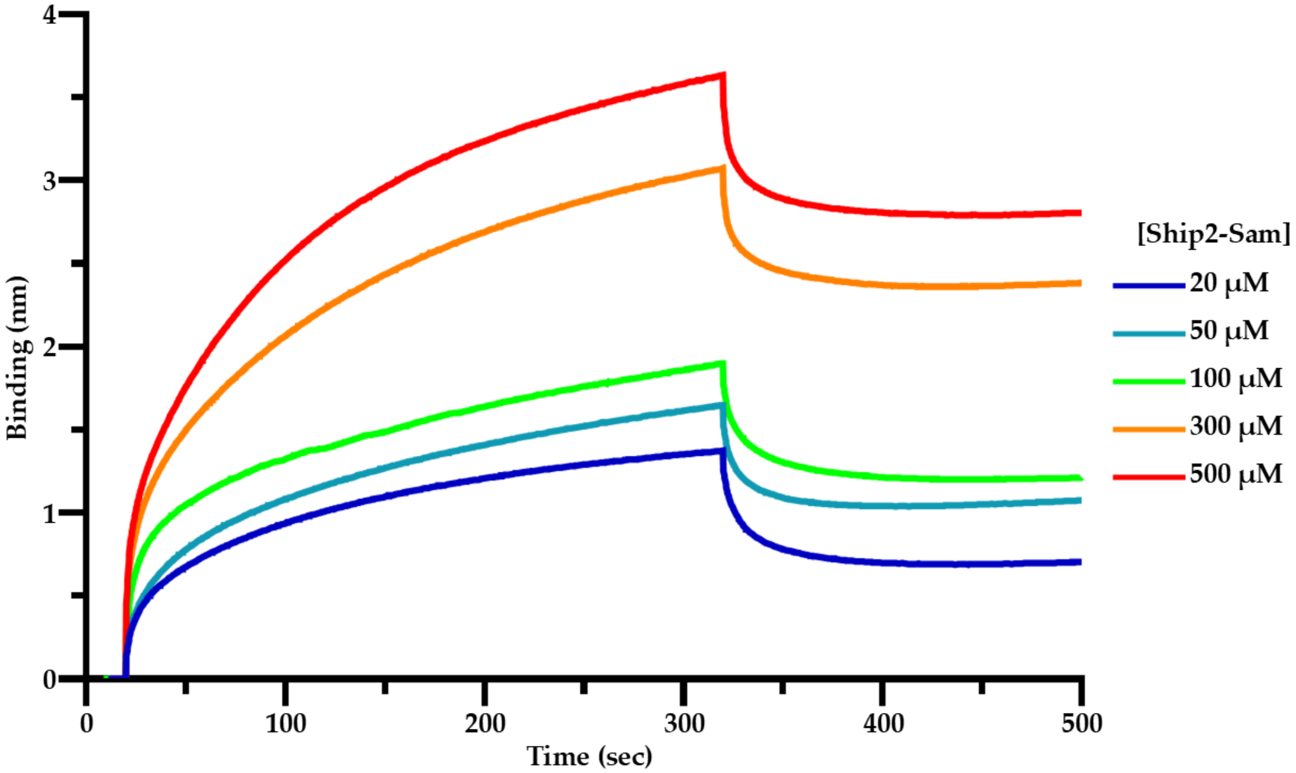

Figure S19. Interaction between immobilized PSscan255 peptide and Ship2-Sam: BLI traces at different protein concentrations.

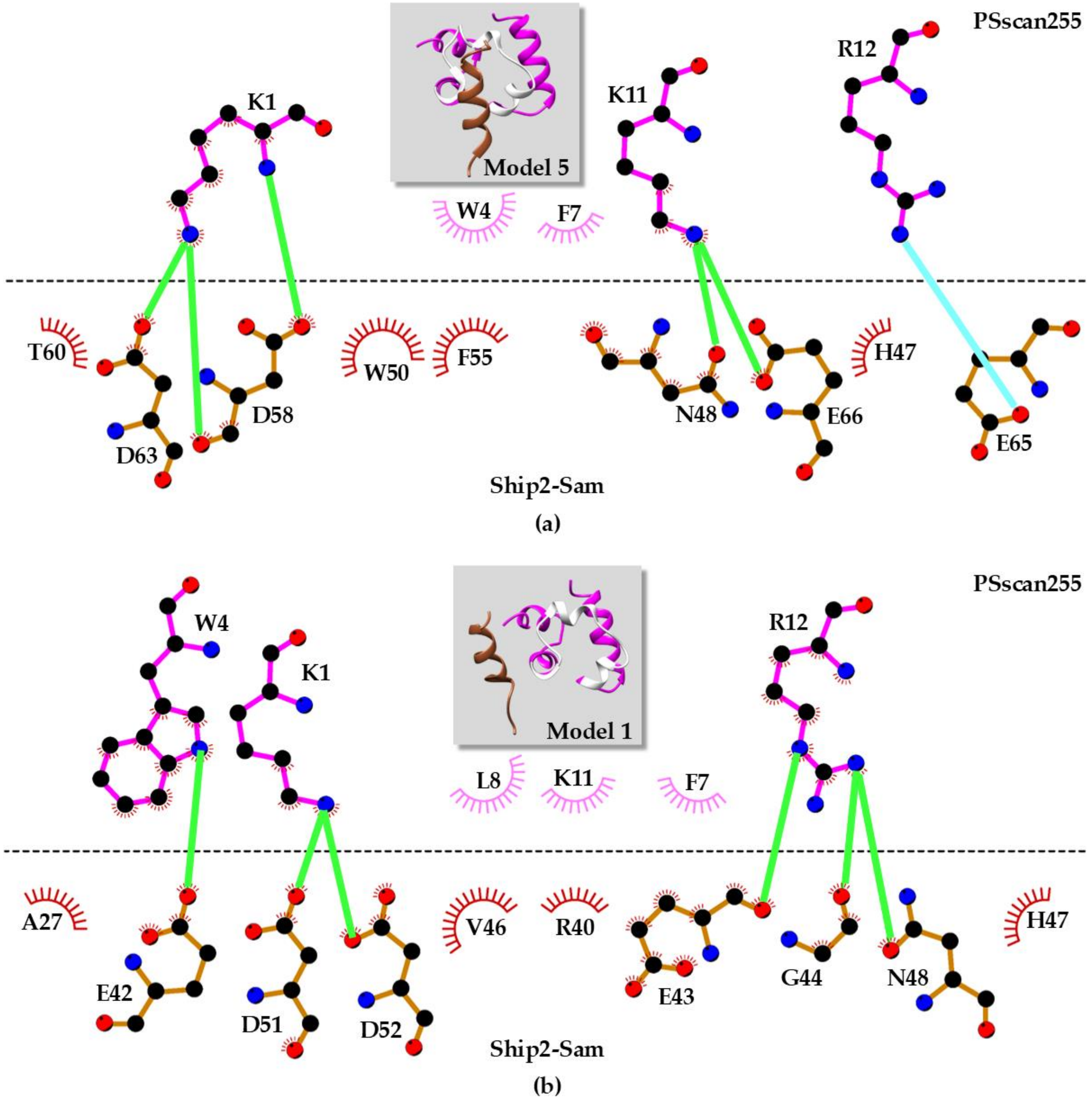

Figure S20. 2D diagrams of intermolecular interactions generated by LigPlot+ [7, 8] for two of the best Ship2-Sam/PSscan255 docking poses, that are shown in ribbon representations in the middle inserts with the PSscan255 peptide colored orange and Ship2-Sam colored magenta with the ML surface in white. (**a**) Haddock pose n. 5 characterized by the PSscan255 peptide interacting with the ML surface of Ship2-Sam. (**b**) Best solution in terms of Haddock score (i.e., n. 1) with the PSscan255 peptide disposed laterally at one edge of the ML interface. (**a, b**) Ship2-Sam and PSscan255 peptide residues involved in non-bonded interactions are labelled as well and represented by red and pink crescents with bristles. Green and cyan lines indicate H-bonds, and salt-bridges, respectively.

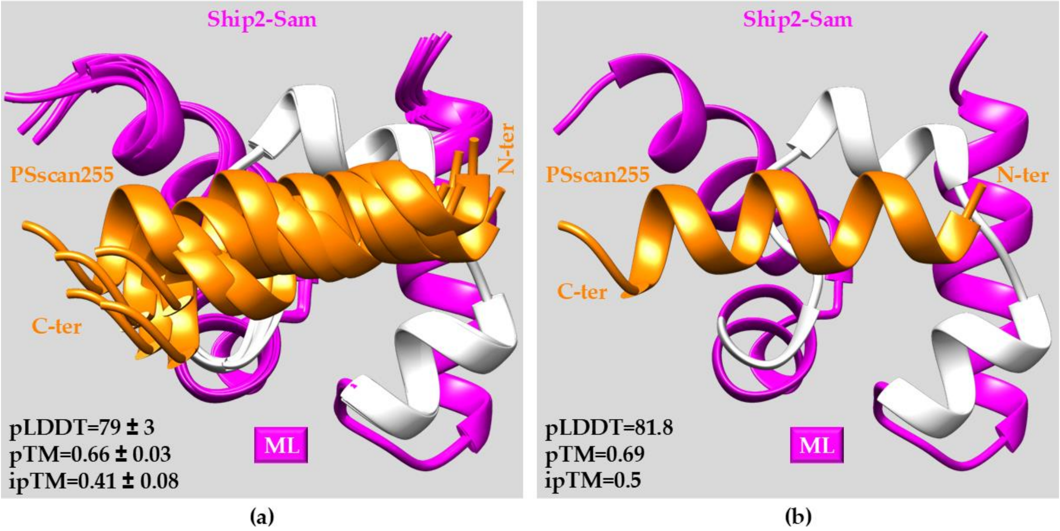

Figure S21. AlphaFold2 (AF2) [9, 10] models for the PSscan255 peptide in complex with Ship2-Sam. (**Left**) Superposition on the backbone atoms of the best five models predicted by AlphaFold2. Averaged pLDDT, pTM, and ipTM scores over the 5 models and corresponding standard deviations are indicated. (**Right**) Representative AF2 model with its pLDDT, pTM, and ipTM scores. Both Ship2-Sam and PSscan255 are reported in a ribbon representation where Ship2-Sam is colored magenta with the ML interface highlighted in white whereas the peptide is colored orange.

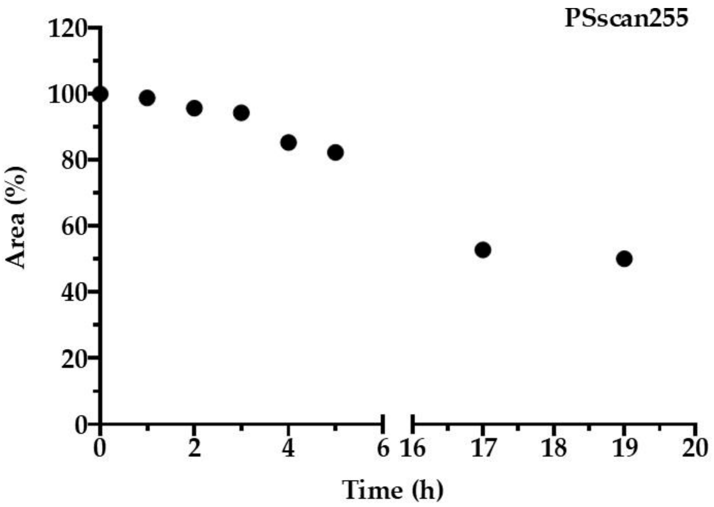

(a)

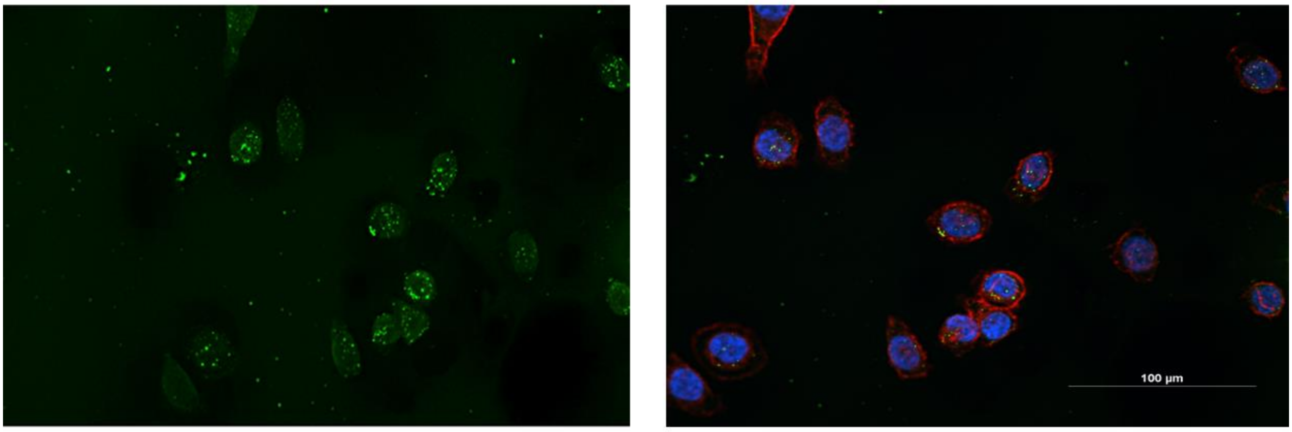

(b)

Figure S22. (**a**) Stability in FBS (Fetal Bovine Serum) of the PSscan255 peptide. (**b**) Characteristic images, by means of deconvolution microscopy, showing FITC-TAT-PSscan255 uptake in PC-3 cells. (Left) PC-3 cells were treated with FITC-TAT-PSscan255 at 50 µM concentration for 4 hours. FITC-TAT-PSscan255 is displayed in green punctate structures. (Right) Overlay of FITC-TAT-PSscan255 (green), nuclei marked with Hoechst (blue), and actin filaments marked with phalloidin (red).

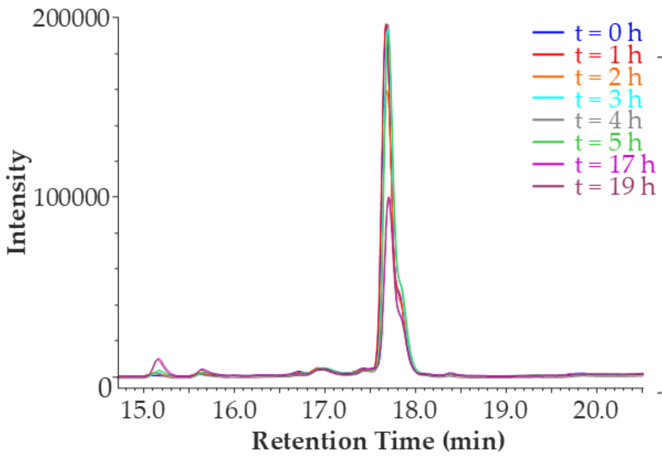

(a)

Sample time (h)	Retention time (min)	Area	Area (%)
0	17.675	1.63E+06	100
1	17.673	1.61E+06	98.8
2	17.676	1.56E+06	95.7
3	17.677	1.54E+06	94.5
4	17.677	1.39E+06	85.3
5	17.679	1.34E+06	82.2
17	17.675	8.61E+05	52.8
19	17.675	8.18E+05	50.2

(b)

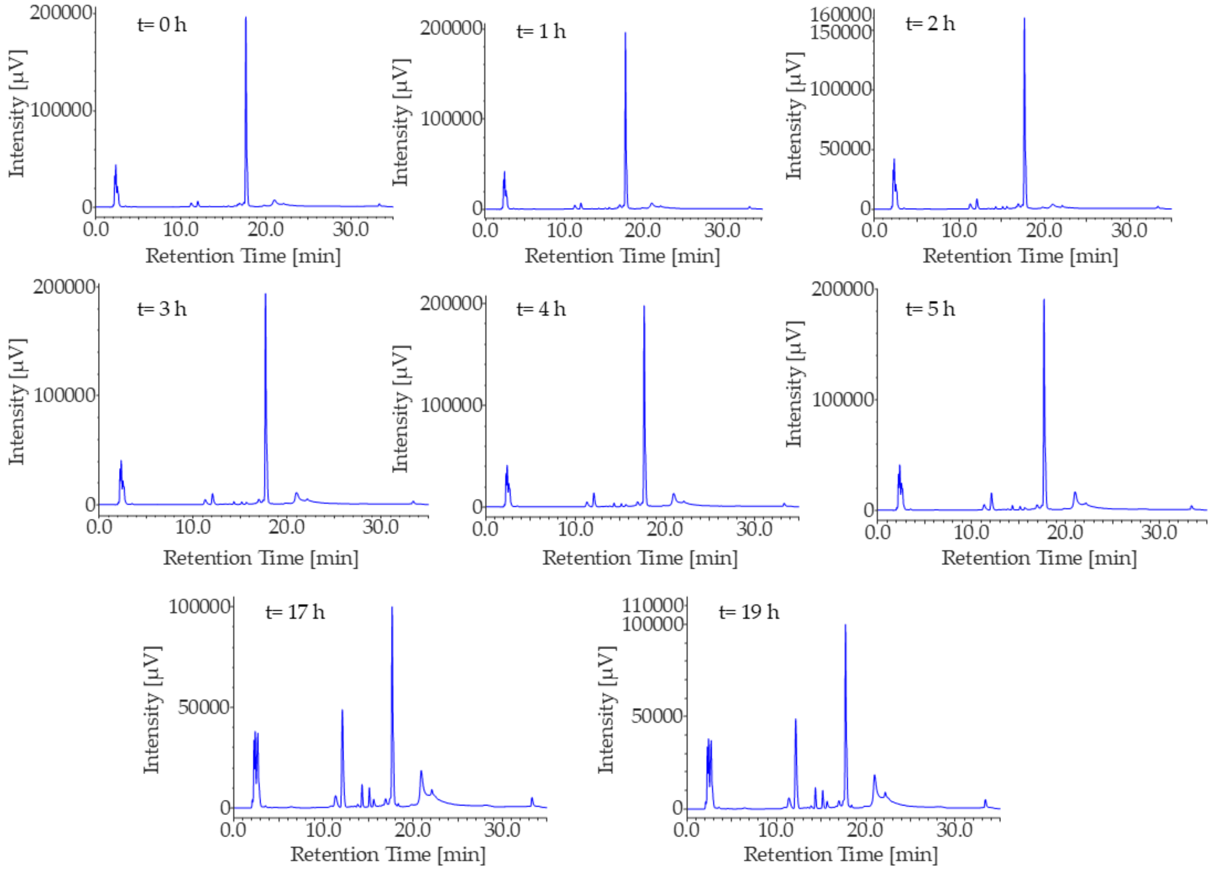

(c)

Figure S23. (**a**) Overlay of the HPLC traces of the PSscan255 peak in the serum stability (for one representative experiment). (**b**) Table with parameters used to calculate the percentages of area. (**c**) Single chromatograms of serum stability samples (in full time format) at the different indicated times.

Sample Name :3
Sample ID :U944VHI060-3
Time Processed :2:31:58
Month-Day-Year Processed :11/01/2022

Pump A : 0.065% trifluoroacetic in 100% water (v/v)
Pump B : 0.05% trifluoroacetic in 100% acetonitrile (v/v)
Total Flow:1 ml/min
Wavelength:220 nm
<<LC Time Program>>

Time	Module	Command	Value
0.01	Pumps	B.Conc	5
25.00	Pumps	B.Conc	65
25.01	Pumps	B.Conc	95
27.00	Pumps	B.Conc	95
27.01	Pumps	B.Conc	5
35.00	Pumps	B.Conc	5
35.01	Controller	Stop	

<<Column Performance>>
<Detector A>
Column :Inertsil ODS-3 4.6 x 250 mm
Equipment: ZJ19010140

<Chromatogram>

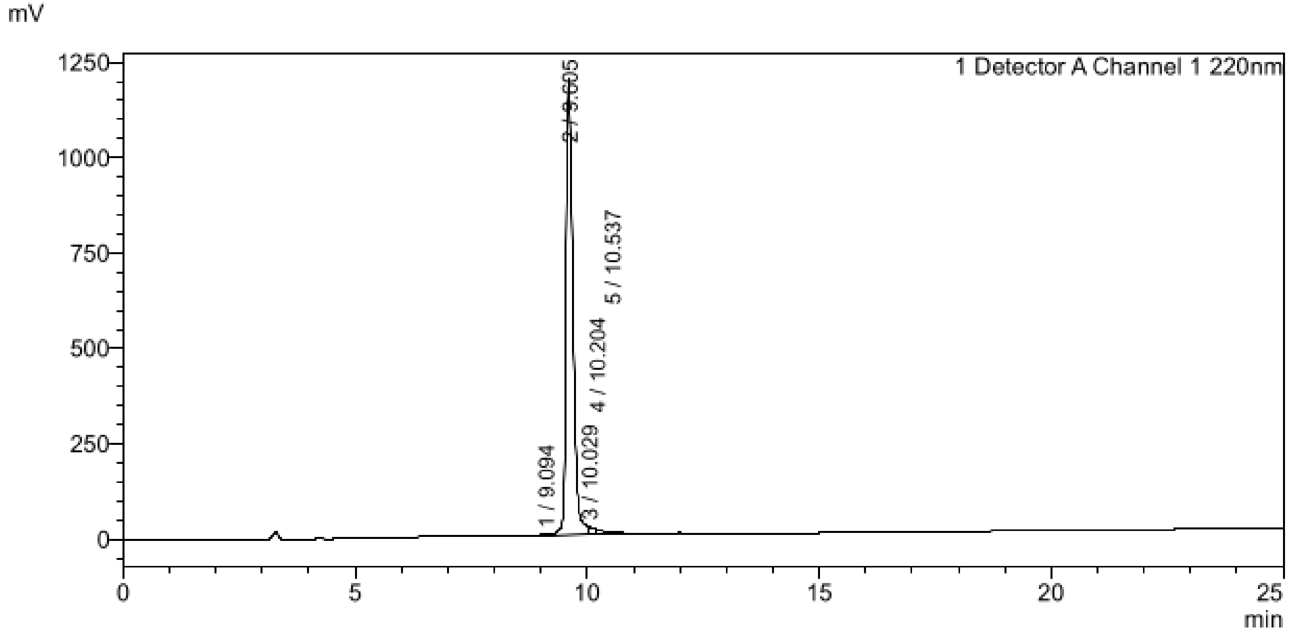

<Peak Table>

Detector A Channel 1 220nm

Peak#	Ret. Time	Area	Height	Area%
1	9.094	46316	4063	0.359
2	9.605	12425720	1196247	96.196
3	10.029	181611	22048	1.406
4	10.204	125876	13926	0.974
5	10.537	137592	7515	1.065
Total		12917115	1243798	100.000

Figure S24. HPLC profile: PSscan255 (purity 96.2%). Data provided by GenScript.

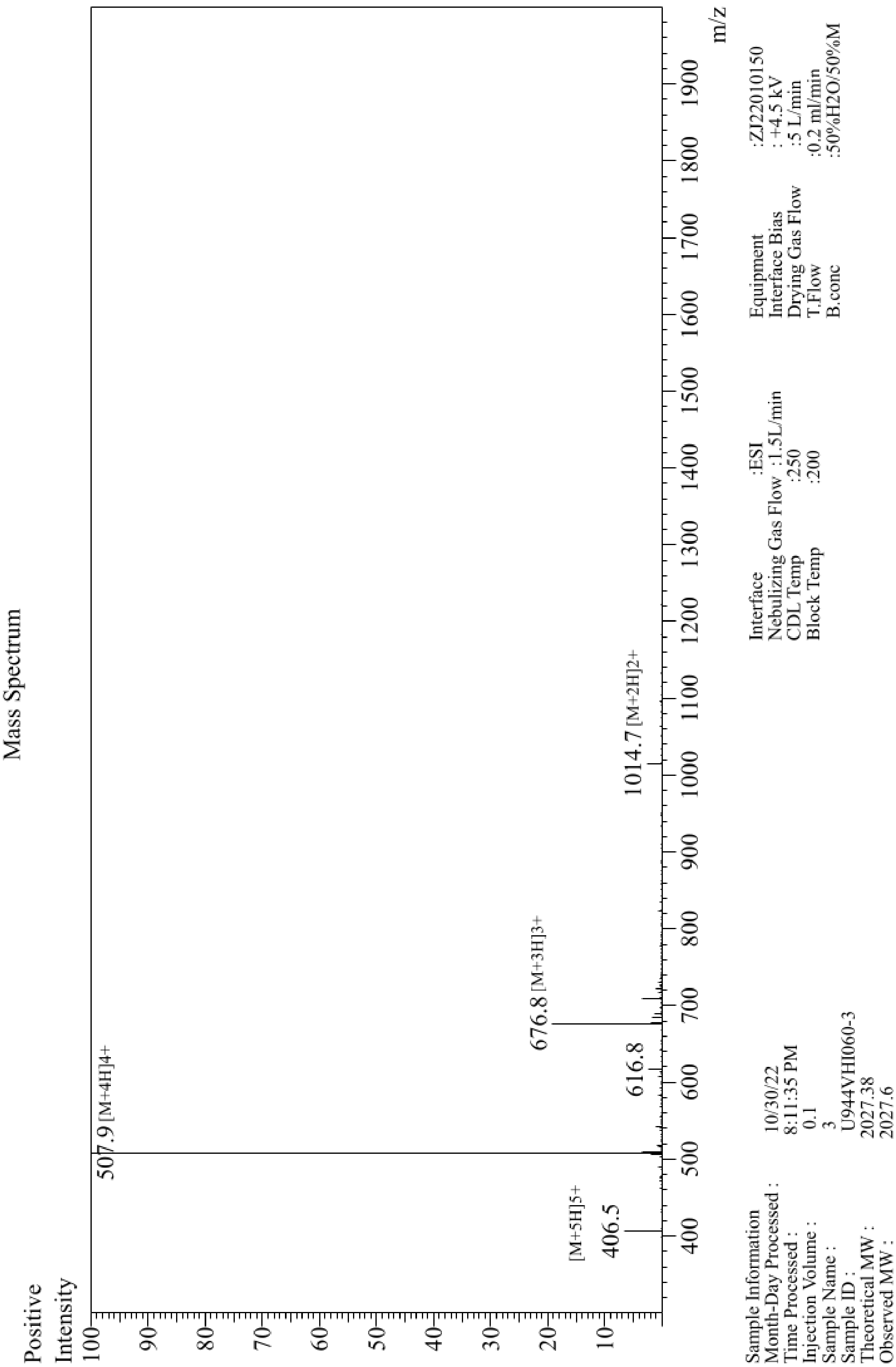

Figure S25. Mass spectrum: PSscan255. Data provided by GenScript.

Sample Name :3
Sample ID :U9249YHPG0-5
Time Processed :0:48:18
Month-Day-Year Processed :07/24/2023

Pump A : 0.065% trifluoroacetic in 100% water (v/v)
Pump B : 0.05% trifluoroacetic in 100% acetonitrile (v/v)
Total Flow:1 ml/min
Wavelength:220 nm
<<LC Time Program>>

Time	Module	Command	Value
0.01	Pumps	B.Conc	5
25.00	Pumps	B.Conc	65
25.01	Pumps	B.Conc	95
27.00	Pumps	B.Conc	95
27.01	Pumps	B.Conc	5
35.00	Pumps	B.Conc	5
35.01	Controller	Stop	

<<Column Performance>>
<Detector A>
Column :Inertsil ODS-SP 4.6 x 250 mm
Equipment: GR11010440

<Chromatogram>

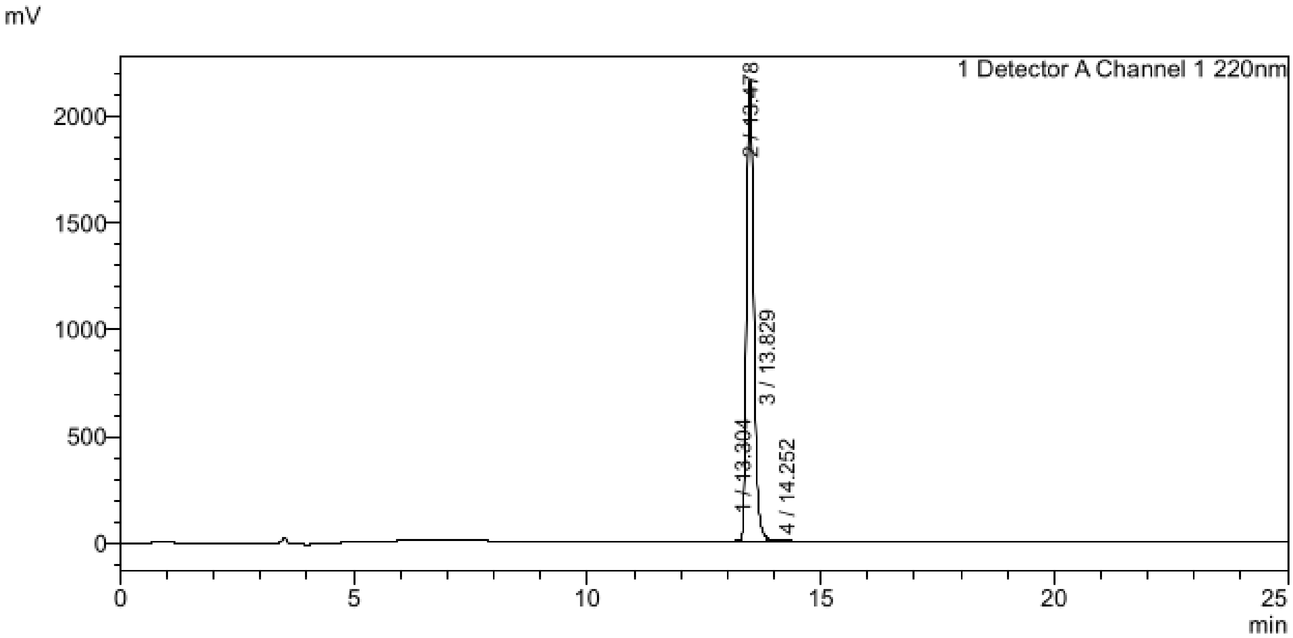

<Peak Table>

Detector A Channel 1 220nm

Peak#	Ret. Time	Area	Height	Area%
1	13.304	222390	24257	0.943
2	13.478	22922439	2156486	97.162
3	13.829	246981	22356	1.047
4	14.252	200290	6673	0.849
Total		23592100	2209772	100.000

Figure S26. HPLC profile: PSscan258 (purity 97.2%). Data provided by GenScript.

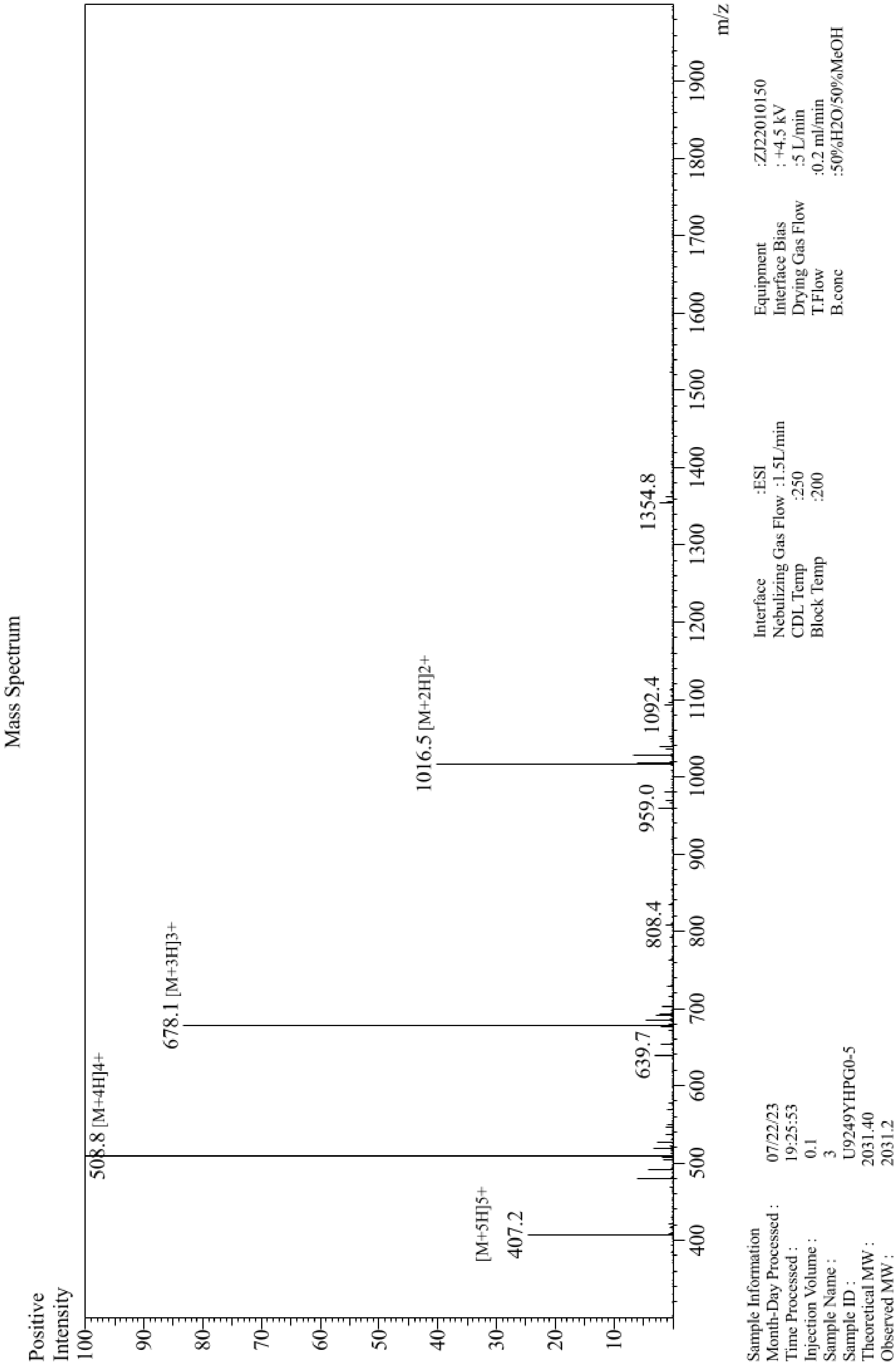

Figure S27. Mass spectrum: PSscan258. Data provided by GenScript.

Sample Name :1
Sample ID :U9249YHPG0-1
Time Processed :21:57:31
Month-Day-Year Processed :07/19/2023

Pump A : 0.065% trifluoroacetic in 100% water (v/v)
Pump B : 0.05% trifluoroacetic in 100% acetonitrile (v/v)
Total Flow:1 ml/min
Wavelength:220 nm
<<LC Time Program>>

Time	Module	Command	Value
0.01	Pumps	B.Conc	5
25.00	Pumps	B.Conc	65
25.01	Pumps	B.Conc	95
27.00	Pumps	B.Conc	95
27.01	Pumps	B.Conc	5
35.00	Pumps	B.Conc	5
35.01	Controller	Stop	

<<Column Performance>>
<Detector A>
Column :Inertsil ODS-SP 4.6 x 250 mm
Equipment: ZJ19010325

<Chromatogram>

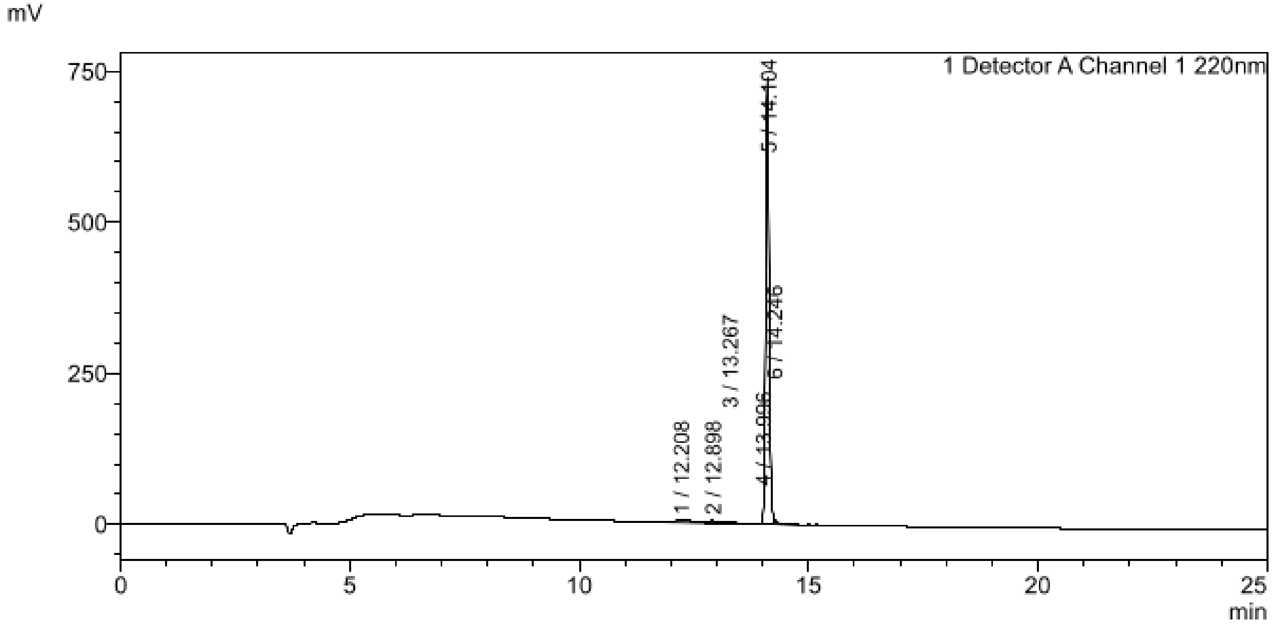

<Peak Table>

Detector A Channel 1 220nm

Peak#	Ret. Time	Area	Height	Area%
1	12.208	76970	2198	1.830
2	12.898	47250	3632	1.124
3	13.267	18220	1220	0.433
4	13.996	8230	3864	0.196
5	14.104	4006678	738783	95.281
6	14.246	47770	11228	1.136
Total		4205118	760926	100.000

Figure S28. HPLC profile: PSscan266 (purity 95.3%). Data provided by GenScript.

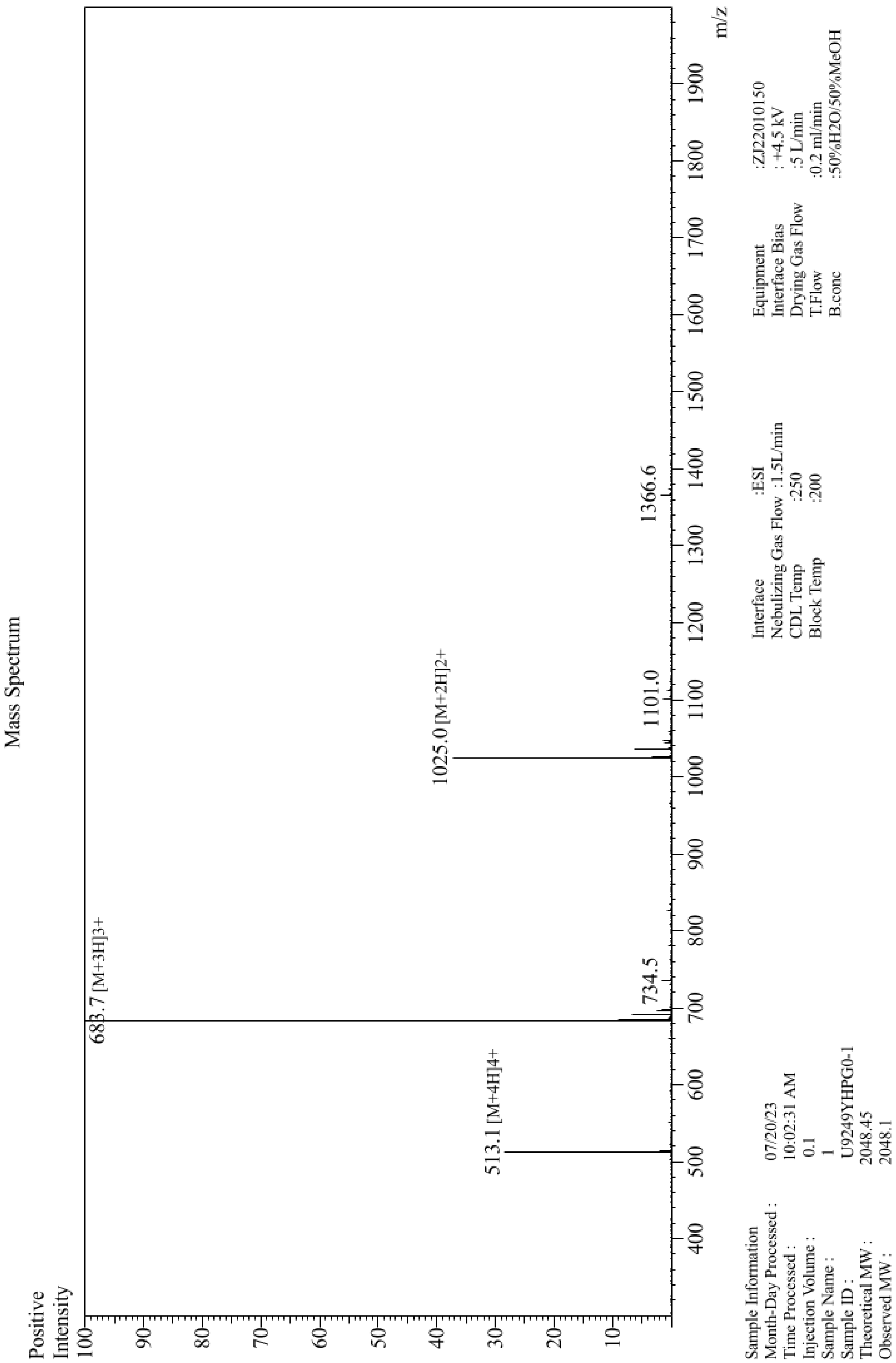

Figure S29. Mass spectrum: PSscan266. Data provided by GenScript.

Sample Name :8
Sample ID :U647A208G0-12
Time Processed :10:47:51 PM
Month-Day-Year Processed :10/03/2023

Pump A : 0.065% trifluoroacetic in 100% water (v/v)
Pump B : 0.05% trifluoroacetic in 100% acetonitrile (v/v)
Total Flow:1 ml/min
Wavelength:220 nm
<<LC Time Program>>

Time	Module	Command	Value
0.01	Pumps	B.Conc	5
25.00	Pumps	B.Conc	65
25.01	Pumps	B.Conc	95
27.00	Pumps	B.Conc	95
27.01	Pumps	B.Conc	5
35.00	Pumps	B.Conc	5
35.01	Controller	Stop	

<<Column Performance>>
<Detector A>
Column :Inertsil ODS-SP 4.6 x 250 mm
Equipment: ZJ19010141

<Chromatogram>

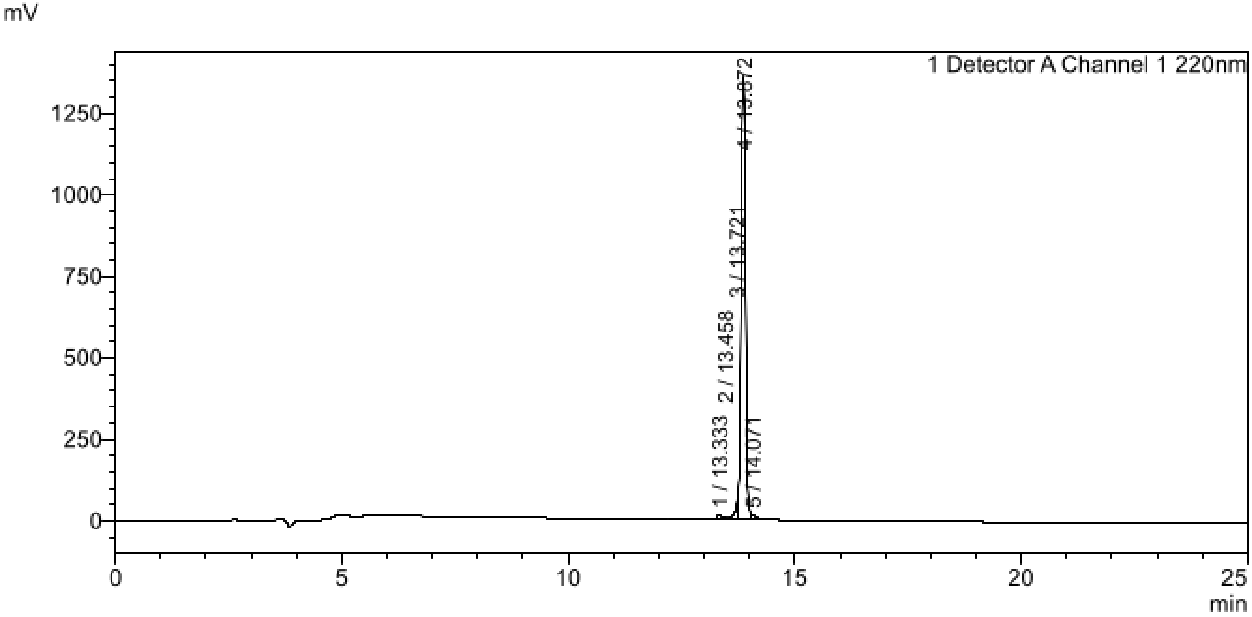

<Peak Table>

Detector A Channel 1 220nm

Peak#	Ret. Time	Area	Height	Area%
1	13.333	77134	14040	0.743
2	13.458	30381	5291	0.293
3	13.721	208060	48998	2.004
4	13.872	9966649	1358404	95.985
5	14.071	101316	15946	0.976
Total		10383540	1442678	100.000

Figure S30. HPLC profile: Biotin-(Peg11)-PSscan255 (purity 96.0%). Data provided by GenScript.

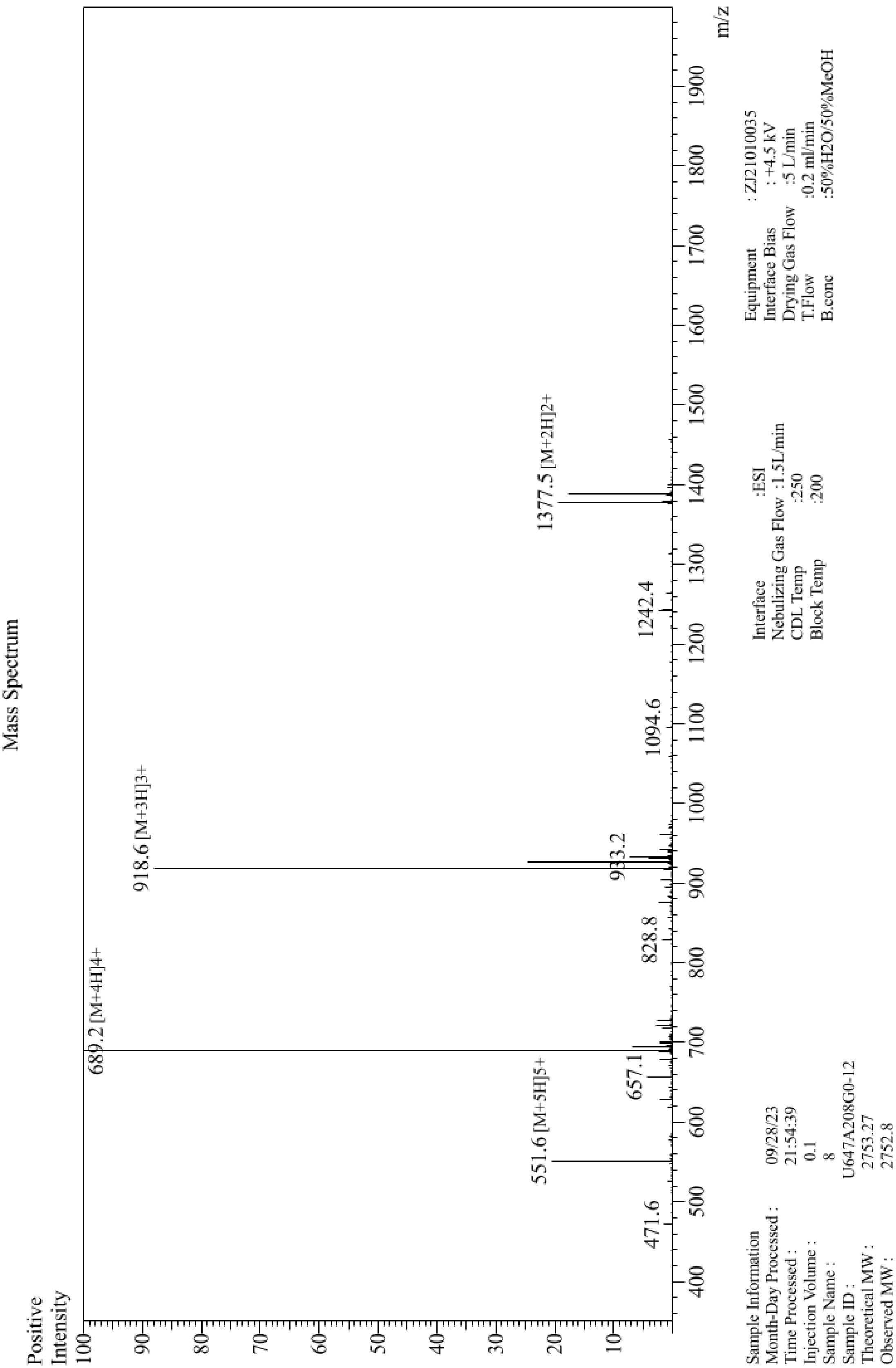

Figure S31. Mass spectrum: Biotin-(Peg11)-PSscan255. Data provided by GenScript.

Sample Name :27
Sample ID :U9249YHPG0-60
Time Processed :22:44:17
Month-Day-Year Processed :08/13/2023

Pump A : 0.065% trifluoroacetic in 100% water (v/v)
Pump B : 0.05% trifluoroacetic in 100% acetonitrile (v/v)
Total Flow:1 ml/min
Wavelength:220 nm
<<LC Time Program>>

Time	Module	Command	Value
0.01	Pumps	Pump A B.Conc	5
10.00	Pumps	Pump A B.Conc	35
35.00	Pumps	Pump A B.Conc	95
37.00	Pumps	Pump A B.Conc	95
37.01	Pumps	Pump A B.Conc	5
45.00	Pumps	Pump A B.Conc	5
45.01	Controller	Stop	

<<Column Performance>>
<Detector A>
Column :Inertsil ODS-SP 4.6 x 250 mm
Equipment: GK11010011

<Chromatogram>

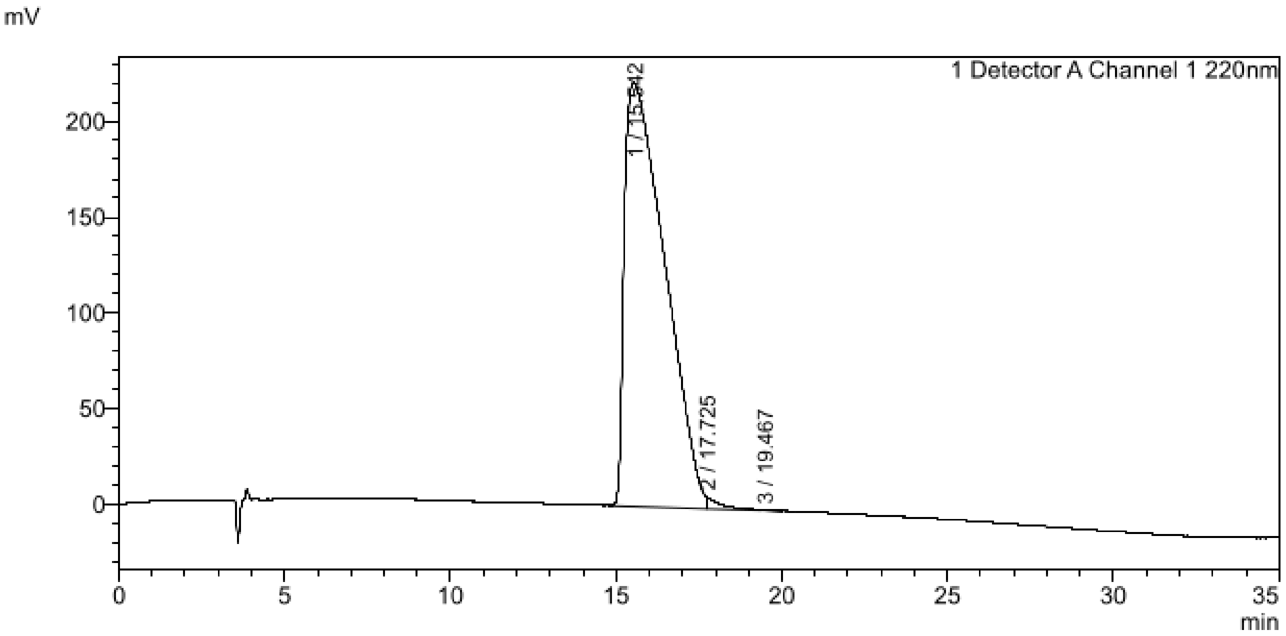

<Peak Table>

Detector A Channel 1 220nm

Peak#	Ret. Time	Area	Height	Area%
1	15.542	18699816	221288	99.029
2	17.725	177565	5957	0.940
3	19.467	5707	305	0.030
Total		18883089	227550	100.000

Figure S32. HPLC profile: FITC-TAT-PSscan255 (purity 99.0%). Data provided by GenScript.

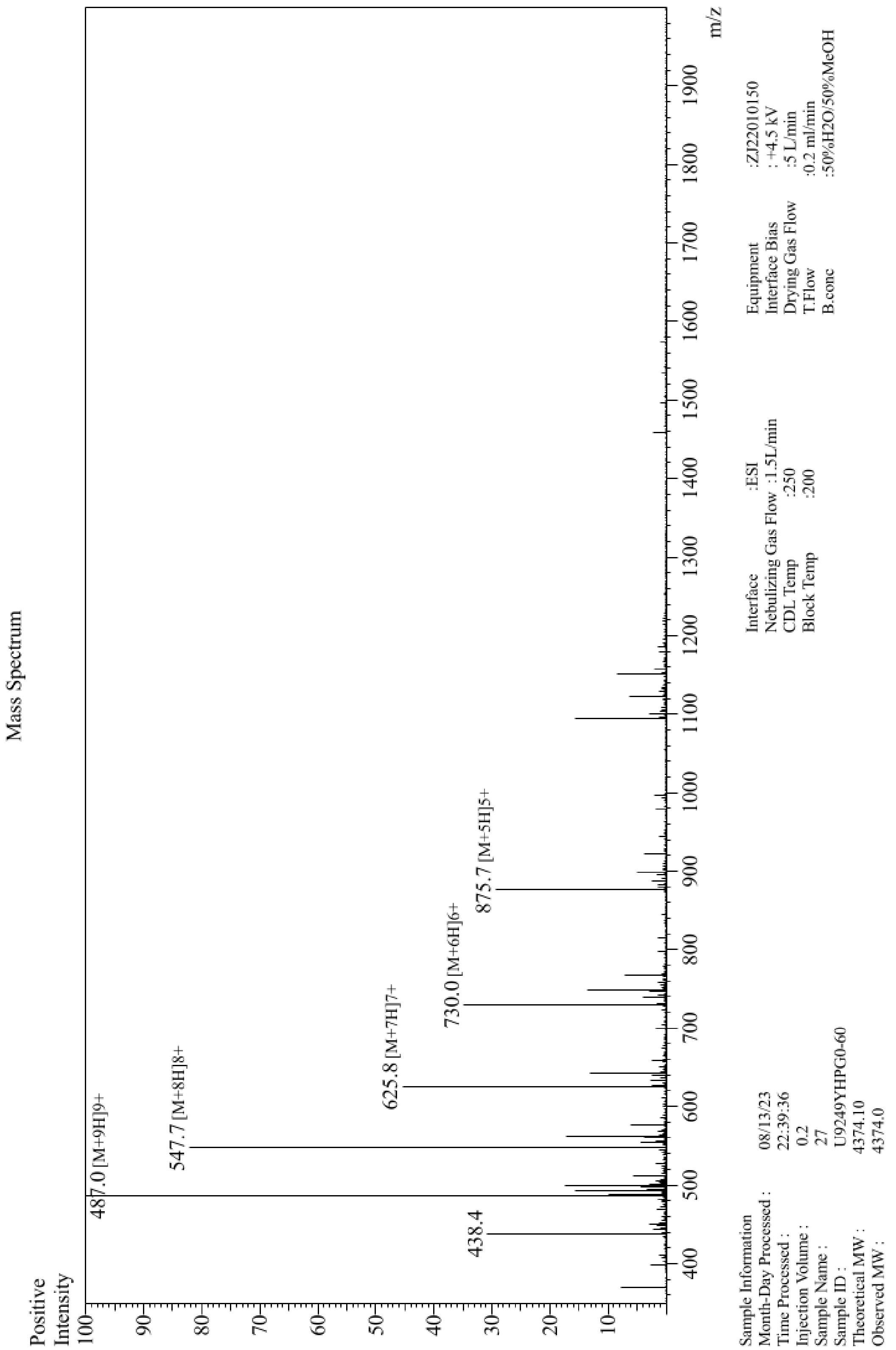

Figure S33. Mass spectrum: FITC-TAT-PSscan255. Data provided by GenScript.

Sample Name :28
Sample ID :U9249YHPG0-63
Time Processed :15:09:53
Month-Day-Year Processed :08/29/2023

Pump A : 0.065% trifluoroacetic in 100% water (v/v)
Pump B : 0.05% trifluoroacetic in 100% acetonitrile (v/v)
Total Flow:1 ml/min
Wavelength:220 nm
<<LC Time Program>>

Time	Module	Command	Value
0.01	Pumps	Pump A B.Conc	5
25.00	Pumps	Pump A B.Conc	65
25.01	Pumps	Pump A B.Conc	95
27.00	Pumps	Pump A B.Conc	95
27.01	Pumps	Pump A B.Conc	5
35.00	Pumps	Pump A B.Conc	5
35.01	Controller	Stop	

<<Column Performance>>
<Detector A>
Column :Inertsil ODS-SP 4.6 x 250 mm
Equipment: GR11010440

<Chromatogram>

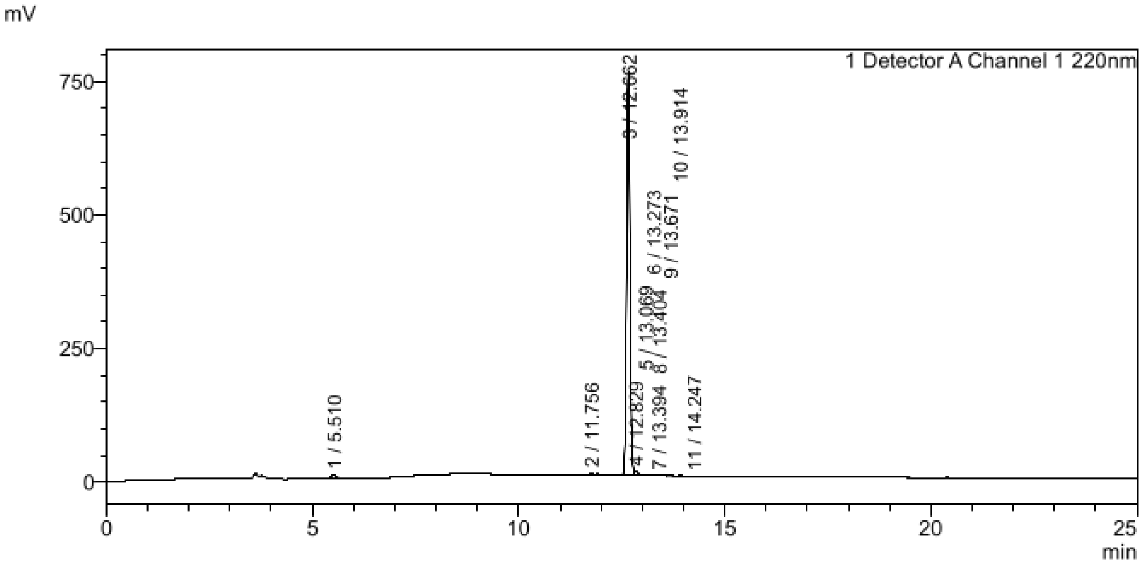

<Peak Table>

Detector A Channel 1 220nm

Peak#	Ret. Time	Area	Height	Area%
1	5.510	51626	6833	1.139
2	11.756	31545	4289	0.696
3	12.662	4369512	755925	96.411
4	12.829	43748	7034	0.965
5	13.069	13096	2124	0.289
6	13.273	12178	1870	0.269
7	13.394	3420	1156	0.075
8	13.404	2693	1154	0.059
9	13.671	1581	279	0.035
10	13.914	1756	290	0.039
11	14.247	1004	204	0.022
Total		4532159	781158	100.000

Figure S34. HPLC profile: TAT-PSscan255 (purity 96.4%). Data provided by GenScript.

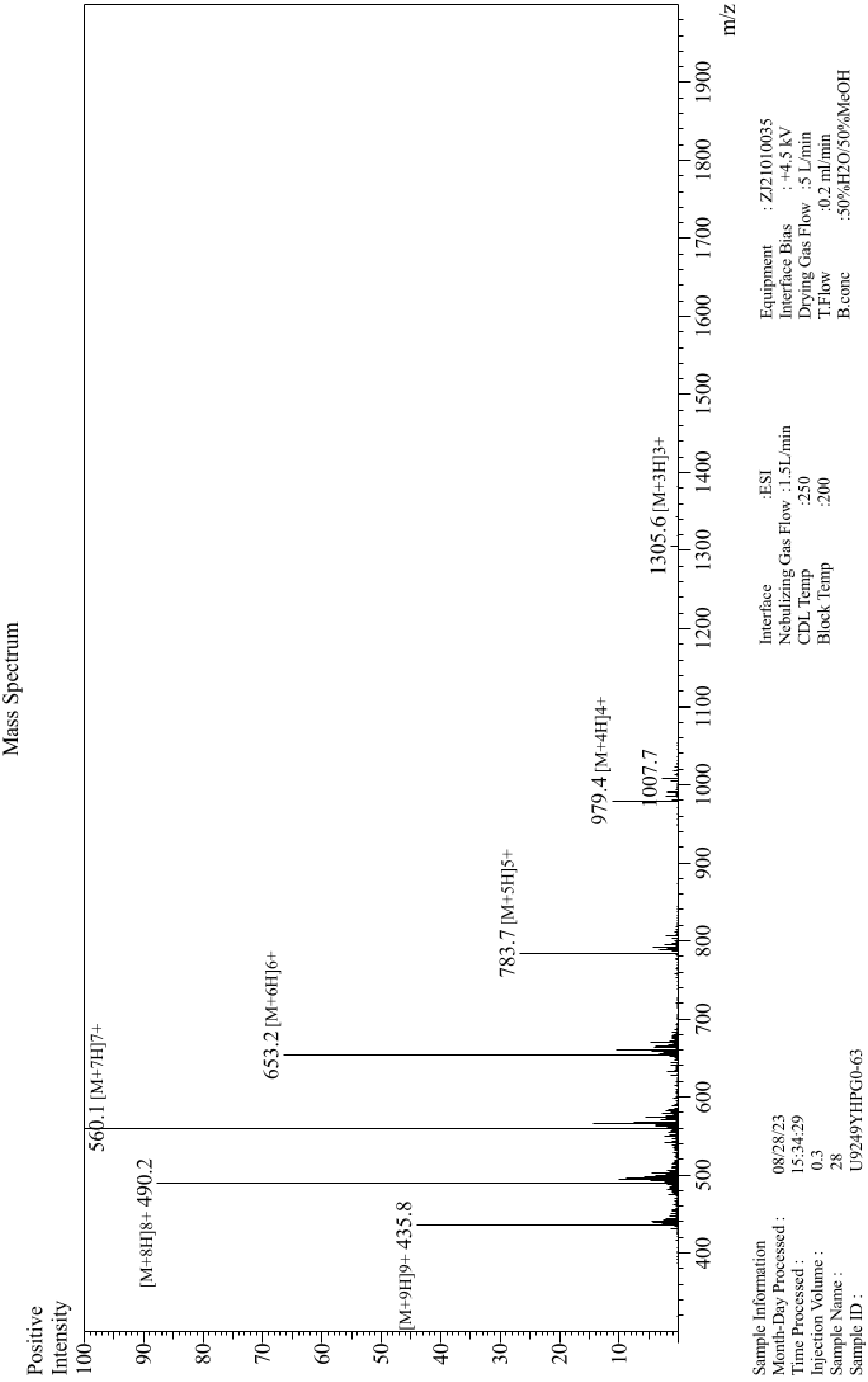

Figure S35. Mass spectrum: TAT-PSscan255. Data provided by GenScript.

Table S1. Results of FoldX analysis by the "PositionScan" macro [11-13] applied to the best Haddock [5] pose obtained for the Ship2-Sam/KRI3 complex [14]. All residues in the KRI3 peptide were individually substituted with the 20 natural amino acids, and ΔΔG ($\Delta G_{mut} - \Delta G_{WT}$) values were evaluated for all mutations. In each peptide position mutations associated with a more stabilizing effect (chosen threshold: ΔΔG < –0.3 Kcal/mol) and lower energy penalizations due to Van der Waals' clashes (chosen threshold: ΔVdW clashes ≤ 0.8 Kcal/mol) were selected. The most stabilizing substitutions are highlighted in bold on each peptide sequence.

**Reference Peptide**	**Sequence**	**PositionScan**	
**KRI3**	**1-KRIAYKRIAYKRIAY-15**		
**Mutation**	**Sequence**	**ΔΔG* (Kcal/mol)**	**ΔVdW* (Kcal/mol)**
**K1 → K#**	**K**RIAYKRIAYKRIAY	0.00	0.00
**K1 → M**	**M**RIAYKRIAYKRIAY	–0.305	–2.32
**I3 → I#**	KR**I**AYKRIAYKRIAY	0.00	0.00
**I3 → E**	KR**E**AYKRIAYKRIAY	–0.67	–0.03
**I3 → L**	KR**L**AYKRIAYKRIAY	–0.4	–0.02
**I3 → D**	KR**D**AYKRIAYKRIAY	–0.38	–0.03
**I3 → F**	KR**F**AYKRIAYKRIAY	–0.31	–0.02
**A4 → A#**	KRI**A**YKRIAYKRIAY	0.00	0.00
**A4 → W**	KRI**W**YKRIAYKRIAY	–0.69	–1.62
**A4 → M**	KRI**M**YKRIAYKRIAY	–0.35	–2.28
**R7 → R#**	KRIAYK**R**IAYKRIAY	0.00	0.00
**R7 → F**	KRIAYK**F**IAYKRIAY	–0.71	–0.003
**I8 → I#**	KRIAYKR**I**AYKRIAY	0.00	0.00
**I8 → M**	KRIAYKR**M**AYKRIAY	–0.52	–0.12
**I8 → L**	KRIAYKR**L**AYKRIAY	–0.48	–0.21
**Y10 → Y#**	KRIAYKRIA**Y**KRIAY	0.00	0.00
**Y10 → N**	KRIAYKRIA**N**KRIAY	–0.86	–0.03

*ΔΔG= $\Delta G_{mut} - \Delta G_{WT}$ and ΔVdW= $VdW_{mut} - VdW_{WT}$ where ΔG_{WT} and VdW_{WT} are the values associated with the starting Wild Type reference structure edited by the "PositionScan" macro.

#The "PositionScan" macro creates a different WT reference structure for the diverse amino acid positions.

Table S2. Results of the "BuildModel" macro of FoldX [11-13] applied to the best Haddock [5] pose obtained for the Ship2-Sam/KRI3 complex [14] by inserting the best (= the most stabilizing) peptide single mutations identified with the "PositionScan" macro. Best point-mutations in positions 1, 3, 4, 7, 8, and 10 (See Table S1) were combined to generate double, triple, four-, five- and six-fold mutants. Mutant peptides including the most stabilizing combinations of amino acid substitutions (chosen threshold: $\Delta\Delta G < -3.0$ Kcal/mol) and lacking significant increase in Van der Waals' clashes (chosen threshold: ΔVdW clashes ≤ 0.8 Kcal/mol) are reported in the table. Underlined peptides are those characterized by the lowest values of $\Delta\Delta G$ combined with significant decreases in ΔVdW clashes.

**Peptide name**	**Sequence**	**ΔΔG*** **(Kcal/mol)**	**ΔVdW*** **(Kcal/mol)**
PSscan217	**M**R**EM**YK**FM**AYKRIAY	–3.15	–0.56
PSscan245	**M**REAYK**FL**A**N**KRIAY	–3.05	–0.11
PSscan254	KR**EW**YK**FM**A**N**KRIAY	–3.22	0.39
PSscan255	KRE**W**YK**FL**A**N**KRIAY	–3.57	–1.08
PSscan258	KR**DW**YK**FM**A**N**KRIAY	–3.23	–1.97
PSscan266	**M**RE**W**YK**FM**A**N**KRIAY	–3.65	–0.38
PSscan323	KR**LW**YKR**M**A**N**KRIAY	–3.35	–0.04

*$\Delta\Delta G = \Delta G_{mut} - \Delta G_{WT}$ and $\Delta VdW = VdW_{mut} - VdW_{WT}$ where ΔG_{WT} and VdW_{WT} are the values associated with the reference structure edited by the "Build model" macro.

Table S3. Results of the "AnalyseComplex" macro of FoldX [11, 12]. Underlined peptides are those chosen for experimental studies.

**Peptide name**	**Sequence**	**ΔΔGbind#** **(Kcal/mol)**	**ΔVdWShip2-Sam@** **(Kcal/mol)**	**ΔVdWPeptide*** **(Kcal/mol)**
PSscan217	**M**R**EM**YK**FM**AYKRIAY	0.41	0.00	–0.02
PSscan245	**M**REAYK**FL**A**N**KRIAY	0.68	0.00	-0.20
PSscan254	KR**EW**YK**FM**A**N**KRIAY	0.76	0.00	0.13
PSscan255	KRE**W**YK**FL**A**N**KRIAY	–0.48	0.55	0.73
PSscan258	KR**DW**YK**FM**A**N**KRIAY	0.68	–0.61	–0.22
PSscan266	**M**RE**W**YK**FM**A**N**KRIAY	0.97	0.00	–0.56
PSscan323	KR**LW**YKR**M**A**N**KRIAY	–0.25	0.00	–0.22

Differences in the binding affinities of the mutated complex structures with respect to WT reference structures generated by the BuildModel Macro ($\Delta\Delta Gbind = \Delta Gbind_{mut} - \Delta Gbind_{WT}$).

@$\Delta VdW_{Ship2\text{-}Sam}$ represents the contribute to intramolecular VdW clashes at the interface due to the protein and evaluated as difference between that in mutated and corresponding WT complexes edited by the BuildModel Macro.

*$\Delta VdW_{Peptide}$ represents the contribute to intramolecular VdW clashes at the interface due to the peptide and evaluated as difference between that in mutated and reference WT complexes edited by the BuildModel Macro.

Tables S4. Peptide sequences analyzed in the manuscript.

Peptide	Sequence
PSscan255	Ac-KREWYKFLANKRIAY-NH_2
PSscan258	Ac-KRDWYKFMANKRIAY-NH_2
PSscan266	Ac-MREWYKFMANKRIAY-NH_2
Biotin-(Peg11)-PSscan255	Biotin-(Peg11)-KREWYKFLANKRIAY-NH_2
FITC-TAT-PSscan255	FITC-Ahx-βAla-GRKKRRQRRRPPQGGKREWYKFLANKRIAY-NH_2
TAT-PSscan255	Ac-βAla-GRKKRRQRRRPPQGGKREWYKFLANKRIAY-NH_2

Ac = N-terminal acetylation, NH_2 = C-terminal amidation, βAla = NH-CH_2CH_2-CO, FITC = Fluorescein-Isothiocyanate, Ahx = aminohexanoic linker

Table S5. Proton chemical shifts (ppm) of PSscan255 peptide in PBS/TFE (50/50 - v/v) at pH 7.03 and T 25°C. Chemical shifts were referenced with respect to internal TSP.

Residue	H_N	Hα	Hβ	Hγ	Others
1 K	7.57	4.26	1.48	1.36-1.82	Hδ 1.67<br>Hε 2.97<br>Acetyl 2.10
2 R	8.47	4.18	1.84	1.71	Hδ 3.23
3 E	8.82	4.16	2.07	2.40	
4 W	7.81	4.63	3.39		Hδ1 7.07<br>Hε1 9.76<br>Hε3 7.50<br>Hη2 7.02<br>Hζ2 7.51<br>Hζ3 7.23
5 Y	7.67	4.16	2.96-3.00		Hδ 7.04-7.06<br>Hε 6.87
6 K	7.82	4.01	1.90	1.39	Hδ 1.71<br>Hε 2.98
7 F	7.85	4.38	3.24-3.31		Hδ 7.21<br>Hε 7.31
8 L	8.02	3.96	1.49-1.75	1.63	Hδ 0.83-0.86
9 A	8.14	4.06	1.41		
10 N	7.89	4.55	2.82-2.88		Hδ 6.68-7.46
11 K	7.94	4.15	1.83	1.37	Hδ 1.61<br>Hε 2.86-2.93
12 R	8.00	4.20	1.92-1.96	1.72	Hδ 3.20
13 I	7.82	4.00	1.92	CH3 0.92<br>1.21-1.61	Hδ 0.90
14 A	7.86	4.22	1.34		
15 Y	7.75	4.52	3.00-3.16		Hδ 7.18-7.20<br>Hε 6.86

Table S6. Proton chemical shifts (ppm) of PSscan258 peptide in PBS/TFE (50/50 - v/v) at pH 7.03 and T 25°C. Chemical shifts were referenced with respect to internal TSP.

Residue	H_N	Hα	Hβ	Hγ	Others
1 K	7.91	4.32	1.72-1.88	1.44-1.50	Hδ 1.71<br>Hε 2.99<br>Acetyl 2.10
2 R	8.35	4.28	1.71-1.82	1.62	Hδ 3.14
3 D	8.21	4.62	2.67-2.77		
4 W	7.96	4.45	3.36		Hδ1 7.19<br>Hε1 9.79<br>Hε3 7.42<br>Hη2 7.21<br>Hζ2 7.49<br>Hζ3 7.02
5 Y	7.77	4.09	2.95-2.99		Hδ 7.09<br>Hε 6.90
6 K	7.78	3.98	1.86	1.39-1.45	Hδ 1.72<br>Hε 2.98
7 F	7.91	4.35	3.19-3.22		Hδ 7.19<br>Hε 7.33
8 M	8.12	4.19	1.97-2.00	2.32	Hε 2.03
9 A	8.16	4.08	1.41		
10 N	7.90	4.55	2.80-2.86		Hδ 6.67-7.44
11 K	7.91	4.15	1.85	1.40	Hδ 1.63<br>Hε 2.88-2.95
12 R	7.95	4.20	1.91-1.95	1.71-1.76	Hδ 3.21
13 I	7.83	4.02	1.91	CH3 0.91<br>1.20-1.61	Hδ 0.90
14 A	7.87	4.23	1.33		
15 Y	7.75	4.51	3.00-3.16		Hδ 7.18-7.21<br>Hε 6.86

Table S7. Proton chemical shifts (ppm) of PSscan266 peptide in PBS/TFE (50/50 - v/v) at pH 7.09 and T 25°C. Chemical shifts were referenced with respect to internal TSP.

Residue	H_N	Hα	Hβ	Hγ	Others
1 M	7.93	4.46	1.99-2.12	2.55-2.60	Acetyl 2.04
2 R	8.36	4.21	1.87	1.70-1.81	Hδ 3.26
3 E	8.54	4.16	2.11	2.40-2.43	
4 W	7.94	4.49	3.40-3.43		Hδ1 7.12<br>Hε1 9.64<br>Hε3 7.44<br>Hη2 7.21<br>Hζ2 7.48<br>Hζ3 7.01
5 Y	7.99	4.07	3.02-3.09		Hδ 7.09-7.12<br>Hε 6.88
6 K	7.83	3.97	1.90	1.39-1.53	Hδ 1.74<br>Hε 2.98
7 F	7.98	4.33	3.22-3.25		Hδ 7.16<br>Hε 7.31
8 M	8.18	4.12	1.88-1.91	2.16	Hε 1.99
9 A	8.21	4.06	1.39		
10 N	7.88	4.54	2.77-2.87		Hδ 6.66-7.45
11 K	7.90	4.12	1.80	1.37	Hδ 1.61<br>Hε 2.85-2.93
12 R	7.93	4.18	1.90-1.93	1.71	Hδ 3.20
13 I	7.81	4.00	1.92	CH3 0.92<br>1.20-1.61	Hδ 0.90
14 A	7.86	4.21	1.33		
15 Y	7.75	4.52	3.00-3.16		Hδ 7.17-7.20<br>Hε 6.86

Table S8. Structure statistics for 20 PSscan255 conformers. NMR structures were calculated in PBS/TFE (50/50 - v/v).

**Residual target function, Å^2**	0.15 ± 0.05
**Residual NOE violations**	0
**Number ≥ 0.1 Å***	0
**Residual angle violations**	0
**Atomic pairwise RMSD, Å**	
**Backbone atoms (all residues)**	0.73 ± 0.14
**Heavy atoms (all residues)**	1.34 ± 0.23
**PROCHECK analysis (all residues)#**	
**Residues in core regions**	59.6%
**Residues in allowed regions**	36.1%
**Residues in generous regions**	3.6%
**Residues in disallowed regions**	0.7%

*CYANA [15] mean violations
#PROCHECK-NMR [16] statistics

Table S9. Structure statistics for 20 PSscan258 conformers. NMR structures were calculated in PBS/TFE (50/50 - v/v).

**Residual target function, Å^2**	0.11 ± 0.01
**Residual NOE violations**	0
**Number ≥ 0.1 Å***	0
**Residual angle violations**	0
**Atomic pairwise RMSD, Å**	
**Backbone atoms (all residues)**	0.73 ± 0.14
**Heavy atoms (all residues)**	1.35 ± 0.22
**PROCHECK analysis (all residues)#**	
**Residues in core regions**	76.1%
**Residues in allowed regions**	23.2%
**Residues in generous regions**	0.7%
**Residues in disallowed regions**	0.0%

*CYANA [15] mean violations

#PROCHECK-NMR [16] statistics

Table S10. Structure statistics for 20 PSscan266 conformers. NMR structures were calculated in PBS/TFE (50/50 - v/v).

**Residual target function, Å^2**	0.17±0.02
**Residual NOE violations**	1
**Number ≥ 0.1 Å***	1
**Residual angle violations**	0
**Atomic pairwise RMSD, Å**	
**Backbone atoms (all residues)**	0.72±0.10
**Heavy atoms (all residues)**	1.27±0.13
**PROCHECK analysis (all residues)** #	
**Residues in core regions**	79.3%
**Residues in allowed regions**	17.9%
**Residues in generous regions**	2.9%
**Residues in disallowed regions**	0.0%

*CYANA [15] mean violations
#PROCHECK-NMR [16] statistics

Table S11. Intermolecular contact statistics for the Ship2-Sam/PSscan255 complex. Intermolecular H-bonds and non-bonded interactions refer to 5 among the 10 best Haddock [6] solutions (i.e., complex structures n.1, 3, 5, 6, 10). Regarding residue "1K", 2/5 non bonded contacts are provided by the acetyl protecting group.

PSscan255 residues	Number of H-bonds	Number of non-bonded interactions
1 K	8	5
2 R	4	2
3 E	0	0
4 W	2	10
5 Y	2	5
6 K	4	2
7 F	0	7
8 L	0	7
9 A	0	4
10 N	3	3
11 K	3	2
12 R	3	6
13 I	0	0
14 A	0	0
15 Y	0	0

References:

1. Micsonai, A.; Moussong, E.; Wien, F.; Boros, E.; Vadaszi, H.; Murvai, N.; Lee, Y.H.; Molnar, T.; Refregiers, M.; Goto, Y.; Tantos, A.; Kardos, J. BeStSel: webserver for secondary structure and fold prediction for protein CD spectroscopy. *Nucleic Acids Res.* **2022,** *50*(W1), W90-W98.
2. Kjaergaard, M.; Brander, S.; Poulsen, F.M. Random coil chemical shift for intrinsically disordered proteins: effects of temperature and pH. *J. Biomol. NMR* **2011,** *49*(2), 139-149.
3. Farmer, B.T., 2nd; Constantine, K.L.; Goldfarb, V.; Friedrichs, M.S.; Wittekind, M.; Yanchunas, J., Jr.; Robertson, J.G.; Mueller, L. Localizing the NADP+ binding site on the MurB enzyme by NMR. *Nat. Struct. Biol.* **1996,** *3*(12), 995-7.
4. Leone, M.; Cellitti, J.; Pellecchia, M. NMR studies of a heterotypic Sam-Sam domain association: the interaction between the lipid phosphatase Ship2 and the EphA2 receptor. *Biochemistry* **2008,** *47*(48), 12721-12728.
5. de Vries, S.J.; van Dijk, M.; Bonvin, A.M. The HADDOCK web server for data-driven biomolecular docking. *Nat. Protoc.* **2010,** *5*(5), 883-897.
6. Honorato, R.V.; Trellet, M.E.; Jimenez-Garcia, B.; Schaarschmidt, J.J.; Giulini, M.; Reys, V.; Koukos, P.I.; Rodrigues, J.; Karaca, E.; van Zundert, G.C.P.; Roel-Touris, J.; van Noort, C.W.; Jandova, Z.; Melquiond, A.S.J.; Bonvin, A. The HADDOCK2.4 web server for integrative modeling of biomolecular complexes. *Nat. Protoc.* **2024**.
7. Laskowski, R.A.; Swindells, M.B. LigPlot+: multiple ligand-protein interaction diagrams for drug discovery. *J. Chem. Inf. Model.* **2011,** *51*(10), 2778-86.
8. Wallace, A.C.; Laskowski, R.A.; Thornton, J.M. LIGPLOT: a program to generate schematic diagrams of protein-ligand interactions. *Protein Eng.* **1995,** *8*(2), 127-34.
9. Jumper, J.; Evans, R.; Pritzel, A.; Green, T.; Figurnov, M.; Ronneberger, O.; Tunyasuvunakool, K.; Bates, R.; Zidek, A.; Potapenko, A.; Bridgland, A.; Meyer, C.; Kohl, S.A.A.; Ballard, A.J.; Cowie, A.; Romera-Paredes, B.; Nikolov, S.; Jain, R.; Adler, J.; Back, T.; Petersen, S.; Reiman, D.; Clancy, E.; Zielinski, M.; Steinegger, M.; Pacholska, M.; Berghammer, T.; Bodenstein, S.; Silver, D.; Vinyals, O.; Senior, A.W.; Kavukcuoglu, K.; Kohli, P.; Hassabis, D. Highly accurate protein structure prediction with AlphaFold. *Nature* **2021,** *596*(7873), 583-589.
10. Varadi, M.; Anyango, S.; Deshpande, M.; Nair, S.; Natassia, C.; Yordanova, G.; Yuan, D.; Stroe, O.; Wood, G.; Laydon, A.; Zidek, A.; Green, T.; Tunyasuvunakool, K.; Petersen, S.; Jumper, J.; Clancy, E.; Green, R.; Vora, A.; Lutfi, M.; Figurnov, M.; Cowie, A.; Hobbs, N.; Kohli, P.; Kleywegt, G.; Birney, E.; Hassabis, D.; Velankar, S. AlphaFold Protein Structure Database: massively expanding the structural coverage of protein-sequence space with high-accuracy models. *Nucleic Acids Res.* **2022,** *50*(D1), D439-D444.
11. Delgado, J.; Radusky, L.G.; Cianferoni, D.; Serrano, L. FoldX 5.0: working with RNA, small molecules and a new graphical interface. *Bioinformatics* **2019,** *35*(20), 4168-4169.
12. Schymkowitz, J.; Borg, J.; Stricher, F.; Nys, R.; Rousseau, F.; Serrano, L. The FoldX web server: an online force field. *Nucleic Acids Res.* **2005,** *33*(Web Server issue), W382-8.
13. Kiel, C.; Serrano, L. Structure-energy-based predictions and network modelling of RASopathy and cancer missense mutations. *Mol. Syst. Biol.* **2014,** *10*(5), 727.
14. Vincenzi, M.; Mercurio, F.A.; Di Natale, C.; Palumbo, R.; Pirone, L.; La Manna, S.; Marasco, D.; Pedone, E.M.; Leone, M. Targeting Ship2-Sam with peptide ligands: Novel insights from a multidisciplinary approach. *Bioorg. Chem.* **2022,** *122*, 105680.
15. Herrmann, T.; Guntert, P.; Wuthrich, K. Protein NMR structure determination with automated NOE assignment using the new software CANDID and the torsion angle dynamics algorithm DYANA. *J. Mol. Biol.* **2002,** *319*(1), 209-227.
16. Laskowski, R.A.; Rullmannn, J.A.; MacArthur, M.W.; Kaptein, R.; Thornton, J.M. AQUA and PROCHECK-NMR: programs for checking the quality of protein structures solved by NMR. *J. Biomol. NMR* **1996,** *8*(4), 477-486.

Note: reference 13 corresponds to reference 86 in the Main Text.
